# MiR-301b-3p promotes breast cancer development through inhibiting the expression of transforming growth factor-beta receptor 2

**DOI:** 10.7717/peerj.18324

**Published:** 2024-11-05

**Authors:** Jian Lou, Xueni Liu, Yanru Xie, Minhua Wu, Weibo Mao, Xiaozhen Ying

**Affiliations:** 1 Tumor Center, Lishui Central Hospital, Lishui, China; 2 Pathology Department, Lishui Central Hospital, Lishui, China

**Keywords:** MiR-301b-3p-inhibitor, Transforming growth factor-beta receptor 2 (TGFBR2), Transwell assay, Breast cancer, Antagonism of miR-301b-3p and TGFBR2

## Abstract

**Background:**

Breast cancer (BC) is a serious health threat to the patients. The present work explored the mechanism of miR-301b-3p and transforming growth factor-beta receptor 2 (TGFBR2 ) in affecting BC progression.

**Methods:**

The miR-301b-3p-inhibitor and si-TGFBR2 solution were added to the DEME/F12 medium to culture the BC and normal breast epithelial cell lines to prepare negative control, miR-301b-3p-IN and miR-301b-3p-IN+si-TGFBR2 in the two types of cell lines. The relative expression of target genes and the interference effect were analyzed by quantitative real-time PCR (qRT- PCR). Cell viability was detected applying cell counting kit-8 (CCK-8) assay. Transwell and wound healing assay were conducted to evaluate the invasion and migration of BC cells after miR-301b-3p inhibition. Additionally, cell apoptosis and the expression STAT protein were measured by flow cytometry and Western blot, respectively

**Results:**

The qRT-PCR results showed that miR-301b-3p were high-expressed but the level of TGFBR2 was significantly inhibited in BC cells. The miR-301b-3p-inhibitor significantly downregulated the expression of miR-301b-3p and upregulated that of TGFBR2. Meanwhile, inhibition of miR-301b-3p suppressed the cell viability, invasion, and migration of BC cells, which, however, were restored by the inhibition of TGFBR2. MiR-301b-3p conferred anti-apoptosis ability to BC cells, while TGFBR2 promoted apoptosis of BC cells through producing an antagonistic effect with miR-301b-3p. We found that miR-301b-3p played a crucial role in the phosphorylation of STAT1 and STAT3 to promote BC progression.

**Conclusion:**

The present findings demonstrated that miR-301b-3p played a crucial role in promoting BC cell growth, invasion and migration and anti-apoptosis, and that targeting TGFBR2 could inhibit the tumor-promoting effect of miR-301b-3p.

## Introduction

Breast cancer (BC) is a highly heterogeneous and the most frequent female malignancy (Sung et al., 2021) characterized by high incidence rate and metastasis (Kim et al., 2018). Statistics reported that the estimated new cases and deaths of female BC patients reached 297,790 and 43,170,respectively, in 2023 (Siegel et al., 2023). In recent years, immunotherapy has been widely applied to treat BC. Immune checkpoint inhibitors (ICIs), including PD-1 inhibitor pembrolizumab and PD-L1 inhibitor atezolizumab, have significantly improved the survival of BC (Schmid et al., 2018; Cortes et al., 2022; Akhtar et al., 2023). However, the heterogeneity and distinctive biological characteristics of BC still hinder a successful treatment, and recurrence and metastasis will occur to numerous patients (Ray & Mukherjee, 2024). Currently, major challenges in BC treatment include resistance to chemotherapy and radiotherapy as well as systemic immune-related side effects (Liu et al., 2023). As a result, novel therapeutic targets and more reliable prognostic indicators are needed to facilitate the management of BC.

In multiple organisms, microRNAs (miRNAs) are defined as highly conserved small non-coding single-stranded RNAs with around 22 nucleotides in length (Nicolas et al., 2009) and act as post-transcriptional regulators (Diener, Keller & Meese, 2022) that bind to the 3′-untranslated regions (UTRs) (Sabbaghian et al., 2018) of the messenger RNAs (mRNAs) to degrade or suppress the mRNAs at translational level (Bartel, 2009). The expression changes of miRNAs are pivotal to the regulation of cellular genetic networks and signaling cascades (Diener, Keller & Meese, 2022). For example, altered miRNAs expression could modify proteins as part of pathological changes in some diseases (Subramanian & Steer, 2019), suggesting that miRNAs could be considered as the hallmarks of diseases and effective therapeutic targets (Huang, 2017). Most mature miRNA sequences originated from the exons or introns of non-coding RNAs and the introns of pre-miRNA (Kalla et al., 2015), which are cleaved into stem-loop structure in nucleus by Drosh (García-López, Brieño Enríquez & Del Mazo, 2013) and exported to the cytoplasm by Exportin 5 (Wu et al., 2018) to form small double-stranded RNAs (dsRNAs) *via* Dicer (Ha & Kim, 2014) and assemble into RNA-induced silencing complex (RISC) to participate in the cell proliferation, differentiation, apoptosis processes (Iwakawa & Tomari, 2022). In BC, miR-301b-3p functions as a pro-cancer factor to promote the cell proliferation, invasion and migration *via* targeting NR3C2 (Fan et al., 2021). Moreover, upregulating miR-301b-3p can facilitate the tumor proliferation and migration by targeting HOXA5, HOXB1, and DLC1 in BC (Lu et al., 2021), colorectal cancer (Xiong et al., 2021), lung adenocarcinoma, respectively (Liu et al., 2021). Study showed that hypoxia upregulates miR-301b-3p to promote the occurrence, metastasis and progression of prostate cancer by targeting LRP1B (Zheng & Bai, 2019). Overexpressed miR-301b-3p inhibits TXNIP in mesenchymal stem cells to enhance the resistance of gastric carcinoma to multiple drugs (Zhu et al., 2023). These findings showed that miR-301b-3p paly pro-cancer role *via* different mechanisms in a variety of cancers. In addition, Zhou et al. (2023) observed significantly upregulated miR-301b-3p in BC tissues in comparison to normal para-carcinoma tissue, demonstrating that miR-301b-3p can enhance tumor progression and serve as potential marker to indicate BC progression. Therefore, elucidating the carcinogenic mechanism of miR-301b-3p could help discover effective targets for BC treatment.

TGFBR2 is involved in regulating TGF-β signaling during tumorigenesis (Huang et al., 2014). Loss or downregulation of TGFBR2 expression leads to uncontrolled growth of tumor cells. Some researchers have shown that the mRNA and protein expression levels of TGFBR2 are significantly lowered in BC tissues than in adjacent normal tissues. They also found that low TGFBR2 expression is closely associated with malignant pathologic features and prognosis in BC (Wei et al., 2015). Moreover, overexpressed TGFBR2 inhibits the cervical cancer cell migration and proliferation through targeting SMAD4 to block the Hedgehog signaling pathway (Yuan, Yi & Yang, 2020). Another study revealed a dual effect of TGFBR2 on BC as it can inhibit primary tumor and promote the growth of aggressive tumor (Vitiello et al., 2019). Specifically, Chen et al. (2021) found that ADAMTS9-AS1 could chelate miR-301b-3p *via* the TGFBR2/JAK-STAT pathway, which in turn suppresses the onset and progression of BC. At present, many studies have confirmed TGFBR2 as an important tumor-suppressive factor, while the mechanism of its effect on BC development remained unclear.

The present study applied miR-301b-3p-inhibitor and small interfering-TGFBR2 (si-TGFBR2) to produce miR-301b-3p-IN and miR-301b-3p-IN+si-TGFBR2 interfering cells in the BC cell lines (MCF-10A and MCF-7) to analyze the role of miR-301b-3p and TGFBR2 in BC development. The expressions of TGFBR2 and miR-301b-3pin BC and normal breast epithelial cells. Next, the BC cell viability and invasion, migration and apoptosis and cancer pathway-associated proteins were measured after miR-301b-3p inhibition. In conclusion, this study discovered novel targets for the clinical management of BC.

## Materials and Methods

### Cell culture and RNA silencing

Human BC cell lines containing MCF-7 and MDA-MB-231 and normal human breast epithelial cell lines MCF-10A (Mosayyebi et al., 2021) were obtained from the COBIOER corp. (Nanjing, China). Dulbecco’s modified eagle/F12 medium (DEME/F12, 1:1, Thermo Fisher Scientific, Waltham, MA, USA) added with 10% fetal bovine serum (FBS, Thermo Fisher) were employed for cell culture. The environment was set in a humidified incubator at 37 °C with 5% CO_2_ (Tao et al., 2019).

### Establishment of BC cell lines with miR-301b-3p and TGFBR2 silencing

The miR-301b-3p-inhibitor and si-TGFBR2 were purchased from the Sigon corp. (Suzhou, China) as the si-TGFBR2 sequence 5′GTGTAATGATATGTGCATATTTA3′ could target TGFBR2 (Ma et al., 2014). MiR-301b-3p-inhibitor and si-TGFBR2 reagent were dissolved in sterile water and prepared to required concentration according to the manufacturer’s instruction. Next, transfection of miR-301b-3p-inhibitor and si-TGFBR2 solution into MCF-7, MDA-MB-231 and MCF-10A was performed accordingly (Tao et al., 2019). After that, the cells were cultured for subsequent experiments.

### RNA extraction and qRT-PCR analysis

Extraction of RNA and synthesis of cDNA from the three types of cultured cells were performed using the TRIzol reagent (Rio et al., 2010) (15596026 Thermo Fisher, Waltham, MA, USA) and PrimeScript RT-PCR kit (Takara Bio, Shiga, Japan). Subsequently, qRT-RCR was conducted with the use of FastStart Universal SYBR Green Master kit and LightCycler 480 PCR System (Roche, Boston, MA, USA). The reaction mixture (20 µL) consisted of cDNA template (2 µL), PCR master mixture (10 µL), forward and reverse primers (0.5 µL) and appropriate water. Then, the reaction began with initial denaturation at 95 °C for 30 s 9 (s), followed by denaturation at 94 °C for 15 s, annealing at 56 °C for 30 s and extension at 72 °C for 20 s, for a total of 45 cycles (Amuthalakshmi, Sindhuja & Nalini, 2022). The 2^−ΔΔCT^ method (Maren et al., 2023) was used to detect the expressions of TGFBR2 and miR-301b-3p based on threshold cycle (CT) value. GAPDH and U6 served as internal reference genes for TGFBR2 and miR-301b-3p, respectively. Three technical replicates were set for each sample contained and the target gene primer sequence was shown in Table 1.

**Table 1:** The sequences of primer pairs for qRT-PCR.

Gene	Forward primer sequence (5′–3′)	Reverse primer sequence (5′–3′)
miR-301b-3p	CAGTGCTCTGACGAGGTTG	TGTCCCAGATGCTTTGACA
TGFBR2	TCGAAAGCATGAAGGACAACG	AGCACTCAGTCAACGTCTCAC
U6	CTCGCTTCGGCAGCACAT	TTTGCGTGTCATCCTTGCG
GAPDH	GTCTCCTCTGACTTCAACAGCG	ACCACCCTGTTGCTGTAGCCAA

### Cell viability

The viability of cells was determined using CCK-8 assay (Xu et al., 2021) (Beyotime, Haimen, China), during which the WST-8 were reduced by mitochondrial dehydrogenase to orange-yellow formazan to indirectly indicate the number of living cells. Briefly, the cells (1 × 10^4^) cells/well) were cultured in 96-well plates for 0, 24, 48 and 72 h, and the CCK-8 solution was added at the indicated time points to each well and incubated for 2 h at 37 °C. After that, the absorbance of mixed solutions was recorded using the microplate reader (Thermo Fisher Scientific) under 450 nm. Each experiment was repeated in triplicate.

### Transwell assay

Transwell assays (Flores-Pérez et al., 2018) were conducted to assess the abilities of MCF-7 and MDA-MB-231 cells to migrate and invade. After collection, trypsin digestion and counting, the cells (5 × 10^4^) were added into the upper chambers coated or uncoated with Matrigel (BD Biosciences, Franklin Lakes, NJ, USA) in 100 µL FBS-free medium for invasion or migration test. The lower chamber was supplemented with 600 µL DMEM medium. After 24-h incubation, 4% paraformaldehyde was used to fix the cells, which were then dyed with 0.1% crystalline violet and quantified under a light microscope. Each cell line was performed with three experiments.

### Wound healing assay and flow cytometry

Monolayer cells at a density of 2 ×10^4^ cells/well were seeded into 6-well plates and a wound was created with the sterile 200  µL pipette tips. Then the DMEM medium were replaced by serum-free medium (SFM, Cat#12338018 Thermo Fisher Scientific) for 24-h incubation, after which the width of the wound was recorded (Martinotti & Ranzato, 2020). Cell apoptosis was detected by conducting flow cytometry (Li, 2017). Briefly, cultured cells were harvested using trypsin and resuspended at a concentration of 1 × 10^5^/200 µL in phosphate buffer saline (PBS). Subsequently, Annexin V-FITC and PI solution were used for cell staining on ice in the dark for 30 min (min). The samples were detected by A BD FACS Calibur flow cytometer (BD, USA).

### Western blot

After washing with PBS to remove residual medium, the cell were lysed by RIPA buffer (Solarbio, Beijing, China) for 15 min for measuring the concentration (Tang et al., 2021). The protein samples were added to SDS loading buffer and incubated at 100 °C for 15 min. After separating the proteins with SDS-PAGE, the proteins were moved to polyvinylidene difluoride (PVDF) film and blocked by 5% non-fat dry milk for 1 h. Next, the film was incubated with primary antibodies overnight (Tang et al., 2021) and then with the secondary antibodies for further incubation for 2 h. Finally, a chemiluminescence imager was employing to visualize the protein bands (Tang et al., 2021). The antibodies were shown in Table 2.

**Table 2:** The information of secondary antibodies for western blot.

Maker	Brand	Lot Numbers
STAT1	Proteintech	10144-2-AP
P-STAT1	Proteintech	28977-1-AP
STAT3	Proteintech	10253-2-AP
P-STAT3	Abcam	ab267373
STAT6	Proteintech	51073-1-AP
P-STAT6	Abcam	ab263947

### Statistical analyses

All statistical analyses were performed in the SPSS software (version 26.0). The expressions of TGFBR2 and miR-301b-3p derived from the BC cell lines were analyzed with one-way ANOVA. Expression difference between two types of cells was analyzed by the Wilcoxon matched-pairs test. All the experiments were set with three repetitions of technique. A *p*-value < 0.05 was defined as a statistical significance. SangerBox (http://sangerbox.com/home.html) provided certain analytical assistance for this study.

**Figure 1:**
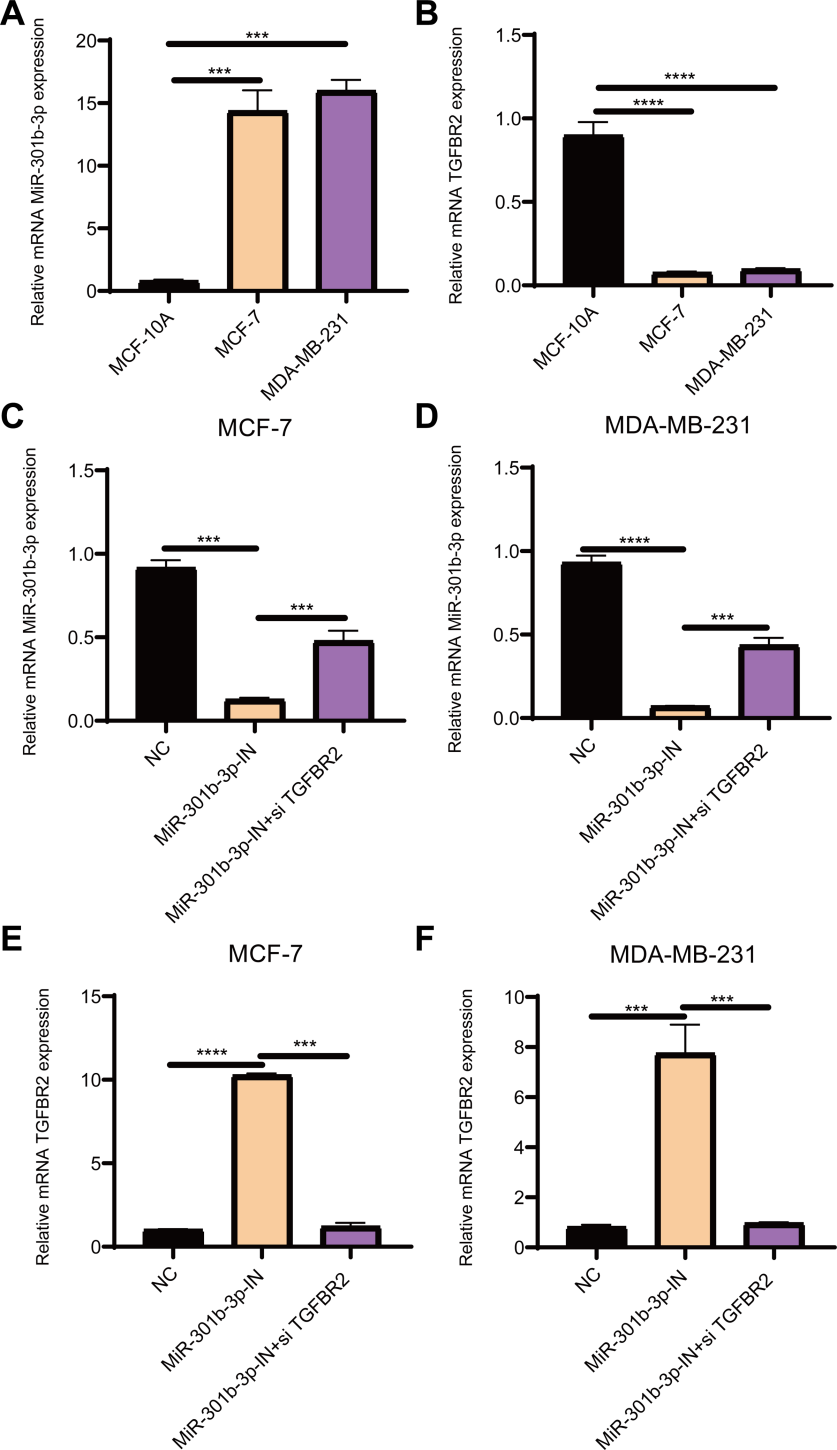
The relative expression analysis of miR-301b-3p and TGFBR2 genes. (A) qRT-PCR for miR-301b-3p expression in MCF-10A, MCF-7 and MDA-MB-231 cell lines. (B) qRT-PCR for TGFBR2 expression in the three cell lines. (C) qRT-PCR for miR-301b-3p expression in MCF-7 cell lines without silencing, with miR-301b-3p-silencing and miR-301b-3p+TGFBR2 silencing. (D) qRT-PCR for miR-301b-3p expression in MDA-MB-231 cell lines without silencing, with miR-301b-3p-silencing and miR-301b-3p+TGFBR2 silencing. (E) qRT-PCR for TGFBR2 expression in MCF-7 cell lines without silencing, with miR-301b-3p-silencing and miR-301b-3p+TGFBR2 silencing. (F) qRT-PCR for TGFBR2 expression in MDA-MB-231 cell lines without silencing, with miR-301b-3p-silencing and miR-301b-3p+TGFBR2 silencing . Data were shown as mean ± SD and ^∗^ ≤ 0.05, ^∗∗^ ≤ 0.01, ^∗∗∗^ ≤ 0.001, ^∗∗∗∗^ ≤ 0.0001.

## Results

### MiR-301b-3p was overexpressed and TGFBR2 was suppressed in BC cells

The qRT-PCR results demonstrated that the level of miR-301b-3p was elevated in the two BC cell lines (Fig. 1A), while that of TGFBR2 was suppressed in BC cells but elevated in normal cells (Fig. 1B). Subsequently, the level of miR-301b-3p in the two BC cell lines silenced with miR-301b-3p inhibitor or miR-301b-3p inhibitor+si TGFBR2 was measured. In the MCF-7 cell lines, the expression level of miR-301b-3p was remarkably inhibited in silencing groups than in negative control (NC) group, but was slightly upregulated in miR-301b-3p-IN+si TGFBR2 silencing group (Fig. 1C). Meanwhile, similar results were observed in MDA-MB-231 cell lines (Fig. 1D), indicating an effective silencing by miR-301b-3p inhibitor. However, compared to NC groups, the expression of TGFBR2 was remarkably upregulated in the MCF-7 (Fig. 1E) and MDA-MB-231 cell lines (Fig. 1F) in the in miR-301b-3p silencing groups. Here, the data revealed a tumor-promoting role of miR-301b-3p and a tumor-suppressive role of TGFBR2 in BC, suggesting that miR-301b-3p can enhance BC development through suppressing TGFBR2.

### MiR-301b-3p was involved the proliferation of BC cells

**Figure 2:**
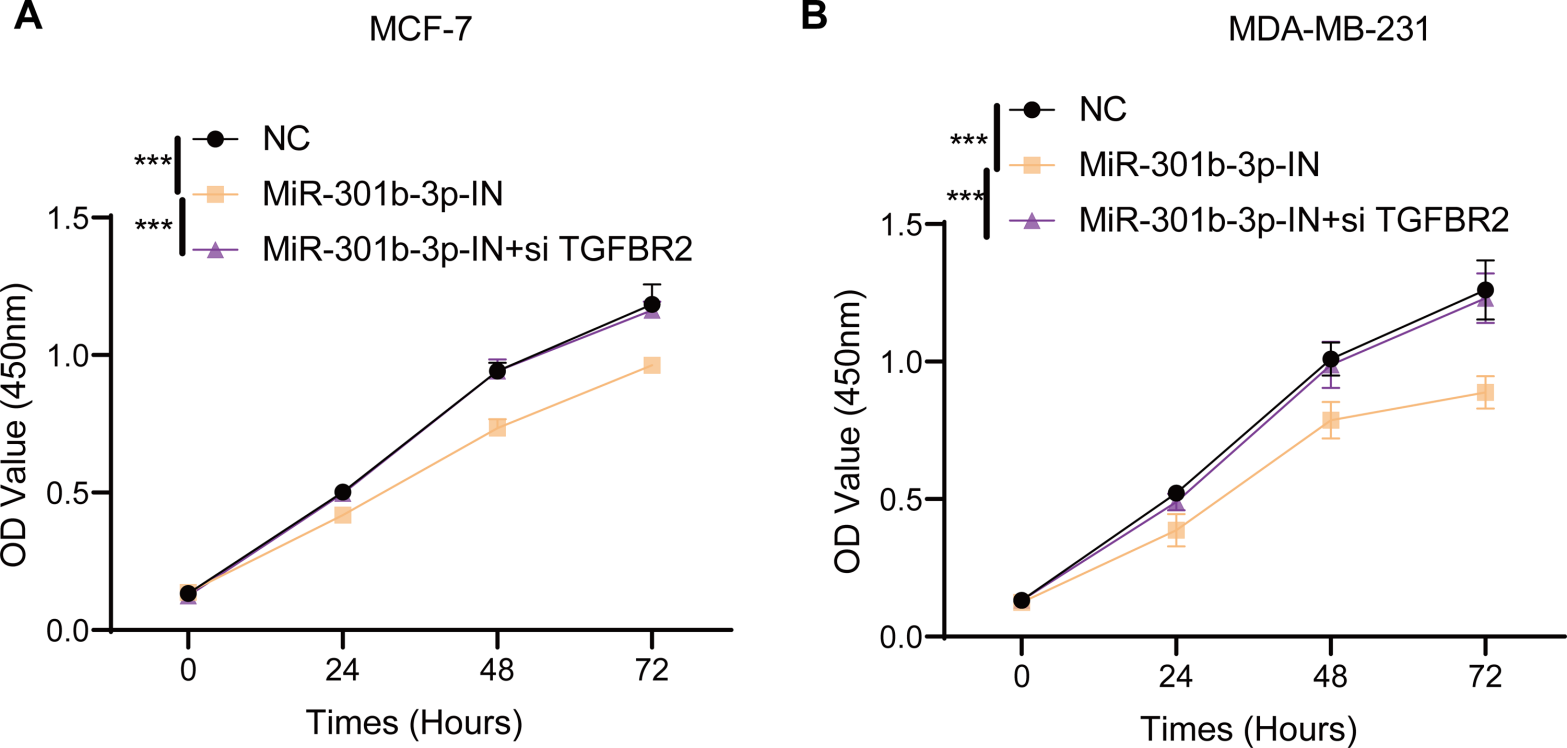
Cell viability assay in different silenced groups. (A) CCK8 assay for no silencing, miR-301b-3p-silencing and miR-301b-3p+TGFBR2 silencing group in MCF-7 cell lines. (B) CCK8 assay for no silencing, miR-301b-3p-silencing and miR-301b-3p+TGFBR2 silencing group in MDA-MB-231 cell lines. Data were shown as mean ± SD and *** represents *P* ≤ 0.001.

In the MCF-7 cell lines, the CCK-8 results presented that the cell viability of BC cells in the miR-301b-3p-IN silencing groups were significantly reduced than the NC and miR-301b-3p-IN+si TGFBR2 silencing groups. In addition, the BC cells showed highly similar cell viability patterns in the NC and miR-301b-3p-IN+si TGFBR2 silencing groups (Fig. 2A). Also, consistent results in the MDA-MB-231 cells were also observed from the corresponding cell viability assay (Fig. 2B). These data indicated that the miR-301b-3p gene was involved in the proliferation of BC cells, and that silencing miR-301b-3p suppressed BC cell viability. In addition, the BC cells showed a normal cell viability in miR-301b-3p-IN+si TGFBR2 silencing groups, suggesting an antagonistic correlation between TGFBR2 and miR-301b-3p during BC progression.

### MiR-301b-3p enhanced the BC cell invasion and migration

**Figure 3:**
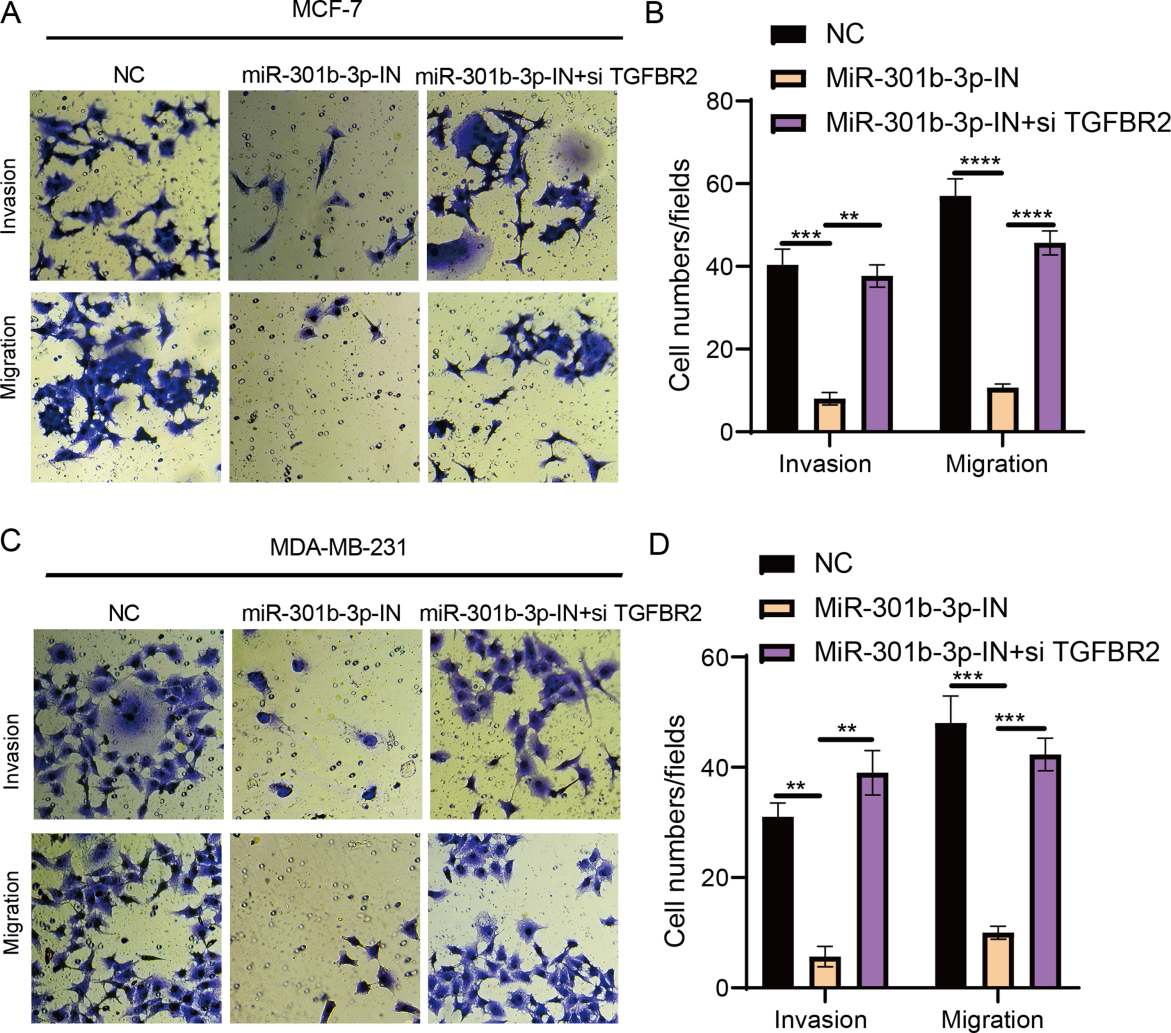
Cell invasion and migration assay in different silenced groups.. (A) Trans-well assay for no silencing, miR-301b-3p-silencing and miR-301b-3p+TGFBR2 silencing group in MCF-7 cell lines. (B) The cell count statistics for invasion and migration in MCF-7 cell lines. (C) Trans-well assay for no silencing, miR-301b-3p-silencing and miR-301b-3p+TGFBR2 silencing group in MDA-MB-231 cell lines. (D) The cell count statistics for invasion and migration in MDA-MB-231 cell lines. Data were shown as mean ± SD and ^∗^ ≤ 0.05, ^∗∗^ ≤ 0.01, ^∗∗∗^ ≤ 0.001, ^∗∗∗∗^ ≤ 0.0001.

**Figure 4:**
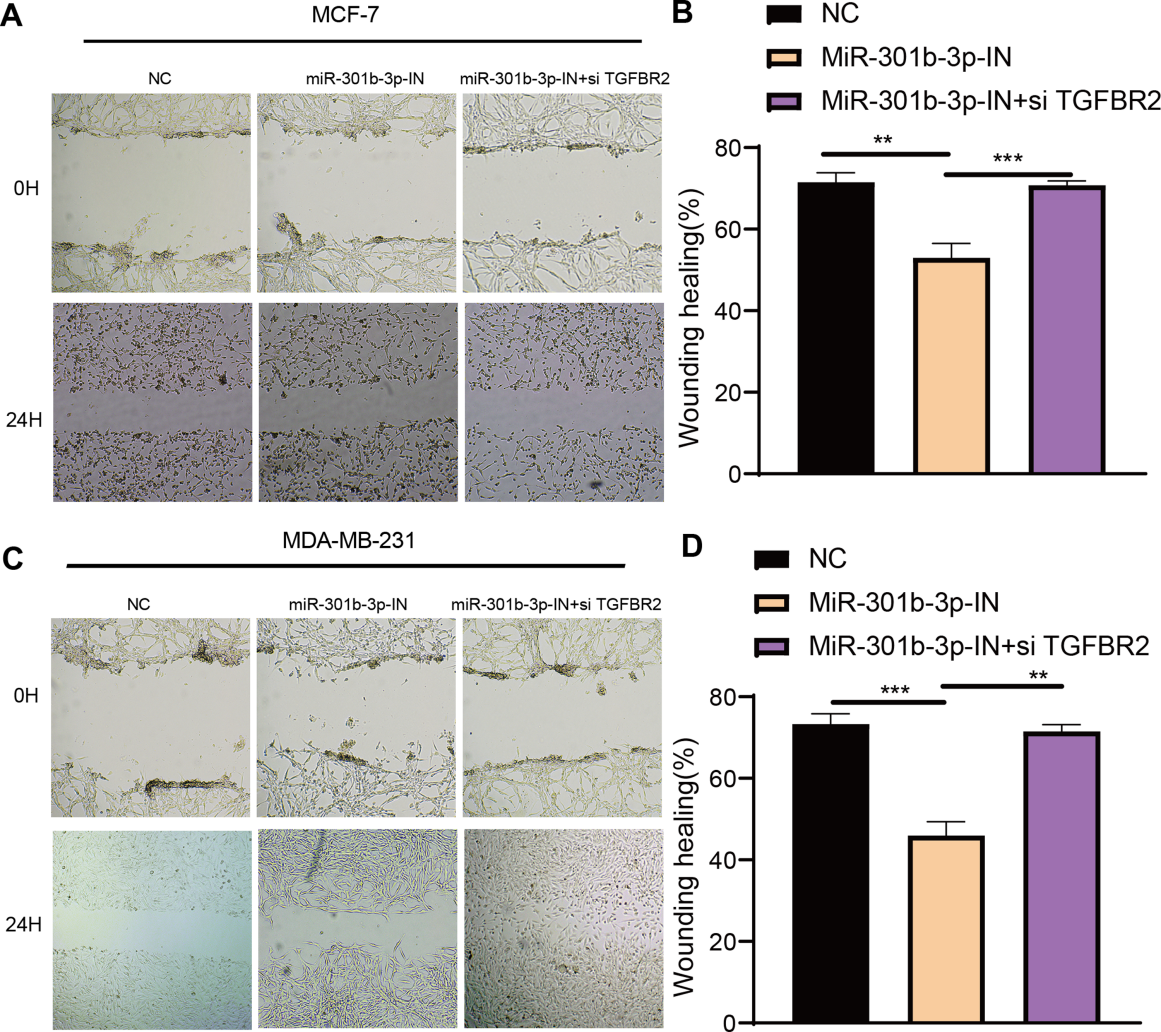
Wound healing assay in different silenced groups. (A, B) Wound healing assay for no silencing, miR-301b-3p-silencing and miR-301b-3p+TGFBR2 silencing group in MCF-7 cell lines. (C, D) Wound healing assay for no silencing, miR-301b-3p-silencing and miR-301b-3p+TGFBR2 silencing group in MDA-MB-231 cell lines. Data were shown as mean ± SD and ^∗^ ≤ 0.05, ^∗∗^ ≤ 0.01, ^∗∗∗^ ≤ 0.001, ^∗∗∗∗^ ≤ 0.0001.

The transwell and wound healing assay were used to investigate the role of TGFBR2 and miR-301b-3p in BC cell invasion and migration. In the MCF-7 cell lines, the BC cells in the NC groups had stronger ability to invade through the Matrigel and there were more blue cells. However, the BC cell invasion and migration were remarkably suppressed and there were only few cells stained blue after miR-301b-3p-IN silencing. Simultaneous silencing of TGFBR2 and miR-301b-3p partially restored the BC cell invasion and migration ability (Fig. 3A). The NC groups had the highest number of cells, the miR-301b-3p-IN groups had the lowest number of invaded and migrated cells, and the miR-301b-3p-IN+si TGFBR2 groups had relatively high number of cells after the inhibiting the expression of TGFBR2 (Fig. 3B). Additionally, the consistent results in the MDA-MB-231 cells were observed, as we found that silencing miR-301b-3p-IN inhibited the invasion and migration of BC cells, which were restored after silencing TGFBR2 (Fig. 3C). The only difference was that the miR-301b-3p-IN+si TGFBR2 groups contained the highest number of invaded cells (Fig. 3D), indicating a critical role of TGFBR2 in suppressing BC cell invasion. In wound healing assay, the invasion of MCF-7 cells (Figs. 4A, 4B) and MDA-MB-231 cells (Figs. 4C, 4D) was inhibited when miR-301b-3p was silenced, but the wound healing ratio was significantly improved when TGFBR2 was also silenced. Hence, miR-301b-3p enhanced the BC cell invasion and migration and inhibited the TGFBR2 function to suppress BC cell invasion.

### MiR-301b-3p conferred the anti-apoptotic ability to the BC cells

**Figure 5:**
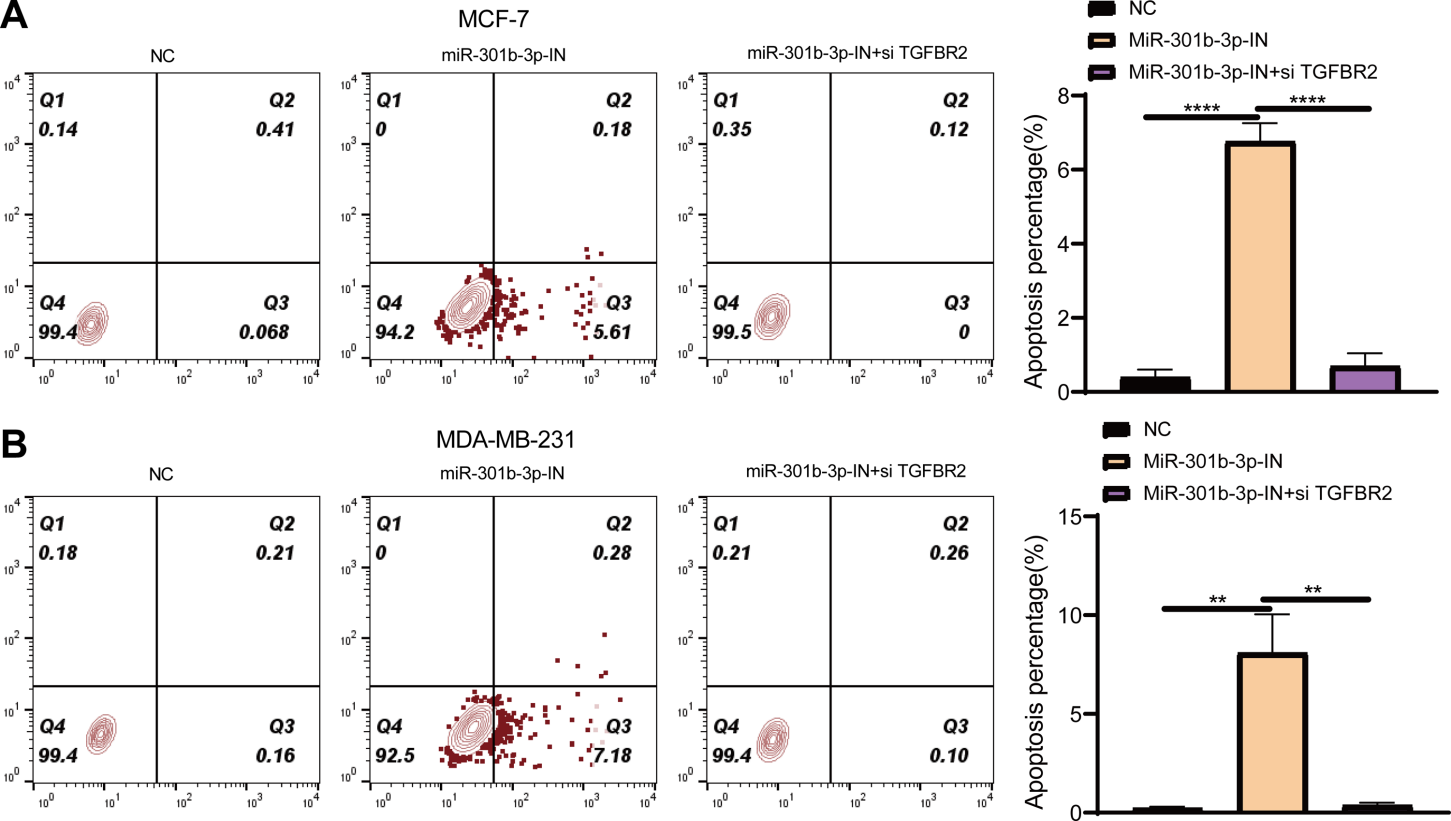
Apoptosis assay in different silenced groups. (A) Flow cytometry for no silencing, miR-301b-3p-silencing and miR-301b-3p+TGFBR2 silencing group in MCF-7 cell lines. (B) Flow cytometry for no silencing, miR-301b-3p-silencing and miR-301b-3p+TGFBR2 silencing group in MDA-MB-231 cell lines. (C) Apoptosis percentage in MCF-7 cell lines. (D) Apoptosis percentage in MDA-MB-231 cell lines. Data were shown as mean ±  SD and ^∗^ ≤ 0.05, ^∗∗^ ≤ 0.01, ^∗∗∗^ ≤ 0.001, ^∗∗∗∗^ ≤ 0.0001. In the four quadrants, Q1, 2, 3, and 4 refers to dead cells, late apoptotic cells, early apoptotic cells, and normal living cells, respectively.

Further, we investigated the role of miR-301b-3p and TGFBR2 genes in anti-apoptotic process of BC cells by flow cytometry. In the MCF-7 cell lines, the living cells in the NC groups accounted for 99.4%, whereas the apoptotic cells increased to 5.61% in the miR-301b-3p-IN group in comparison to 0.068% in the NC groups. When miR-301b-3p and TGFBR2 were silenced at the same time, the BC cells also had no obvious apoptosis (Fig. 5A), while the miR-301b-3p-IN group also showed the highest apoptosis of 68.2% (Fig. 5A). In the MDA-MB-231 cell lines, the BC cells in the NC and miR-301b-3p-IN+si TGFBR2 groups demonstrated a high survival rate. Specifically, the proportion of living cells and the apoptotic cells reached 99.4% and 7.18% in the miR-301b-3p-IN group, respectively, (Fig. 5B), and the highest apoptosis rate of miR-301b-3p-IN group was 77.3% (Fig. 5B). Silencing miR-301b-3p resulted in the highest apoptosis rate indicated the miR-301b-3p conferred a potent anti-apoptotic ability to the BC cells.

### MiR-301b-3p activated the JAK-STAT pathway

**Figure 6:**
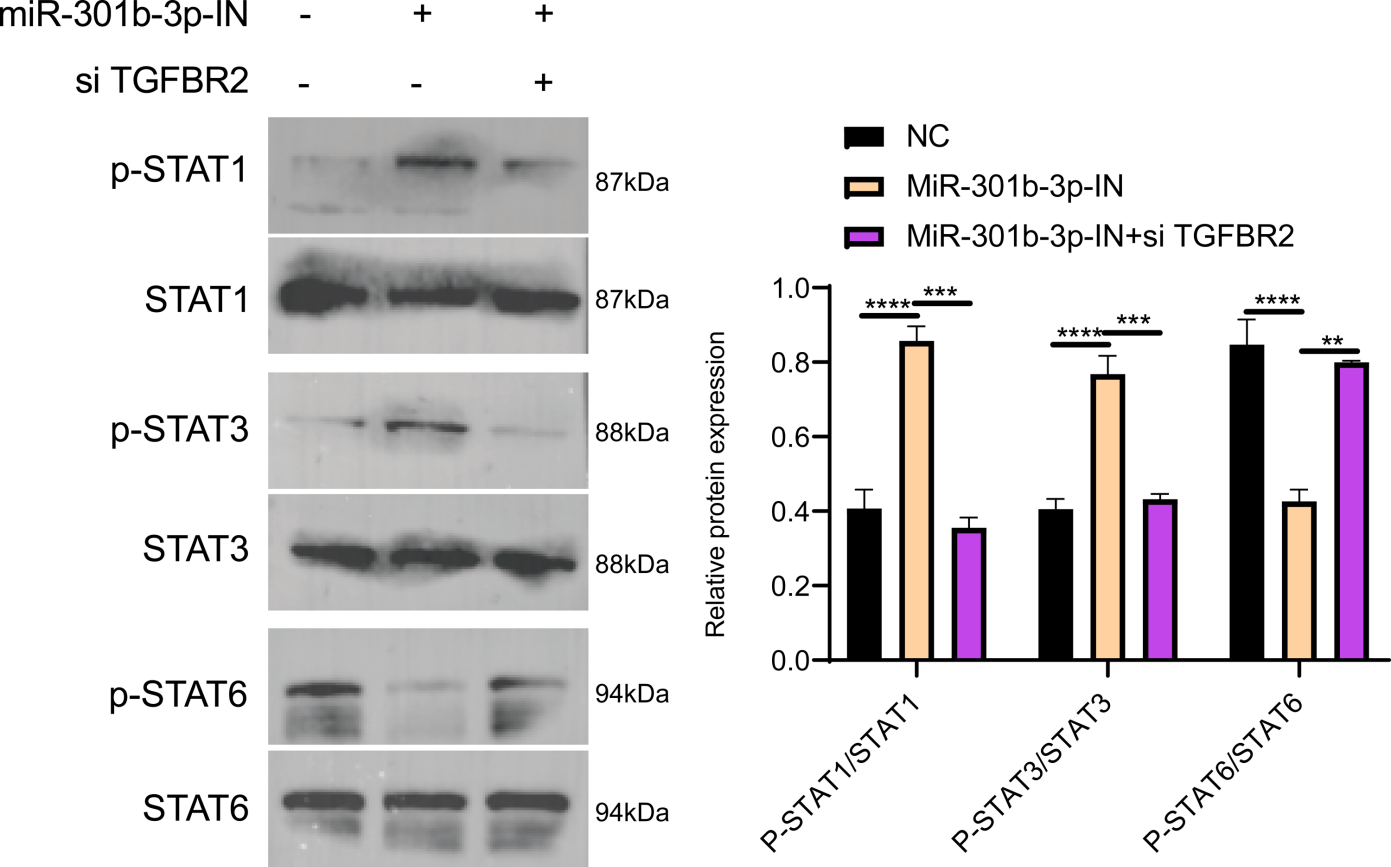
Western blot in different silenced groups. (A) The western blot of STAT1 , STAT3, STAT6 protein and their phosphorylated protein for no silencing, miR-301b-3p-silencing and miR-301b-3p+TGFBR2 silencing group in MCF-7 cell lines. (B) The total protein content detection of STAT1, STAT3, STAT6 protein and their phosphorylated protein in MCF-7 cell lines. Data were shown as mean ± SD and ^∗^ ≤ 0.05, ^∗∗^ ≤ 0.01, ^∗∗∗^ ≤ 0.001, ^∗∗∗∗^ ≤ 0.0001.

The Janus kinases (JAKs) are upstream activators that are responsible for the phosphorylation of STAT (Xin et al., 2020). STAT proteins, especially STAT3 and STAT6, play critical roles in immune-suppression and tumor invasion (Li et al., 2023). In the MCF-7 cell lines, Western blot showed no obvious difference in the effects of TGFBR2 and miR-301b-3p on silencing protein expression levels, but the phosphorylation level of these protein was distinctly different. In the NC group (miR-301b-3p-IN (-)/si TGFBR2 (-)), the phosphorylation level of the STAT6 was remarkably increased, while that of the STAT1 and STAT3 were noticeably increased in the miR-301b-3p silencing group (miR-301b-3p-IN (+)/si TGFBR2 (-)). In the miR-301b-3p and TGFBR2 silencing group (miR-301b-3p-IN (+)/si TGFBR2 (+)), the phosphorylation of STAT3 and STAT6 was greatly promoted (Fig. 6A). Based on the protein expression, silencing miR-301b-3p can noticeably upregulate STAT1 and STAT3 and downregulate STAT6, but when TGFBR2 was also silenced, downregulated expression of STAT6 was elevated (Fig. 6B). These results indicated that miR-301b-3p stimulated the phosphorylation of STAT6 and TGFBR2 could promote the phosphorylation of STAT1 and STAT3.

## Discussion

BC poses serious health threat to the patients (Shang & Xu, 2022). MicroRNAs are a class of post-transcription regulators in organisms (Diener, Keller & Meese, 2022), and a single miRNA can bind to multiple targets and regulate their functions through a variety of mechanisms (Fuchs Wightman et al., 2018; Melo & Esteller, 2011). Recently, several studies reported that miR-301b-3p targets different ligands to mediate BC proliferation, migration and invasion (Fan et al., 2021; Lu et al., 2021; Liu et al., 2021), and that TGFBR2 could both inhibit and promote the growth of BC cells. Therefore, we probed into the relationship of TGFBR2 and miR-301b-3p in BC cell lines, and explored their effects on BC cell apoptosis, viability, and invasion. Our results showed that miR-301b-3p promoted BC progression, while TGFBR2 had a tumor-suppressive function. Importantly, the expression TGFBR2 was inhibited in the BC cells, while suppressing miR-301b-3p restored its expression, suggesting that miR-301b-3p targeted the inhibition of TGFBR2 expression to promote BC development.

Dysfunctional miRNAs often result in an unbalance between oncogenes and anti-oncogenes, increasing the risk of tumorigenesis (Xie et al., 2014). Many studies reported the function of miR-301b-3p in cancer development. MiR-301b-3p suppresses the expression of salt-inducible kinase 1 (SIK1) and regulates the cholesterol and triglyceride homeostasis (Wagschal et al., 2015). Hypoxia-induced miR-301b-3p expression promotes prostate cancer progression through targeting LRP1B (Zheng & Bai, 2019). In liver cancer, tumor-associated neutrophils (TANs) stimulate miR-301b-3p to express, which subsequently inhibits the level of CYLD and LSAMP and upregulates chemokine (CXCL5), leading to a higher infiltration of TANs (Zhou et al., 2019). In bladder cancer, miR-301b-3p downregulates USP13 to further lower the level of tumor suppressor PTEN, contributing to bladder cancer occurrence (Man et al., 2019). These findings demonstrated that miR-301b-3p may fulfill its oncogenic function through diverse mechanisms. The role of canonical TGF-β signaling pathway in various biological processes has been well analyzed (Moustakas & Heldin, 2009). The network members of canonical TGF-β signaling pathway include a TGF-β1 ligand, downstream signal transduction factors (SMAD2, SMAD3, and SMAD4) and two receptors (TGFBR1 and TGFBR2), which are involved in transporting extracellular TGF-β signals into nucleus and regulating the expressions of target genes (Du et al., 2018). Meanwhile, these signaling pathways are regulated by multiple mechanisms including DNA methylation, ubiquitination, deubiquitylation, glycosylation, translational (Wang et al., 2021) and posttranslational regulation (Zhang et al., 2021). TGFBR2 is deubiquitinated by USP11 to maintain the TGF-β signal pathway (Jacko et al., 2016) and the glycosylation of TGFBR2 enhances the radio-resistance and BC cell stemness (Sun et al., 2021); therefore, maintaining a normal TGF-β pathway is essential for cancer cell control. Clinically, low-expressed TGFBR2 in lung cancer tissues is related to poorer prognosis, particularly at an early cancer stage (Lo Sardo et al., 2021). In our study, the level of TGFBR2 as the target gene of miR-301b-3p in BC cells was inhibited, however, the expression of miR-301b-3p was slightly elevated when TGFBR2 and miR-301b-3p were inhibited, which indicated that TGFBR2 was also involved in suppressing miR-301b-3p. Furthermore, the cell viability, invasion and migration, anti-apoptosis ability of BC cells were impaired after silencing miR-301b-3p; however, these oncogenic properties were restored when TGFBR2 also was inhibited. Thus, we hypothesized that the (PI3K)/AKT signaling pathway or other cancer-promoting pathways may be activated during the processes. STAT proteins are a class of crucial overexpressed signal transducers in tumor cells (Li et al., 2023). In normal physiology, PIAS, SOCS and PTPs regulate the activation of STAT (Croker, Kiu & Nicholson, 2008; Sivaganesh et al., 2021; Taheri, Oskooei & Ghafouri-Fard, 2019). However, STAT3 and STAT6 are often over-activated in tumor cells to induce tumor invasion and immune suppression (Li et al., 2023; Yu et al., 2014; Inghirami et al., 2005). In our study, the STAT3 phosphorylation was inhibited by miR-301b-3p, which might be caused by an underlying crosstalk mechanism, however, the specific details required further analysis. MiR-301b-3p activating the STAT6 phosphorylation also plays an important role in tumorigenesis. STAT1 has been reported to have both cancer-promoting and cancer-suppressing properties (Chou et al., 2022; Wang et al., 2020). Our results indicated that the phosphorylation of STAT1 activated by TGFBR2 fulfilled a cancer-suppressing function in BC. This finding further supports the function of TGFBR2 as a breast cancer oncogenic factor and suggests that it inhibits BC progression by modulating JAK-STAT signaling.

In our study, we found the carcinogenesis effect of miR-301b-3p and the tumor-suppressive effect of TGFBR2 on BC cells. However, there were still some limitations in this study. Firstly, further experiments are needed to confirm the antagonistic correlation between TGFBR2 and miR-301b-3p. Secondly, the potential role of miR-301b-3p in inhibiting the phosphorylation of STAT3 during BC development requires verification. Finally, the mechanism of TGFBR2 in regulating the phosphorylation of STAT1 to suppress BC cancer development should be validated.

## Conclusion

The present research explored the cancer-promoting effect of miR-301b-3p on BC. High-expressed miR-301b-3p functioned critically in promoting BC development, including in cell growth, proliferation and anti-apoptosis. TGFBR2 had a tumor-suppressive effect but its expression was suppressed by miR-301b-3p in BC cells.

## Supplemental Information

10.7717/peerj.18324/supp-1 Supplemental Information 1 Experimental raw data

10.7717/peerj.18324/supp-2 Supplemental Information 2 MIQE checklist

10.7717/peerj.18324/supp-3 Supplemental Information 3 STAT1 raw data

10.7717/peerj.18324/supp-4 Supplemental Information 4 STAT3 raw data

10.7717/peerj.18324/supp-5 Supplemental Information 5 STAT6 raw data

10.7717/peerj.18324/supp-6 Supplemental Information 6 WB raw data

## Abbreviations

LUAD: Lung adenocarcinoma
ssGSEA: Single-sample gene set enrichment analysis
PPI: protein-protein interaction
DEGs: differentially expressed genes
LASSO: Least Absolute Shrinkage and Selection Operator Regression
TCGA: The Cancer Genome Atlas
ROC: receiver operating characteristic
AUC: area under the curve
DCA: Decision curve analysis
OS: Overall Survival
NSCLC: non–small-cell lung cancer
TME: tumor microenvironment
GEO: Gene Expression Omnibus
KEGG: Kyoto Encyclopedia of Genes and Genomes
GO: gene ontology
IC50: inhibitory concentration
TIDE: Tumor immune dysfunction and exclusion
KM: Kaplan–Meier
PCA: principal component analysis
CR: Complete Response,
PR: Partial Response
SD: Stable Disease
PD: Progressive Disease

## Additional Information and Declarations

### Data Availability

The following information was supplied regarding data availability: The raw data is available at GitHub and Zenodo: – https://github.com/1yxz/My-Updated-Data.git. – 1yxz. (2024). 1yxz/My-Updated-Data: Raw data (v.1.1.2). Zenodo. https://doi.org/10.5281/zenodo.13358741. The experiment data is available in the Supplementary Files.
