# Supplementary figures and images for: MiR-301b-3p promotes breast cancer development through inhibiting the expression of transforming growth factor-beta receptor 2 (part 1 of 2)
Source: PeerJ. 2024 Nov 5;12:e18324. doi: 10.7717/peerj.18324 (PMC11546148; doi:10.7717/peerj.18324)

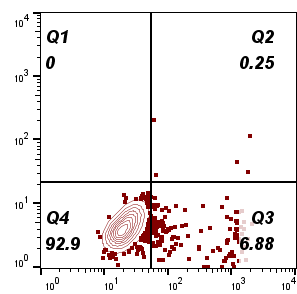

Supplement: Supplemental Information 1 [file peerj-12-18324-s001.zip › Apoptosis/MCF-7/mi-1.png]

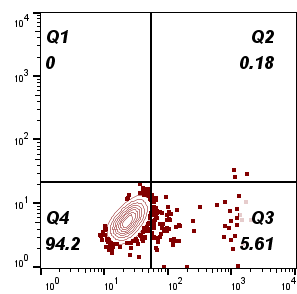

Supplement: Supplemental Information 1 [file peerj-12-18324-s001.zip › Apoptosis/MCF-7/mi-2.png]

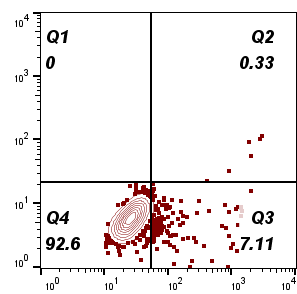

Supplement: Supplemental Information 1 [file peerj-12-18324-s001.zip › Apoptosis/MCF-7/mi-3.png]

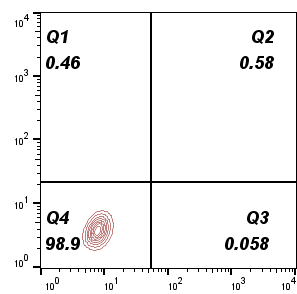

Supplement: Supplemental Information 1 [file peerj-12-18324-s001.zip › Apoptosis/MCF-7/mi-si-1.png]

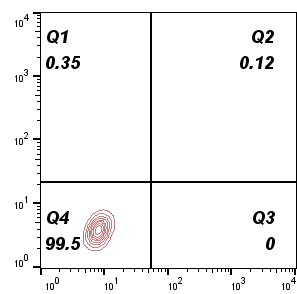

Supplement: Supplemental Information 1 [file peerj-12-18324-s001.zip › Apoptosis/MCF-7/mi-si-2.png]

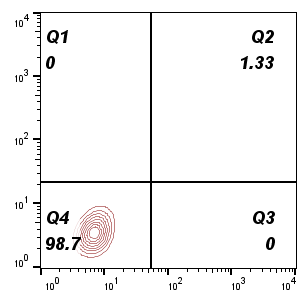

Supplement: Supplemental Information 1 [file peerj-12-18324-s001.zip › Apoptosis/MCF-7/mi-si-3.png]

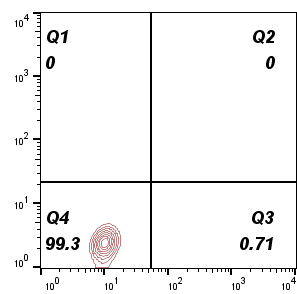

Supplement: Supplemental Information 1 [file peerj-12-18324-s001.zip › Apoptosis/MCF-7/NC-1.png]

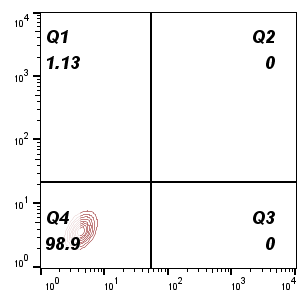

Supplement: Supplemental Information 1 [file peerj-12-18324-s001.zip › Apoptosis/MCF-7/NC-2.png]

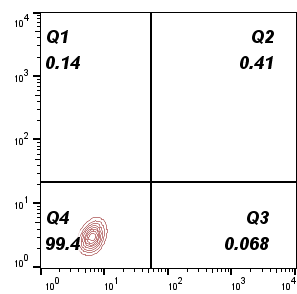

Supplement: Supplemental Information 1 [file peerj-12-18324-s001.zip › Apoptosis/MCF-7/NC-3.png]

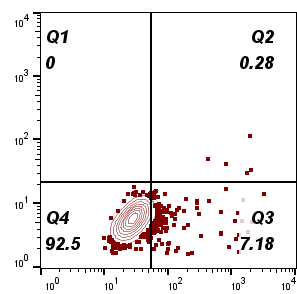

Supplement: Supplemental Information 1 [file peerj-12-18324-s001.zip › Apoptosis/MDA-MB-231/mi-1.png]

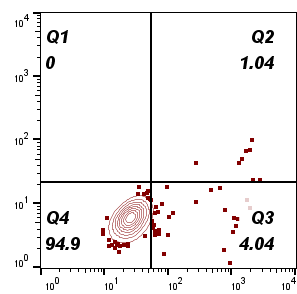

Supplement: Supplemental Information 1 [file peerj-12-18324-s001.zip › Apoptosis/MDA-MB-231/mi-2.png]

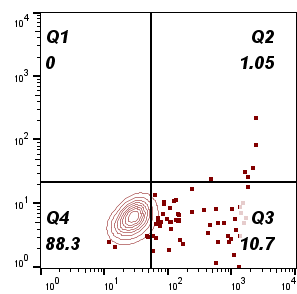

Supplement: Supplemental Information 1 [file peerj-12-18324-s001.zip › Apoptosis/MDA-MB-231/mi-3.png]

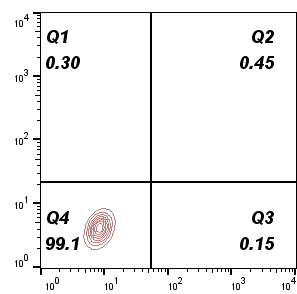

Supplement: Supplemental Information 1 [file peerj-12-18324-s001.zip › Apoptosis/MDA-MB-231/mi-si-1.png]

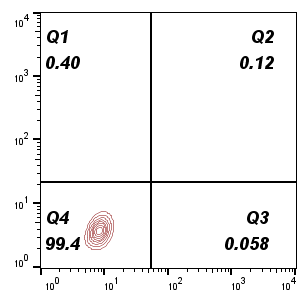

Supplement: Supplemental Information 1 [file peerj-12-18324-s001.zip › Apoptosis/MDA-MB-231/mi-si-2.png]

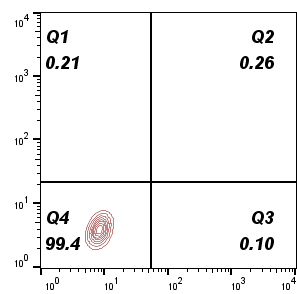

Supplement: Supplemental Information 1 [file peerj-12-18324-s001.zip › Apoptosis/MDA-MB-231/mi-si-3.png]

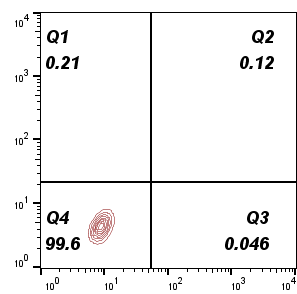

Supplement: Supplemental Information 1 [file peerj-12-18324-s001.zip › Apoptosis/MDA-MB-231/NC-1.png]

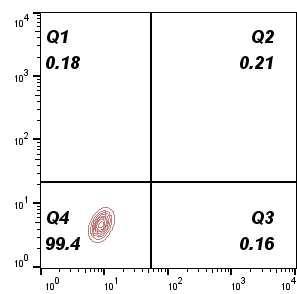

Supplement: Supplemental Information 1 [file peerj-12-18324-s001.zip › Apoptosis/MDA-MB-231/NC-2.png]

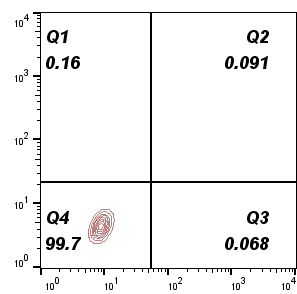

Supplement: Supplemental Information 1 [file peerj-12-18324-s001.zip › Apoptosis/MDA-MB-231/NC-3.png]

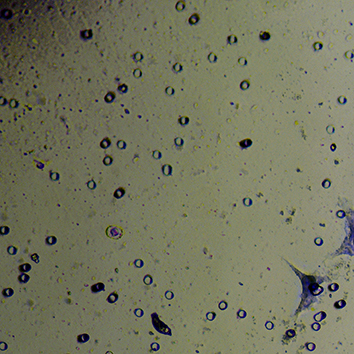

Supplement: Supplemental Information 1 [file peerj-12-18324-s001.zip › transwell/MCF7/invasion/mi-in-1.jpg]

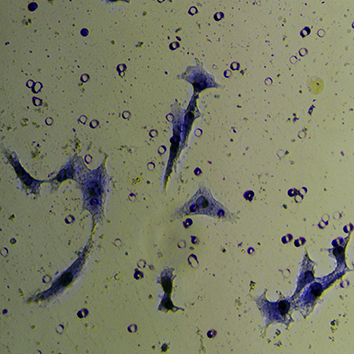

Supplement: Supplemental Information 1 [file peerj-12-18324-s001.zip › transwell/MCF7/invasion/mi-in-2.jpg]

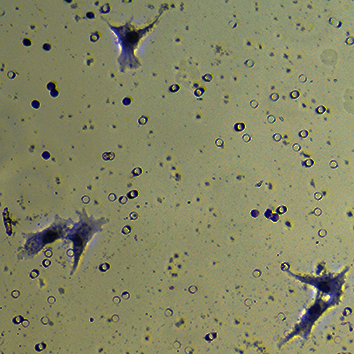

Supplement: Supplemental Information 1 [file peerj-12-18324-s001.zip › transwell/MCF7/invasion/mi-in-3.jpg]

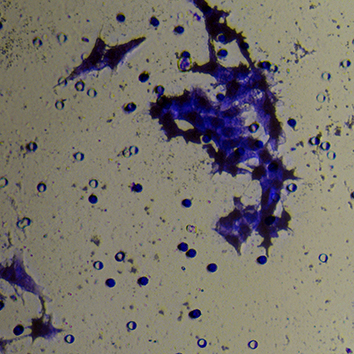

Supplement: Supplemental Information 1 [file peerj-12-18324-s001.zip › transwell/MCF7/invasion/mi-si-1.jpg]

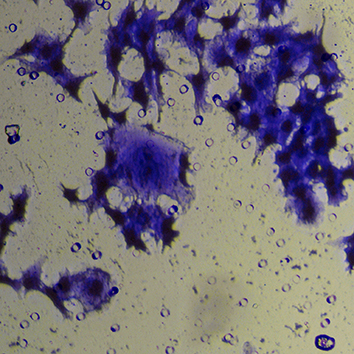

Supplement: Supplemental Information 1 [file peerj-12-18324-s001.zip › transwell/MCF7/invasion/mi-si-2.jpg]

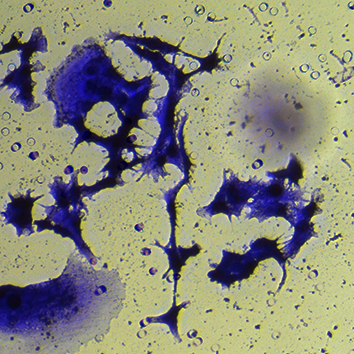

Supplement: Supplemental Information 1 [file peerj-12-18324-s001.zip › transwell/MCF7/invasion/mi-si-3.jpg]

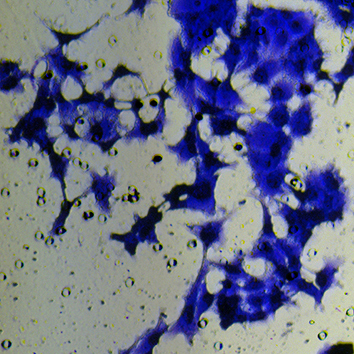

Supplement: Supplemental Information 1 [file peerj-12-18324-s001.zip › transwell/MCF7/invasion/NC-1.jpg]

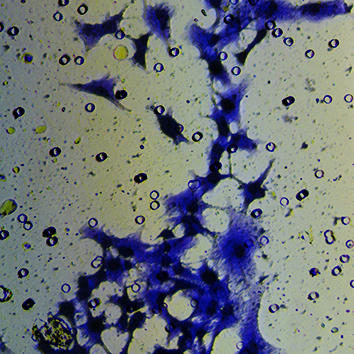

Supplement: Supplemental Information 1 [file peerj-12-18324-s001.zip › transwell/MCF7/invasion/NC-2.jpg]

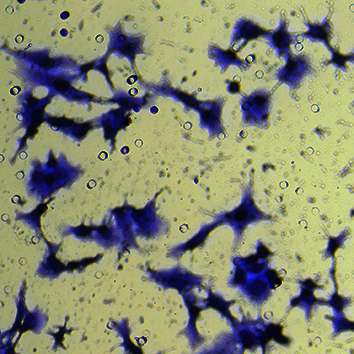

Supplement: Supplemental Information 1 [file peerj-12-18324-s001.zip › transwell/MCF7/invasion/NC-3.jpg]

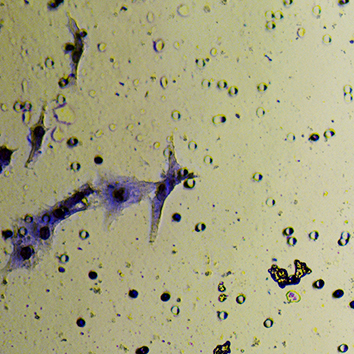

Supplement: Supplemental Information 1 [file peerj-12-18324-s001.zip › transwell/MCF7/migration/mi-1.jpg]

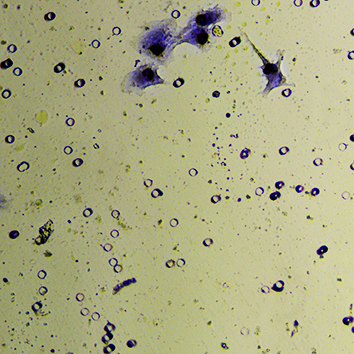

Supplement: Supplemental Information 1 [file peerj-12-18324-s001.zip › transwell/MCF7/migration/mi-2.jpg]

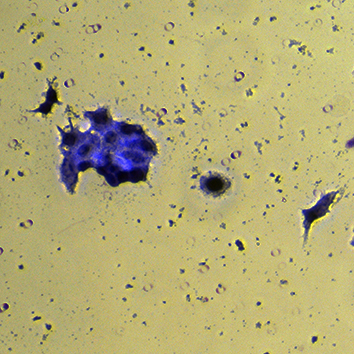

Supplement: Supplemental Information 1 [file peerj-12-18324-s001.zip › transwell/MCF7/migration/mi-3.jpg]

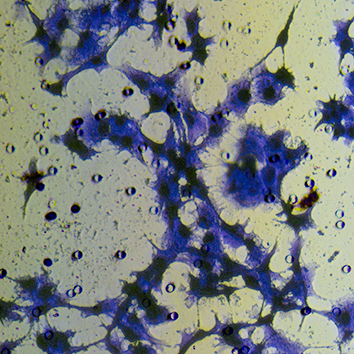

Supplement: Supplemental Information 1 [file peerj-12-18324-s001.zip › transwell/MCF7/migration/mi-si-1.jpg]

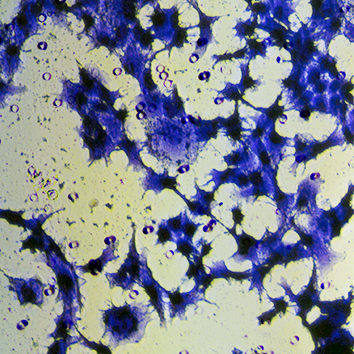

Supplement: Supplemental Information 1 [file peerj-12-18324-s001.zip › transwell/MCF7/migration/mi-si-2.jpg]

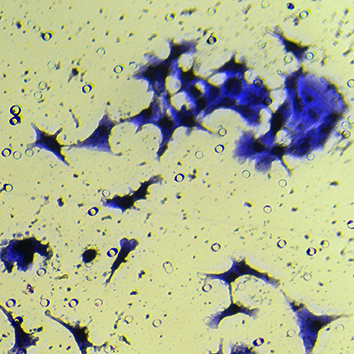

Supplement: Supplemental Information 1 [file peerj-12-18324-s001.zip › transwell/MCF7/migration/mi-si-3.jpg]

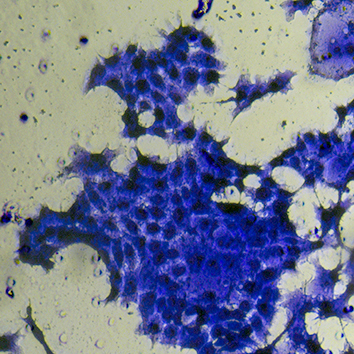

Supplement: Supplemental Information 1 [file peerj-12-18324-s001.zip › transwell/MCF7/migration/NC-1.jpg]

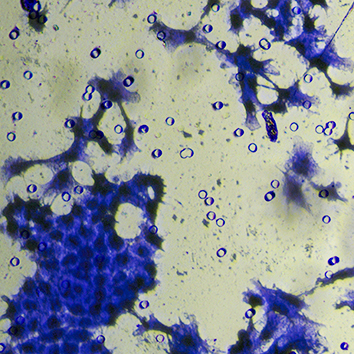

Supplement: Supplemental Information 1 [file peerj-12-18324-s001.zip › transwell/MCF7/migration/NC-2.jpg]

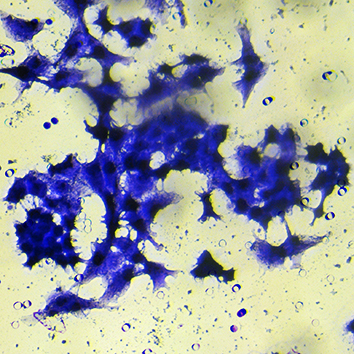

Supplement: Supplemental Information 1 [file peerj-12-18324-s001.zip › transwell/MCF7/migration/NC-3.jpg]

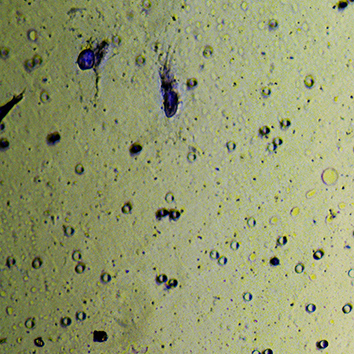

Supplement: Supplemental Information 1 [file peerj-12-18324-s001.zip › transwell/MDA-MB-231/invasion/mi-1.jpg]

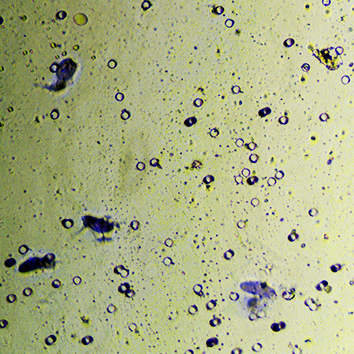

Supplement: Supplemental Information 1 [file peerj-12-18324-s001.zip › transwell/MDA-MB-231/invasion/mi-2.jpg]

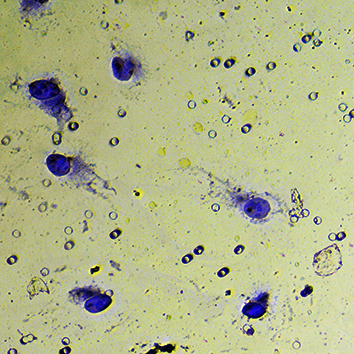

Supplement: Supplemental Information 1 [file peerj-12-18324-s001.zip › transwell/MDA-MB-231/invasion/mi-3.jpg]

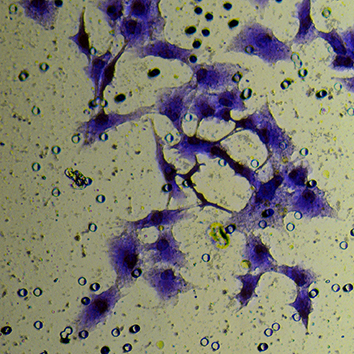

Supplement: Supplemental Information 1 [file peerj-12-18324-s001.zip › transwell/MDA-MB-231/invasion/mi-si-1.jpg]

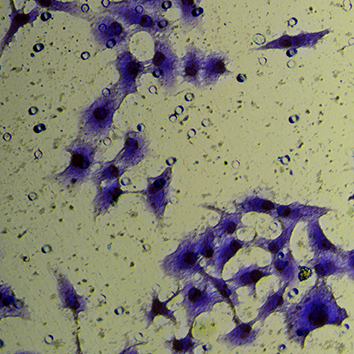

Supplement: Supplemental Information 1 [file peerj-12-18324-s001.zip › transwell/MDA-MB-231/invasion/mi-si-2.jpg]

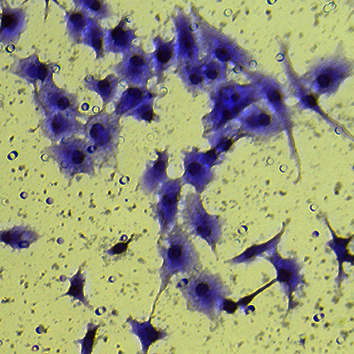

Supplement: Supplemental Information 1 [file peerj-12-18324-s001.zip › transwell/MDA-MB-231/invasion/mi-si-3.jpg]

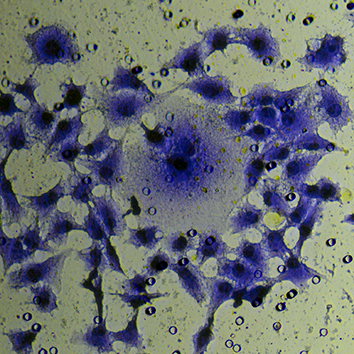

Supplement: Supplemental Information 1 [file peerj-12-18324-s001.zip › transwell/MDA-MB-231/invasion/NC-1.jpg]

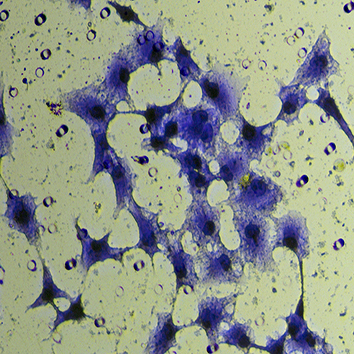

Supplement: Supplemental Information 1 [file peerj-12-18324-s001.zip › transwell/MDA-MB-231/invasion/NC-2.jpg]

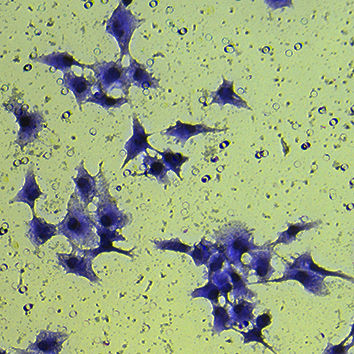

Supplement: Supplemental Information 1 [file peerj-12-18324-s001.zip › transwell/MDA-MB-231/invasion/NC-3.jpg]

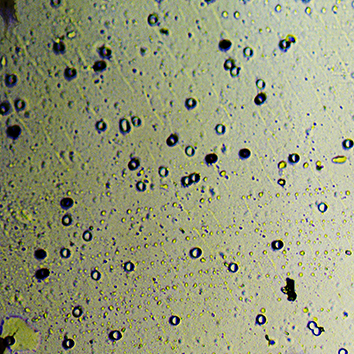

Supplement: Supplemental Information 1 [file peerj-12-18324-s001.zip › transwell/MDA-MB-231/migration/mi-1.jpg]

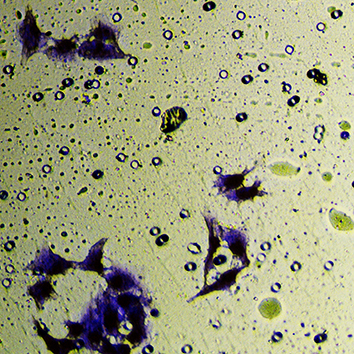

Supplement: Supplemental Information 1 [file peerj-12-18324-s001.zip › transwell/MDA-MB-231/migration/mi-2.jpg]

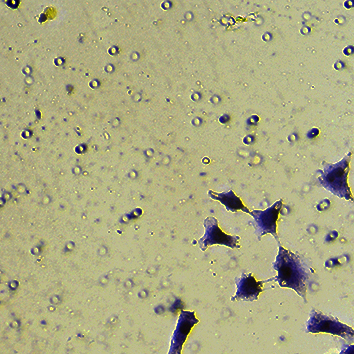

Supplement: Supplemental Information 1 [file peerj-12-18324-s001.zip › transwell/MDA-MB-231/migration/mi-3.jpg]

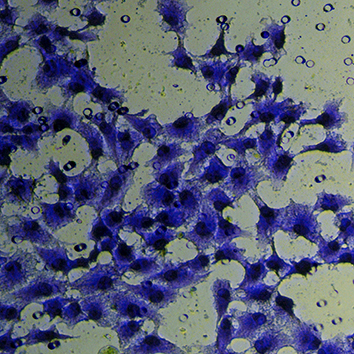

Supplement: Supplemental Information 1 [file peerj-12-18324-s001.zip › transwell/MDA-MB-231/migration/mi-si-1.jpg]

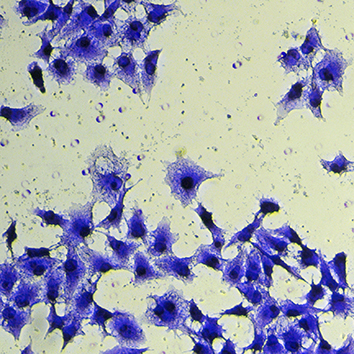

Supplement: Supplemental Information 1 [file peerj-12-18324-s001.zip › transwell/MDA-MB-231/migration/mi-si-2.jpg]

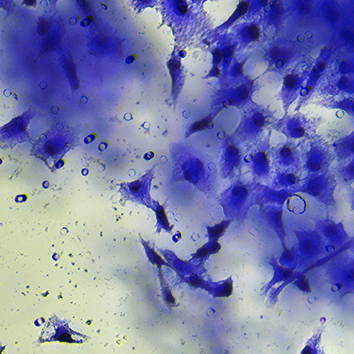

Supplement: Supplemental Information 1 [file peerj-12-18324-s001.zip › transwell/MDA-MB-231/migration/mi-si-3.jpg]

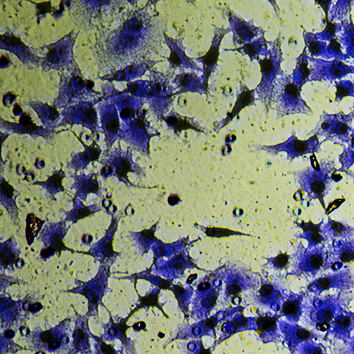

Supplement: Supplemental Information 1 [file peerj-12-18324-s001.zip › transwell/MDA-MB-231/migration/NC-1.jpg]

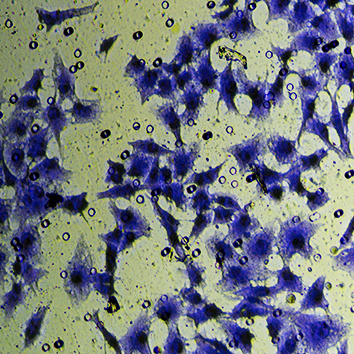

Supplement: Supplemental Information 1 [file peerj-12-18324-s001.zip › transwell/MDA-MB-231/migration/NC-2.jpg]

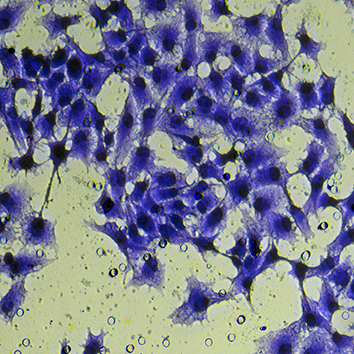

Supplement: Supplemental Information 1 [file peerj-12-18324-s001.zip › transwell/MDA-MB-231/migration/NC-3.jpg]

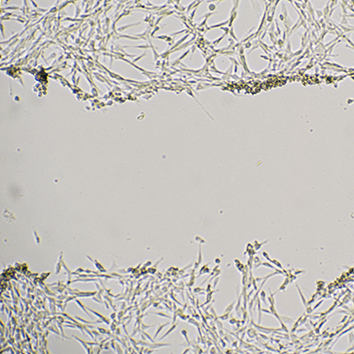

Supplement: Supplemental Information 1 [file peerj-12-18324-s001.zip › wound healing/MCF-7/0h/mi (1).tif]

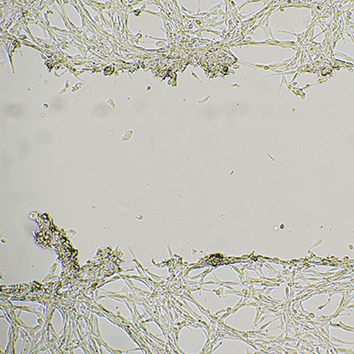

Supplement: Supplemental Information 1 [file peerj-12-18324-s001.zip › wound healing/MCF-7/0h/mi (2).tif]

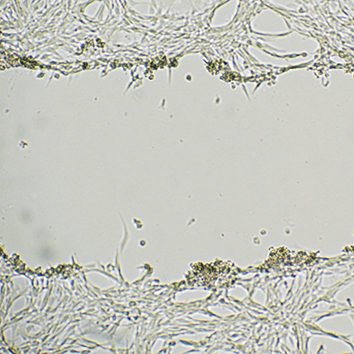

Supplement: Supplemental Information 1 [file peerj-12-18324-s001.zip › wound healing/MCF-7/0h/mi (3).tif]

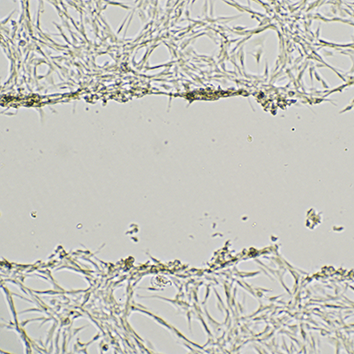

Supplement: Supplemental Information 1 [file peerj-12-18324-s001.zip › wound healing/MCF-7/0h/mi-si (1).tif]

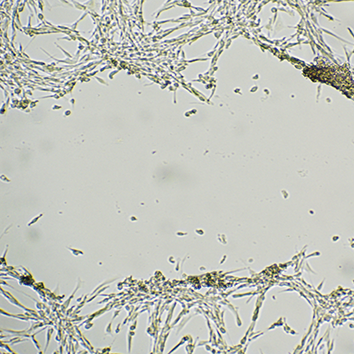

Supplement: Supplemental Information 1 [file peerj-12-18324-s001.zip › wound healing/MCF-7/0h/mi-si (2).tif]

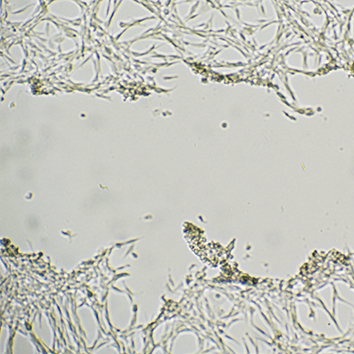

Supplement: Supplemental Information 1 [file peerj-12-18324-s001.zip › wound healing/MCF-7/0h/mi-si (3).tif]

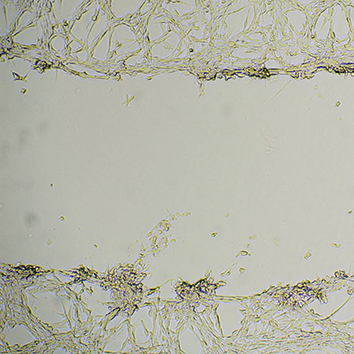

Supplement: Supplemental Information 1 [file peerj-12-18324-s001.zip › wound healing/MCF-7/0h/NC (1).tif]

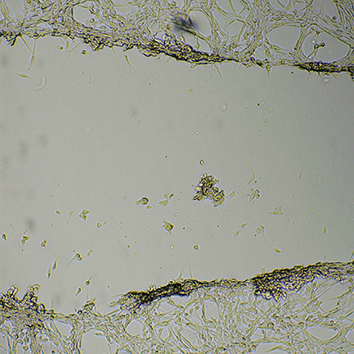

Supplement: Supplemental Information 1 [file peerj-12-18324-s001.zip › wound healing/MCF-7/0h/NC (2).tif]

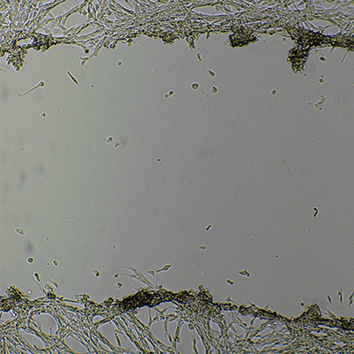

Supplement: Supplemental Information 1 [file peerj-12-18324-s001.zip › wound healing/MCF-7/0h/NC (3).tif]

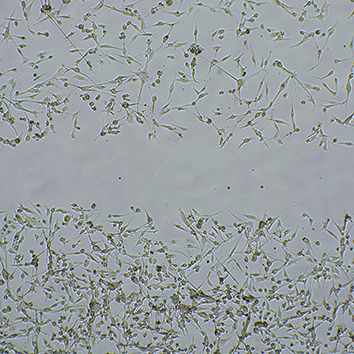

Supplement: Supplemental Information 1 [file peerj-12-18324-s001.zip › wound healing/MCF-7/24h/mi-1.tif]

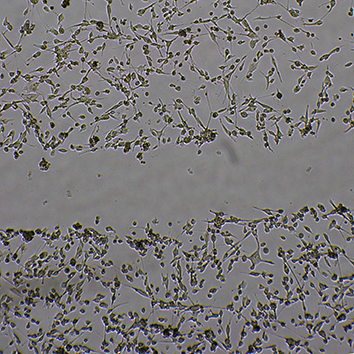

Supplement: Supplemental Information 1 [file peerj-12-18324-s001.zip › wound healing/MCF-7/24h/mi-2.tif]

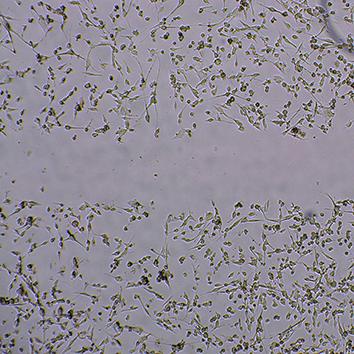

Supplement: Supplemental Information 1 [file peerj-12-18324-s001.zip › wound healing/MCF-7/24h/mi-3.tif]

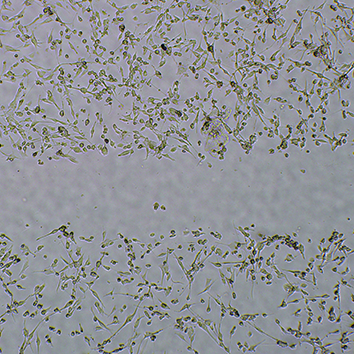

Supplement: Supplemental Information 1 [file peerj-12-18324-s001.zip › wound healing/MCF-7/24h/mi-si-1.tif]

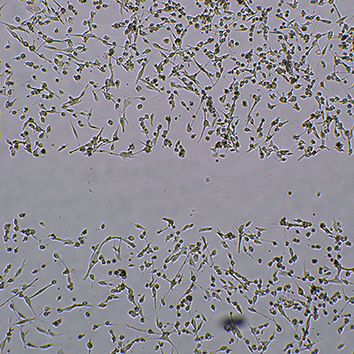

Supplement: Supplemental Information 1 [file peerj-12-18324-s001.zip › wound healing/MCF-7/24h/mi-si-2.tif]

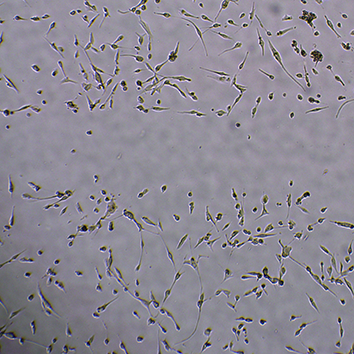

Supplement: Supplemental Information 1 [file peerj-12-18324-s001.zip › wound healing/MCF-7/24h/mi-si-3.tif]

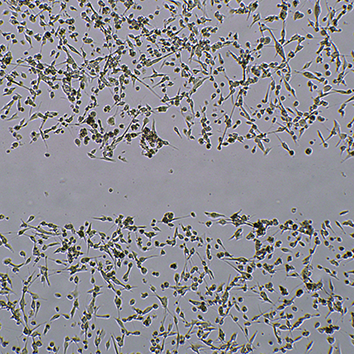

Supplement: Supplemental Information 1 [file peerj-12-18324-s001.zip › wound healing/MCF-7/24h/NC-1.tif]

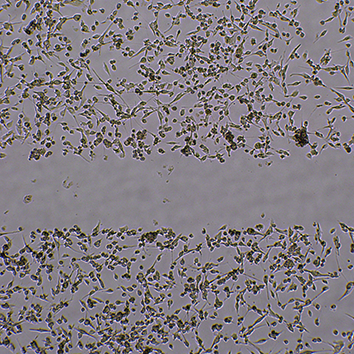

Supplement: Supplemental Information 1 [file peerj-12-18324-s001.zip › wound healing/MCF-7/24h/NC-2.tif]

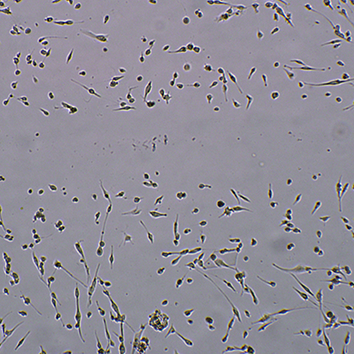

Supplement: Supplemental Information 1 [file peerj-12-18324-s001.zip › wound healing/MCF-7/24h/NC-3.tif]

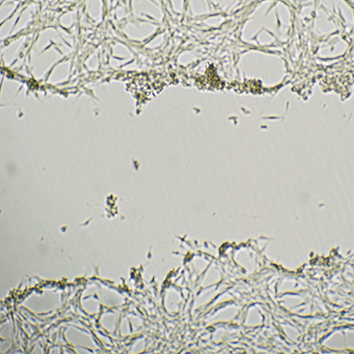

Supplement: Supplemental Information 1 [file peerj-12-18324-s001.zip › wound healing/MDA-MB-231/0h/mi-1.tif]

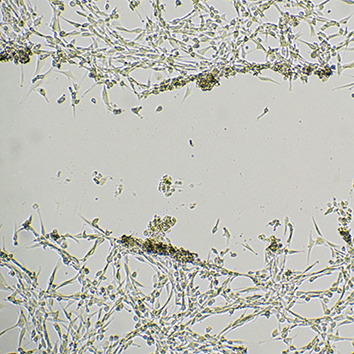

Supplement: Supplemental Information 1 [file peerj-12-18324-s001.zip › wound healing/MDA-MB-231/0h/mi-2.tif]

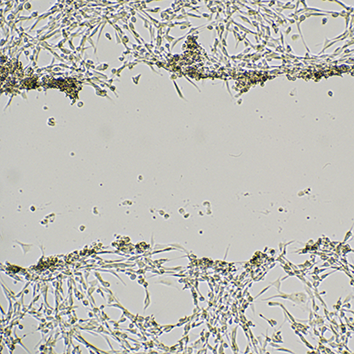

Supplement: Supplemental Information 1 [file peerj-12-18324-s001.zip › wound healing/MDA-MB-231/0h/mi-3.tif]

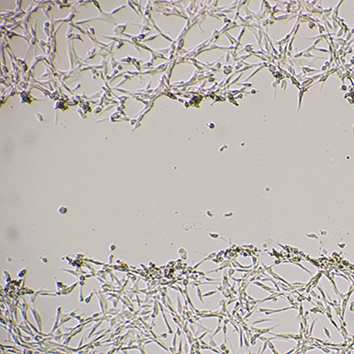

Supplement: Supplemental Information 1 [file peerj-12-18324-s001.zip › wound healing/MDA-MB-231/0h/mi-si-1.tif]

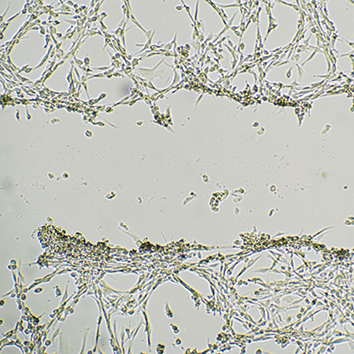

Supplement: Supplemental Information 1 [file peerj-12-18324-s001.zip › wound healing/MDA-MB-231/0h/mi-si-2.tif]

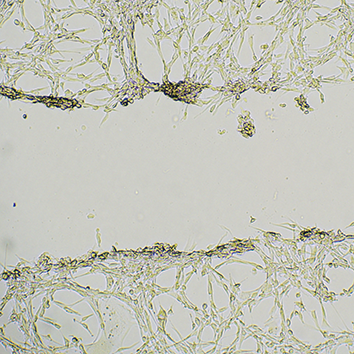

Supplement: Supplemental Information 1 [file peerj-12-18324-s001.zip › wound healing/MDA-MB-231/0h/mi-si-3.tif]

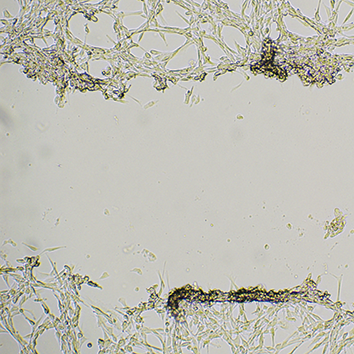

Supplement: Supplemental Information 1 [file peerj-12-18324-s001.zip › wound healing/MDA-MB-231/0h/NC-1.tif]

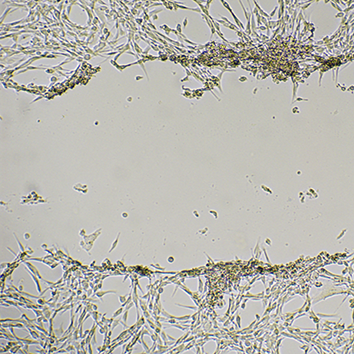

Supplement: Supplemental Information 1 [file peerj-12-18324-s001.zip › wound healing/MDA-MB-231/0h/NC-2.tif]

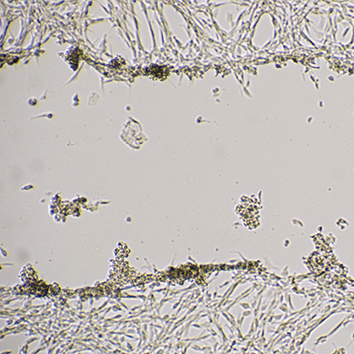

Supplement: Supplemental Information 1 [file peerj-12-18324-s001.zip › wound healing/MDA-MB-231/0h/NC-3.tif]

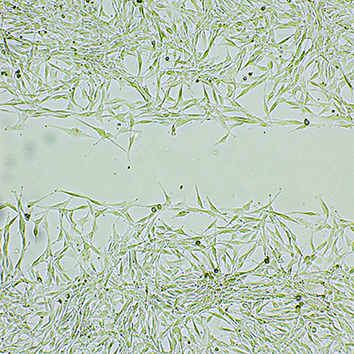

Supplement: Supplemental Information 1 [file peerj-12-18324-s001.zip › wound healing/MDA-MB-231/24h/mi-1.tif]

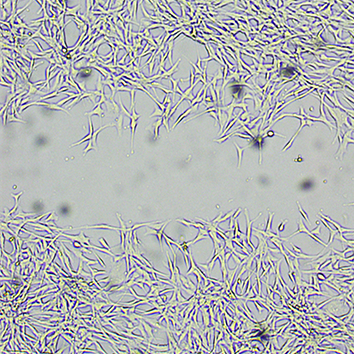

Supplement: Supplemental Information 1 [file peerj-12-18324-s001.zip › wound healing/MDA-MB-231/24h/mi-2.tif]

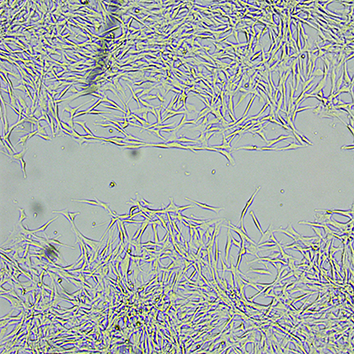

Supplement: Supplemental Information 1 [file peerj-12-18324-s001.zip › wound healing/MDA-MB-231/24h/mi-3.tif]

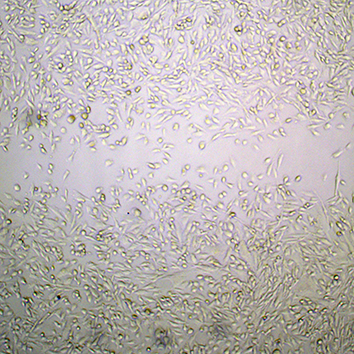

Supplement: Supplemental Information 1 [file peerj-12-18324-s001.zip › wound healing/MDA-MB-231/24h/mi-si-1.tif]

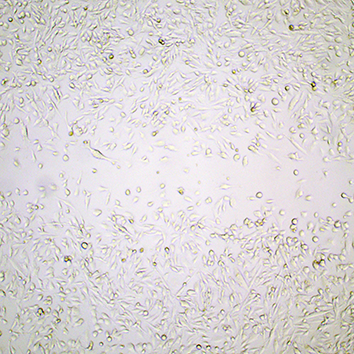

Supplement: Supplemental Information 1 [file peerj-12-18324-s001.zip › wound healing/MDA-MB-231/24h/mi-si-2.tif]

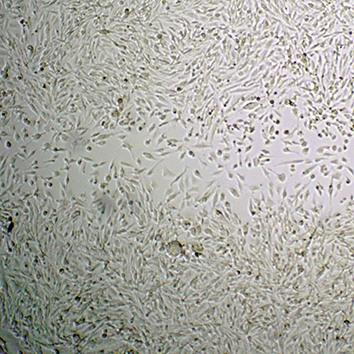

Supplement: Supplemental Information 1 [file peerj-12-18324-s001.zip › wound healing/MDA-MB-231/24h/mi-si-3.tif]

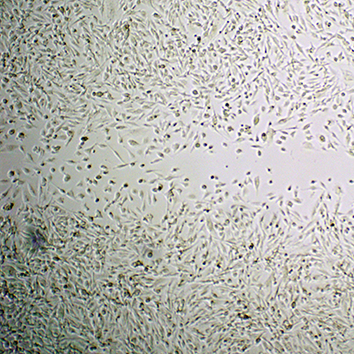

Supplement: Supplemental Information 1 [file peerj-12-18324-s001.zip › wound healing/MDA-MB-231/24h/NC-1.tif]

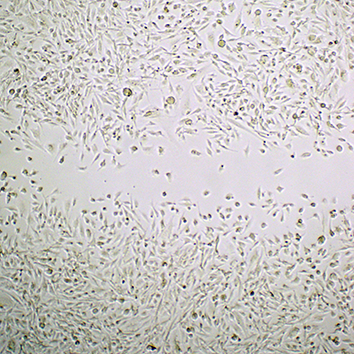

Supplement: Supplemental Information 1 [file peerj-12-18324-s001.zip › wound healing/MDA-MB-231/24h/NC-2.tif]

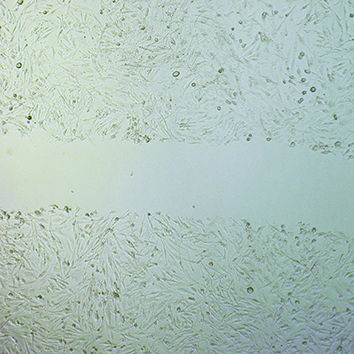

Supplement: Supplemental Information 1 [file peerj-12-18324-s001.zip › wound healing/MDA-MB-231/24h/NC-3.tif]

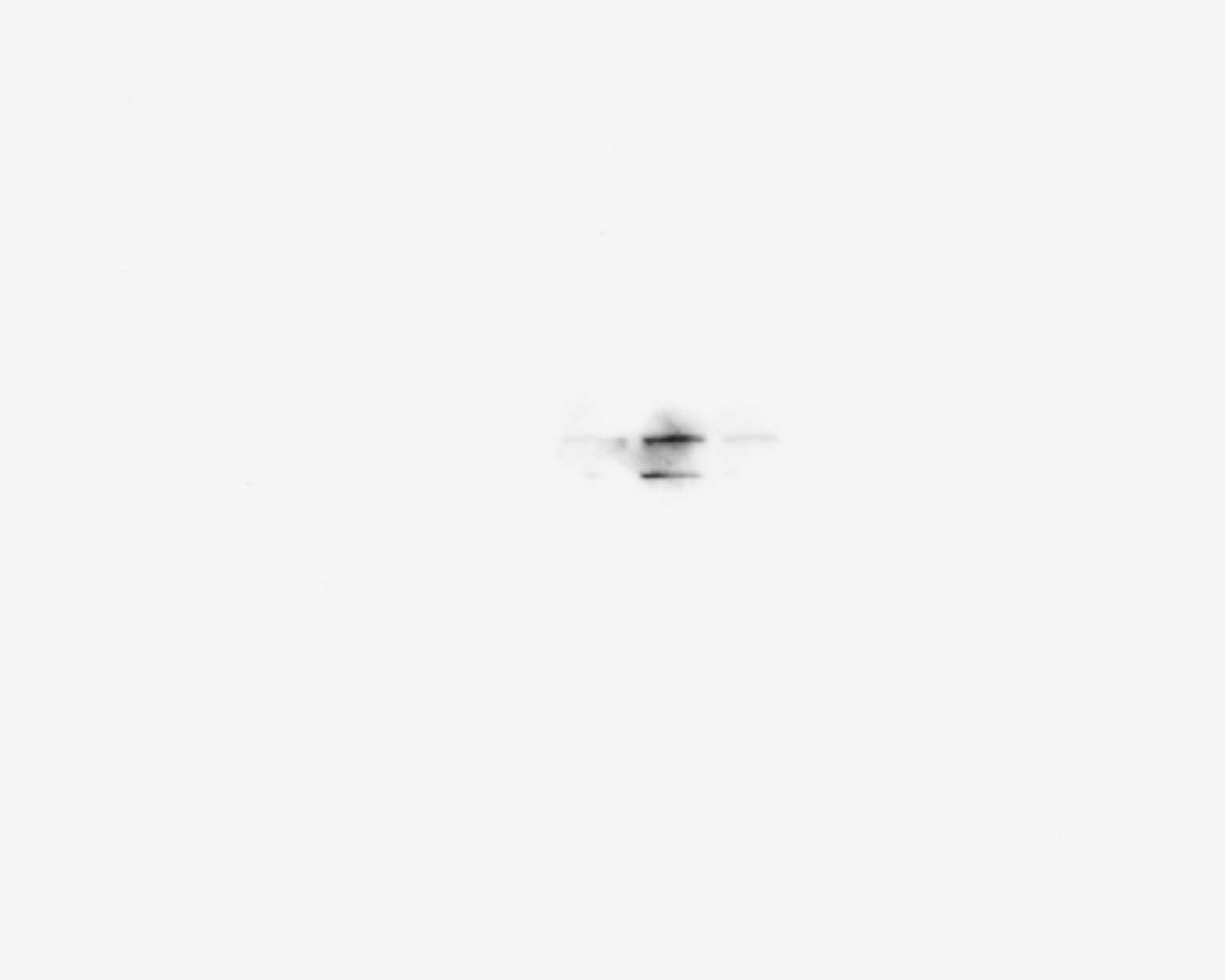

Supplement: Supplemental Information 3 [file peerj-12-18324-s003.zip › pstat1+stat1 1_1(Chemiluminescence).tif]

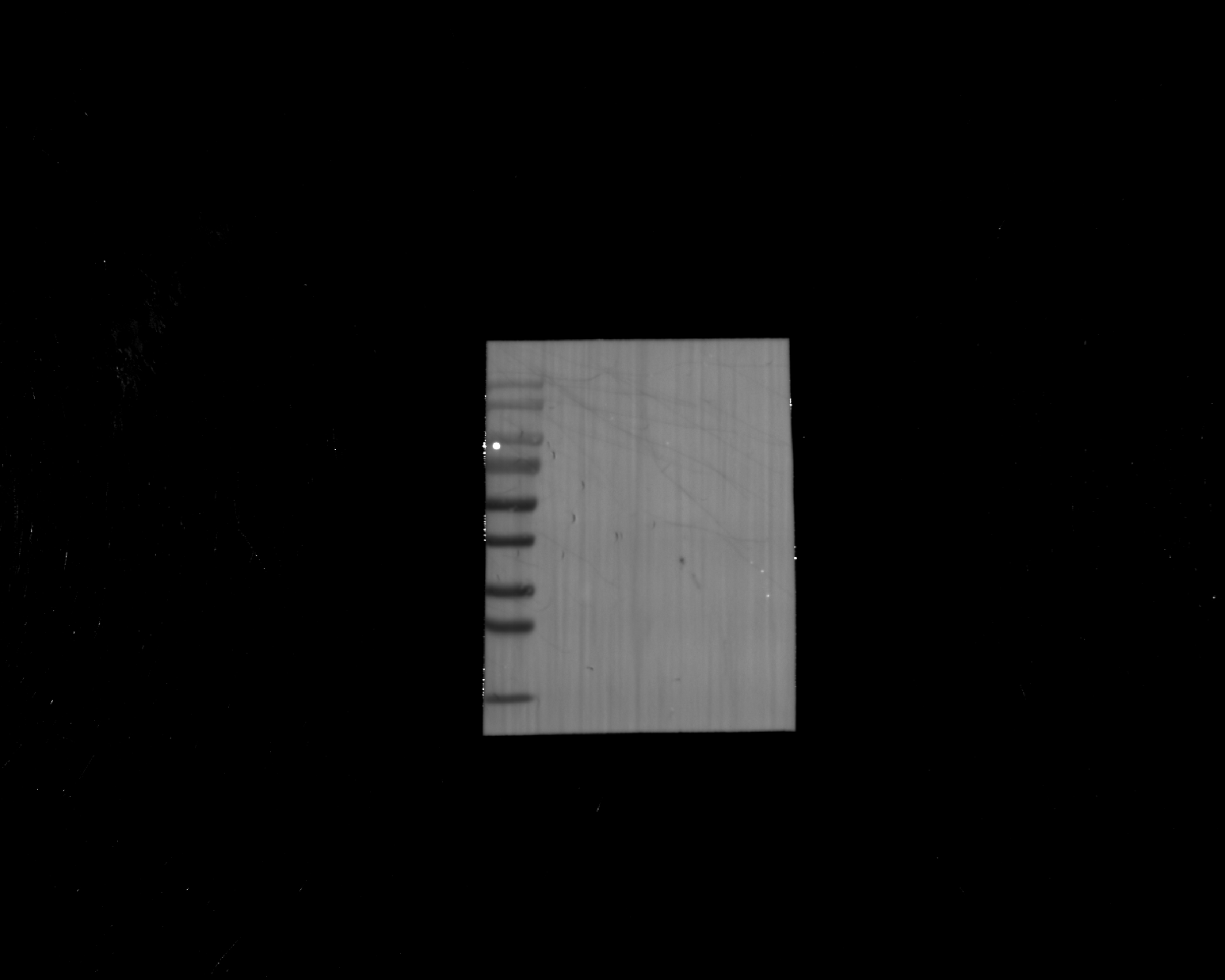

Supplement: Supplemental Information 3 [file peerj-12-18324-s003.zip › pstat1+stat1 1_1(Colorimetric).tif]

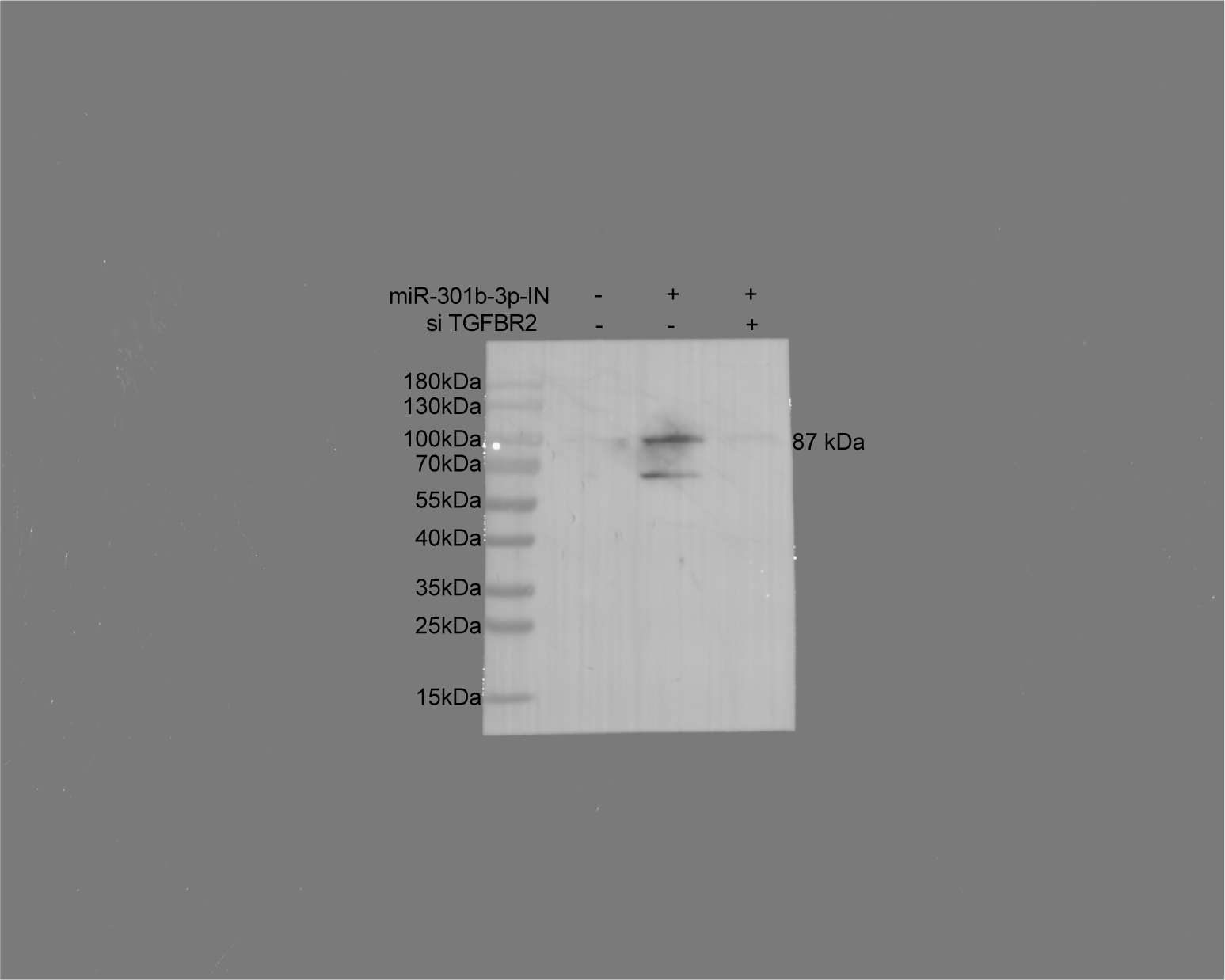

Supplement: Supplemental Information 3 [file peerj-12-18324-s003.zip › pstat1+stat1 1_1(Composite)-01.tif]

|                |   |   |   |
|----------------|---|---|---|
| miR-301b-3p-IN | - | + | + |
| si TGFB2       | - | - | + |

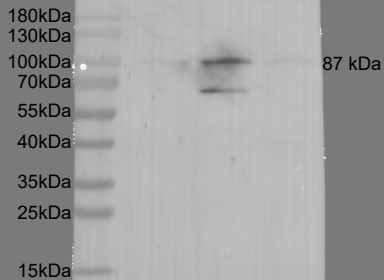

Supplement: Supplemental Information 3 [file peerj-12-18324-s003.zip › pstat1+stat1 1_1(Composite).pdf]

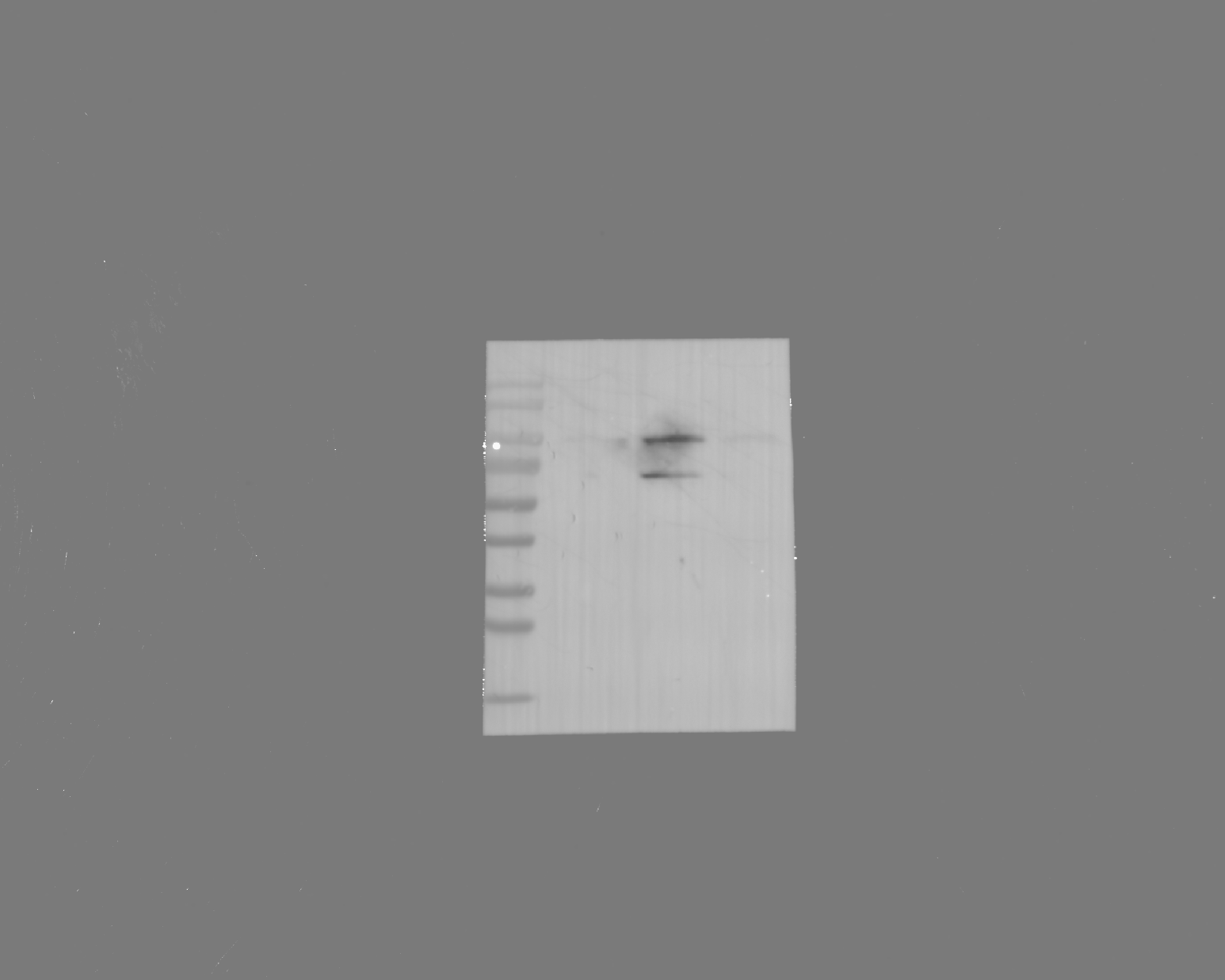

Supplement: Supplemental Information 3 [file peerj-12-18324-s003.zip › pstat1+stat1 1_1(Composite).tif]

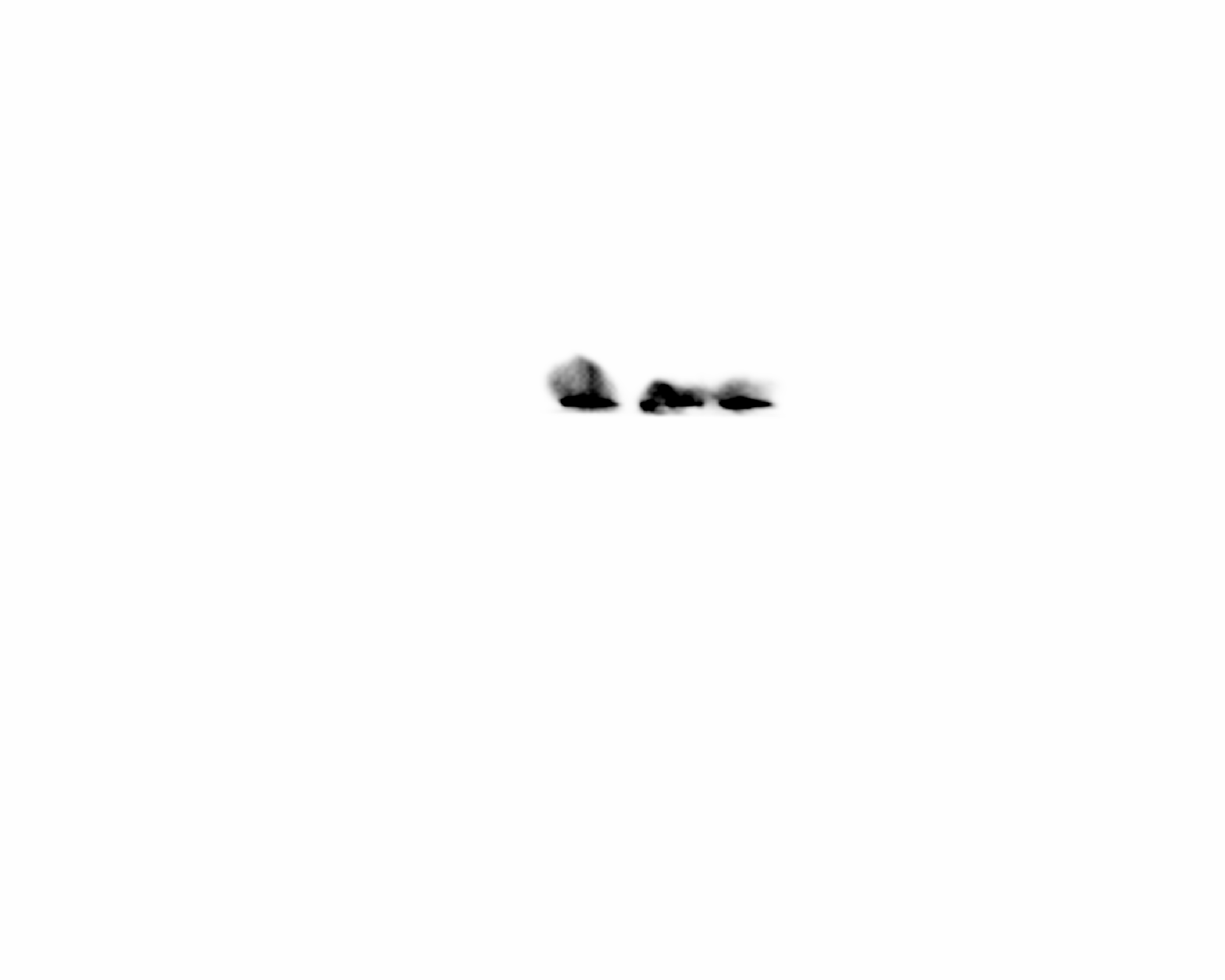

Supplement: Supplemental Information 3 [file peerj-12-18324-s003.zip › pstat1+stat1 1_2(Chemiluminescence).tif]

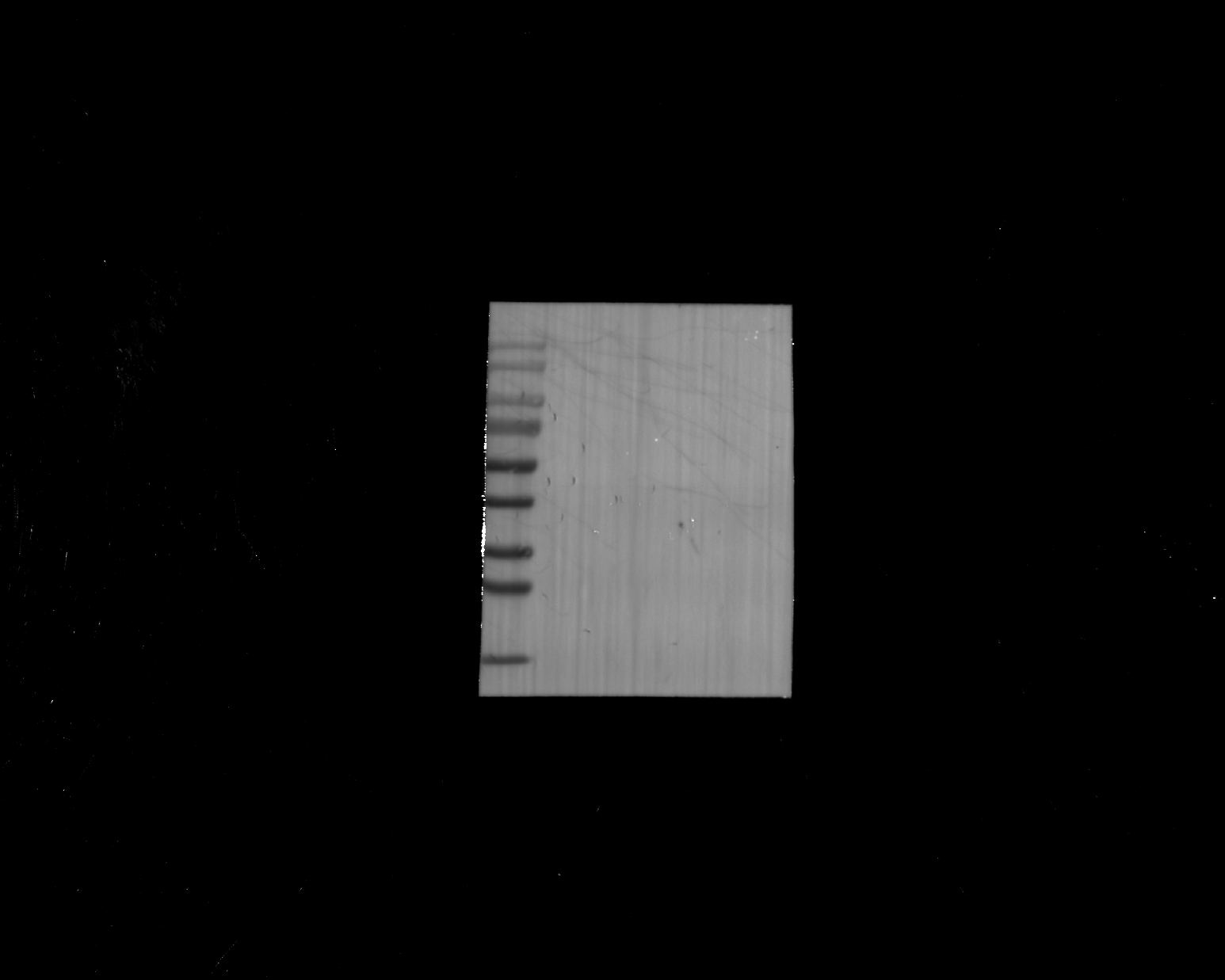

Supplement: Supplemental Information 3 [file peerj-12-18324-s003.zip › pstat1+stat1 1_2(Colorimetric).tif]

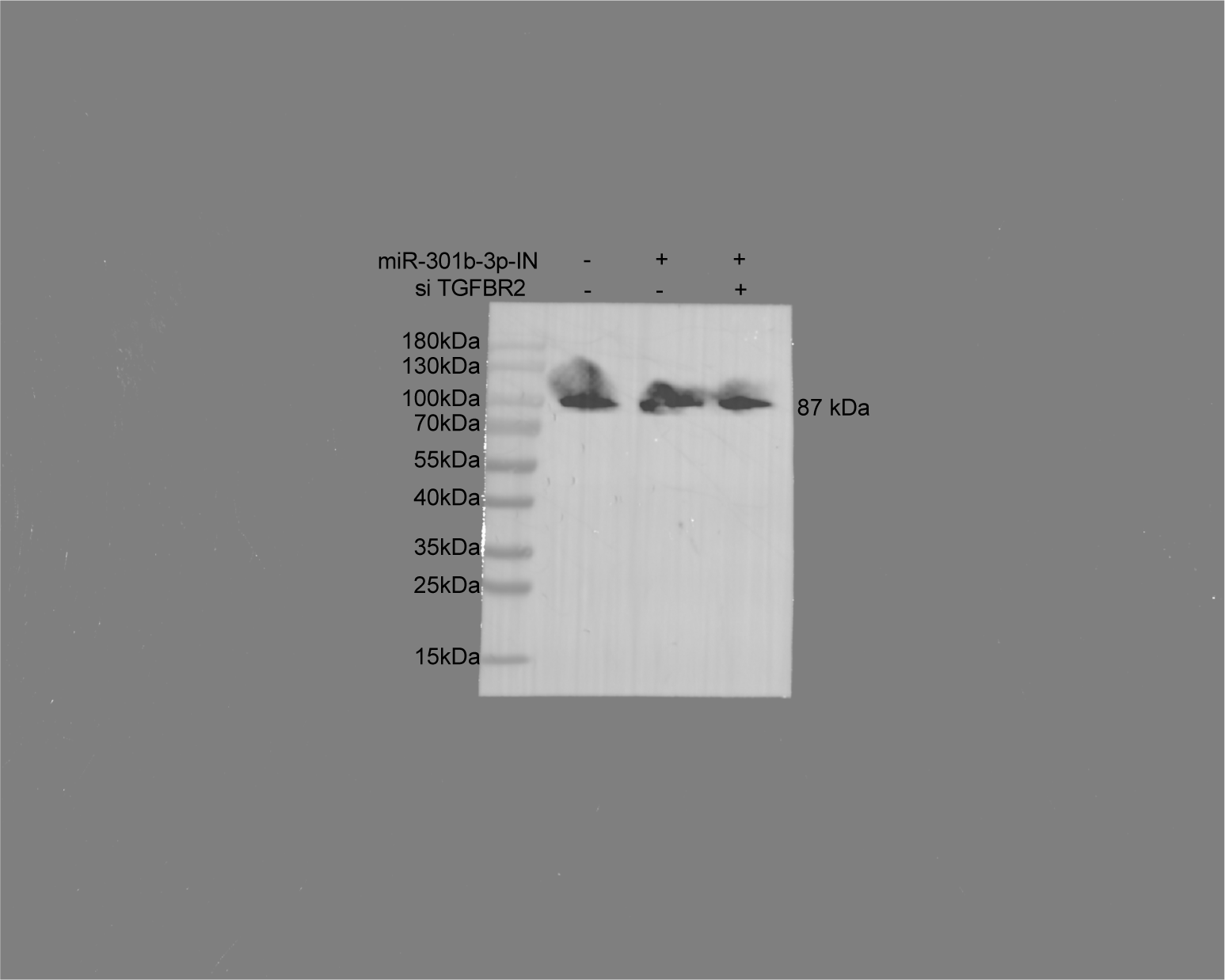

Supplement: Supplemental Information 3 [file peerj-12-18324-s003.zip › pstat1+stat1 1_2(Composite)-01.tif]

|                        |   |   |   |
|------------------------|---|---|---|
| miR-301b-3p-IN         | - | + | + |
| si TGFB <sup>R</sup> 2 | - | - | + |

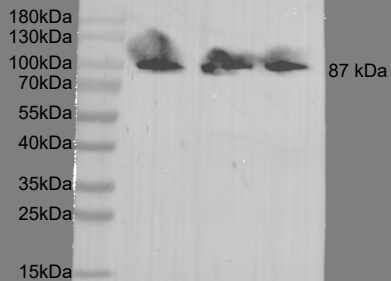

Supplement: Supplemental Information 3 [file peerj-12-18324-s003.zip › pstat1+stat1 1_2(Composite).pdf]

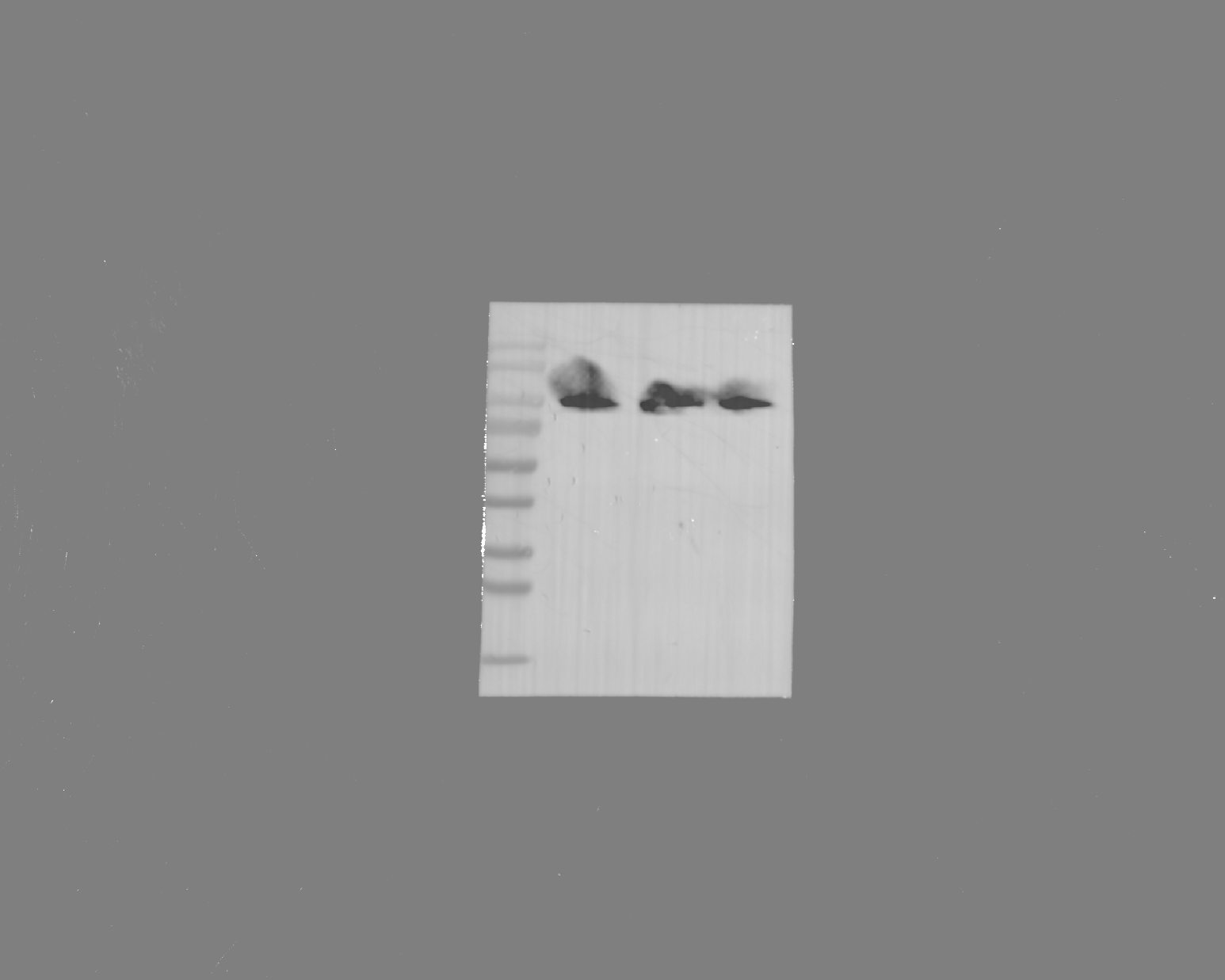

Supplement: Supplemental Information 3 [file peerj-12-18324-s003.zip › pstat1+stat1 1_2(Composite).tif]
