# Supplementary figures and images for: MiR-301b-3p promotes breast cancer development through inhibiting the expression of transforming growth factor-beta receptor 2 (part 2 of 2)
Source: PeerJ. 2024 Nov 5;12:e18324. doi: 10.7717/peerj.18324 (PMC11546148; doi:10.7717/peerj.18324)

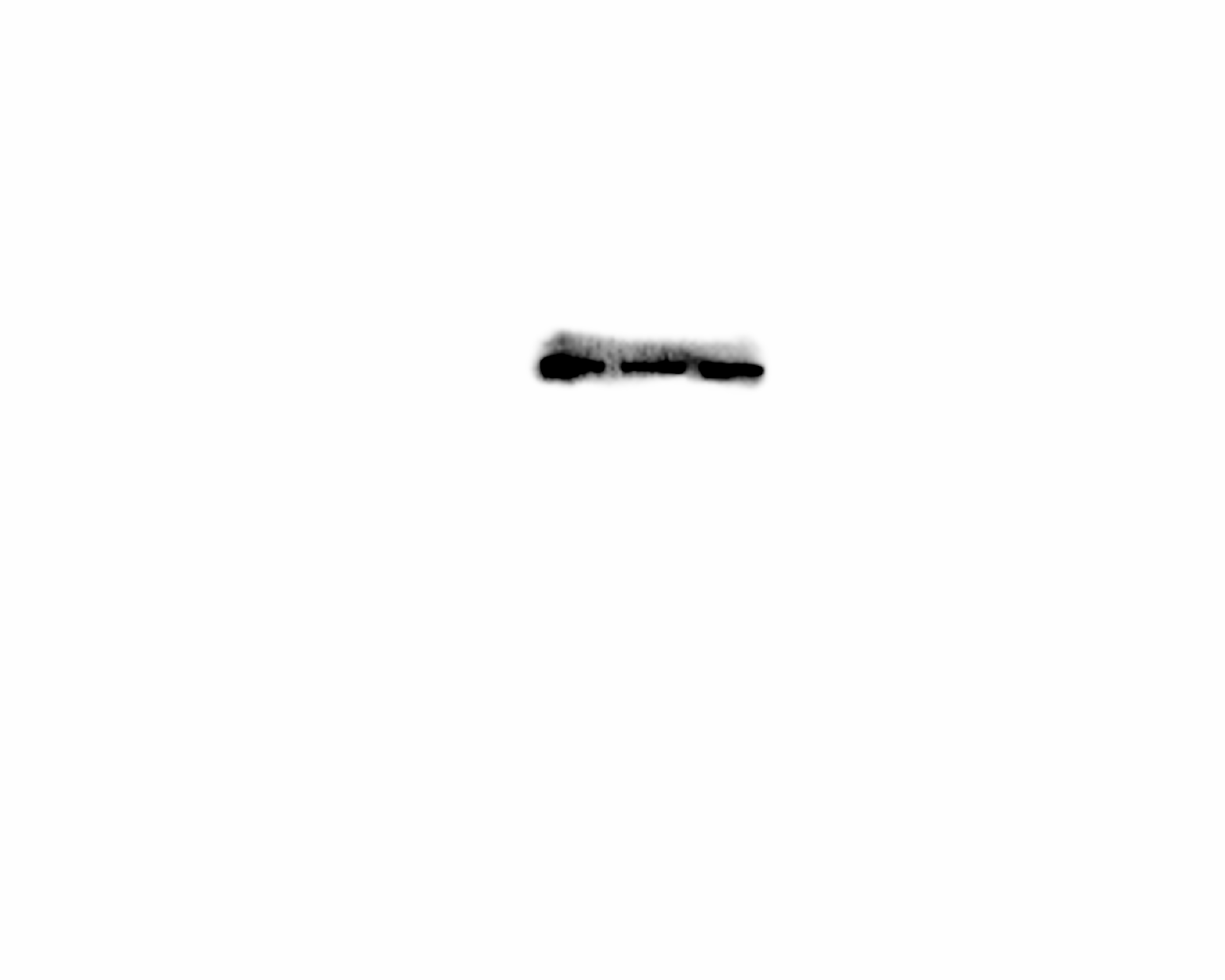

Supplement: Supplemental Information 3 [file peerj-12-18324-s003.zip › pstat1+stat1 2_1(Chemiluminescence).tif]

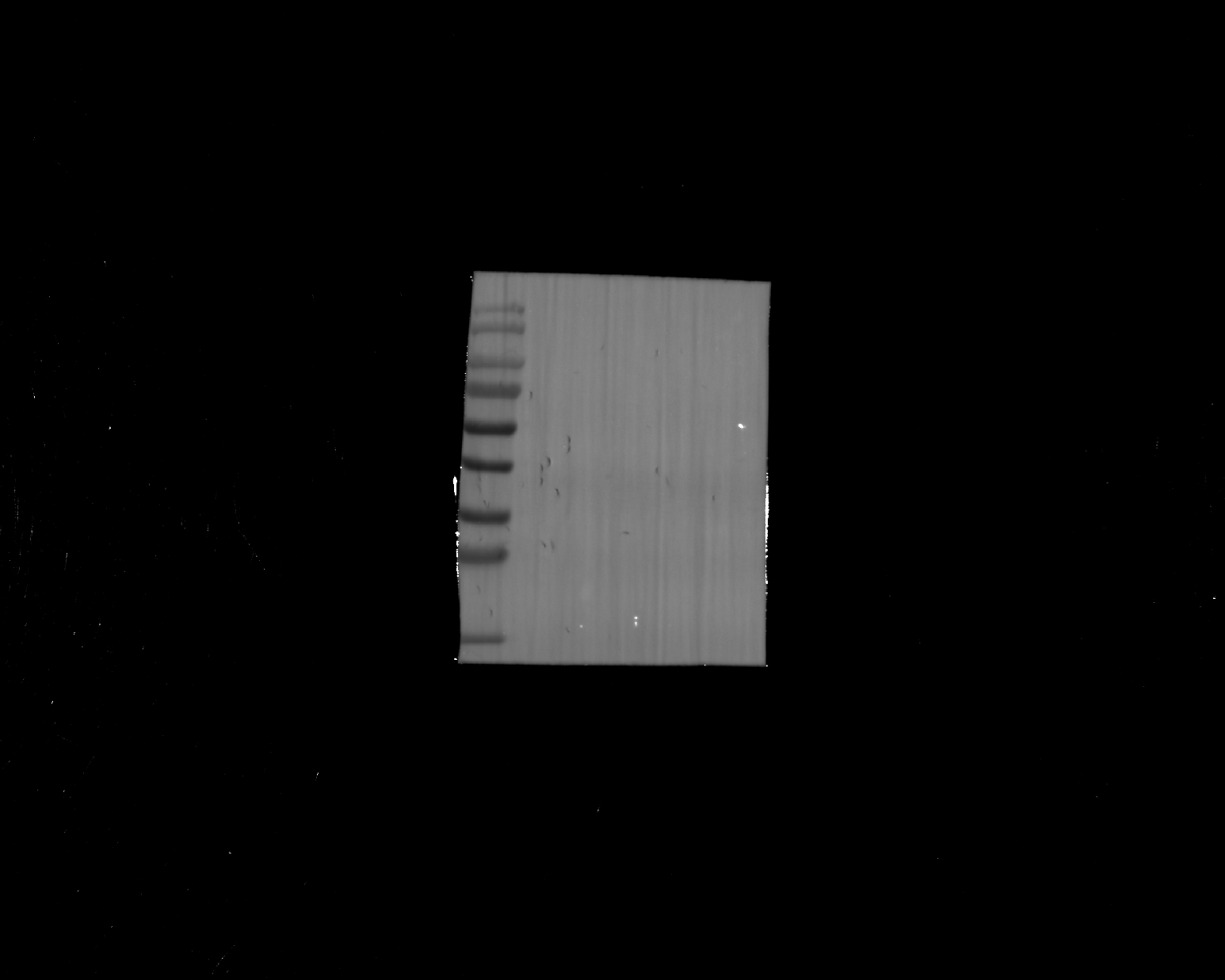

Supplement: Supplemental Information 3 [file peerj-12-18324-s003.zip › pstat1+stat1 2_1(Colorimetric).tif]

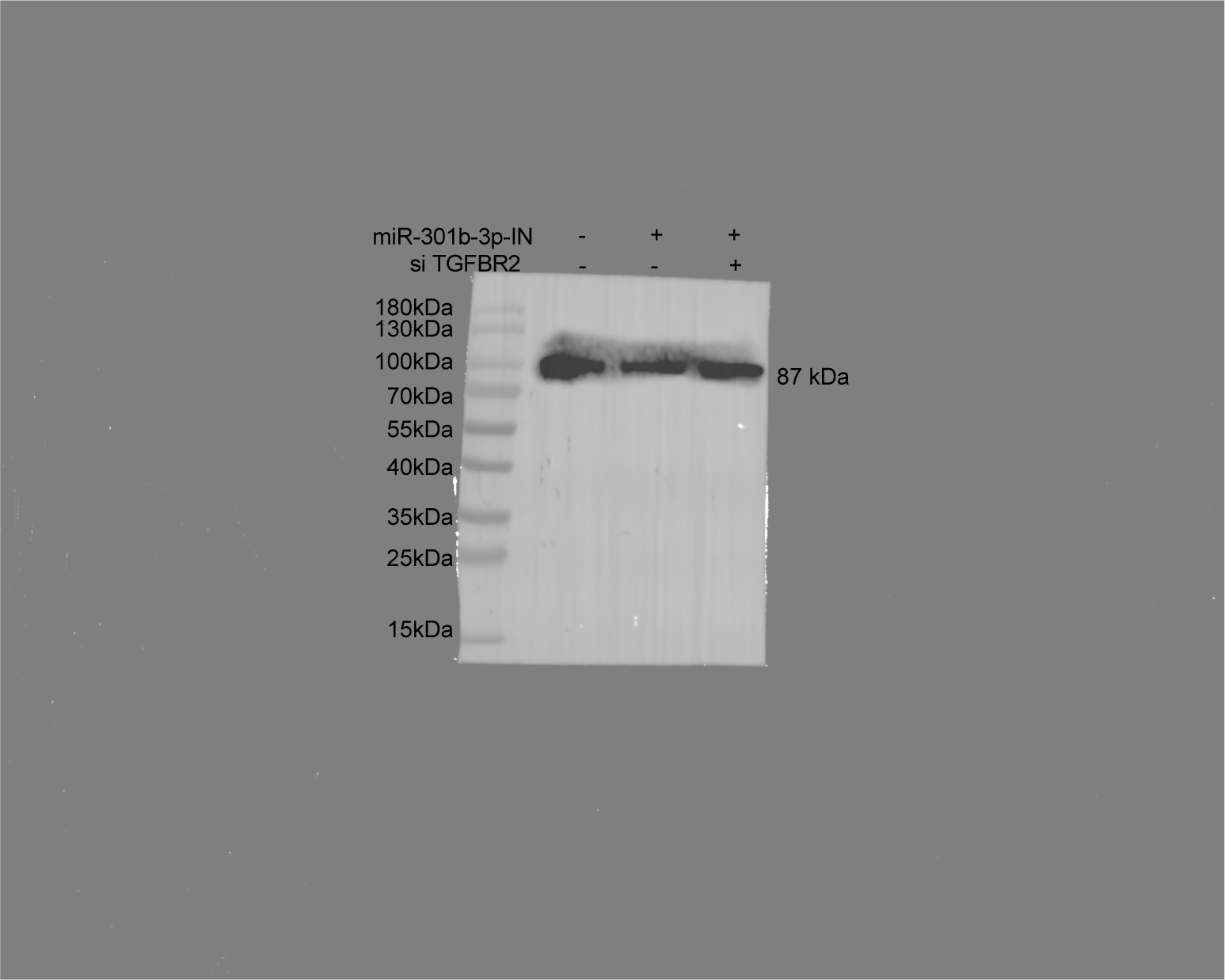

Supplement: Supplemental Information 3 [file peerj-12-18324-s003.zip › pstat1+stat1 2_1(Composite)-01.tif]

|                |   |   |   |
|----------------|---|---|---|
| miR-301b-3p-IN | - | + | + |
| si TGFBR2      | - | - | + |

180kDa  
130kDa  
100kDa  
70kDa  
55kDa  
40kDa  
35kDa  
25kDa  
15kDa

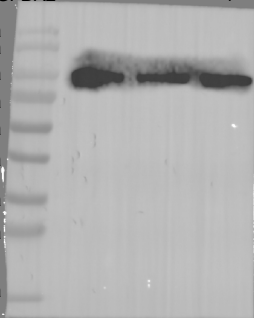

87 kDa

Supplement: Supplemental Information 3 [file peerj-12-18324-s003.zip › pstat1+stat1 2_1(Composite).pdf]

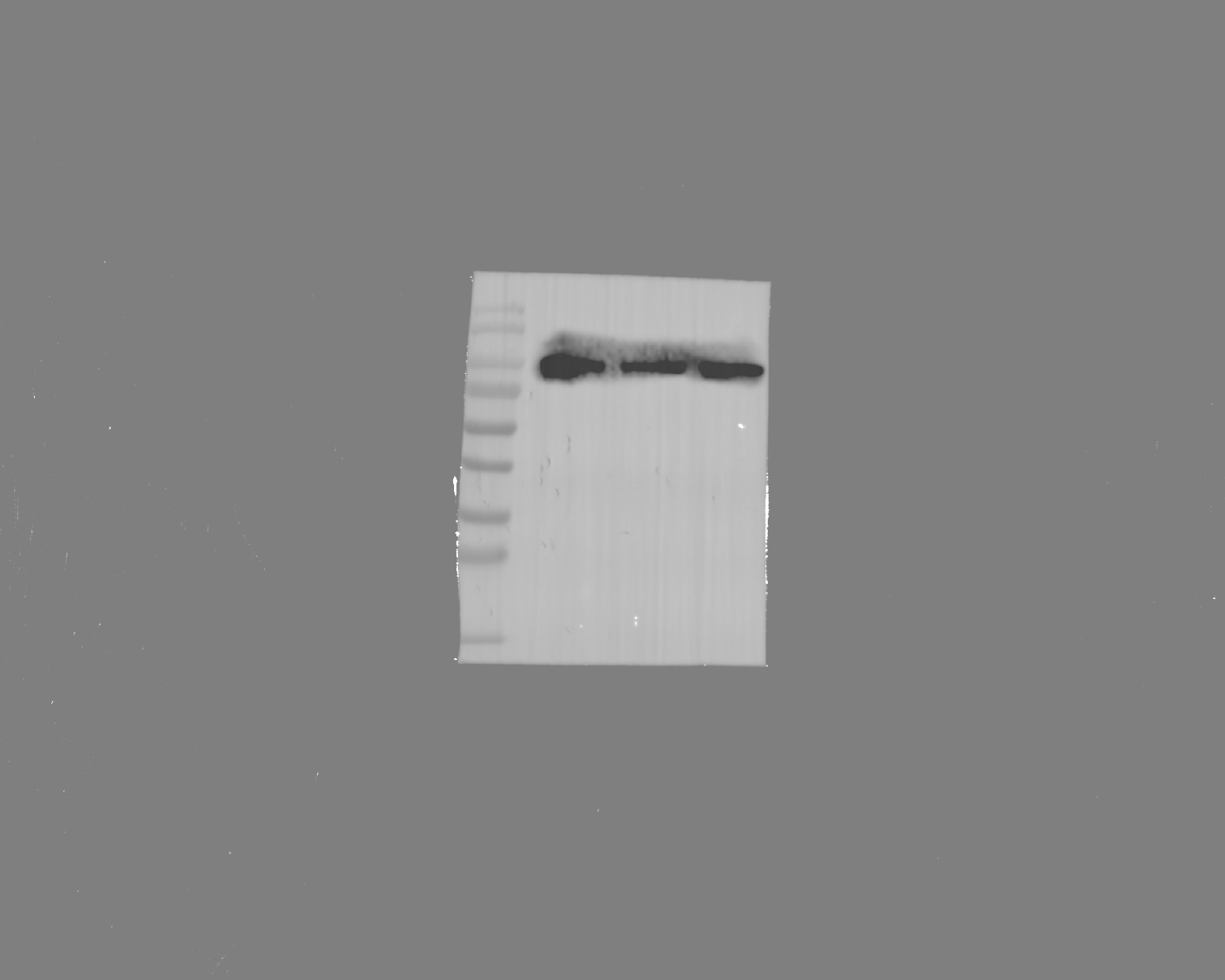

Supplement: Supplemental Information 3 [file peerj-12-18324-s003.zip › pstat1+stat1 2_1(Composite).tif]

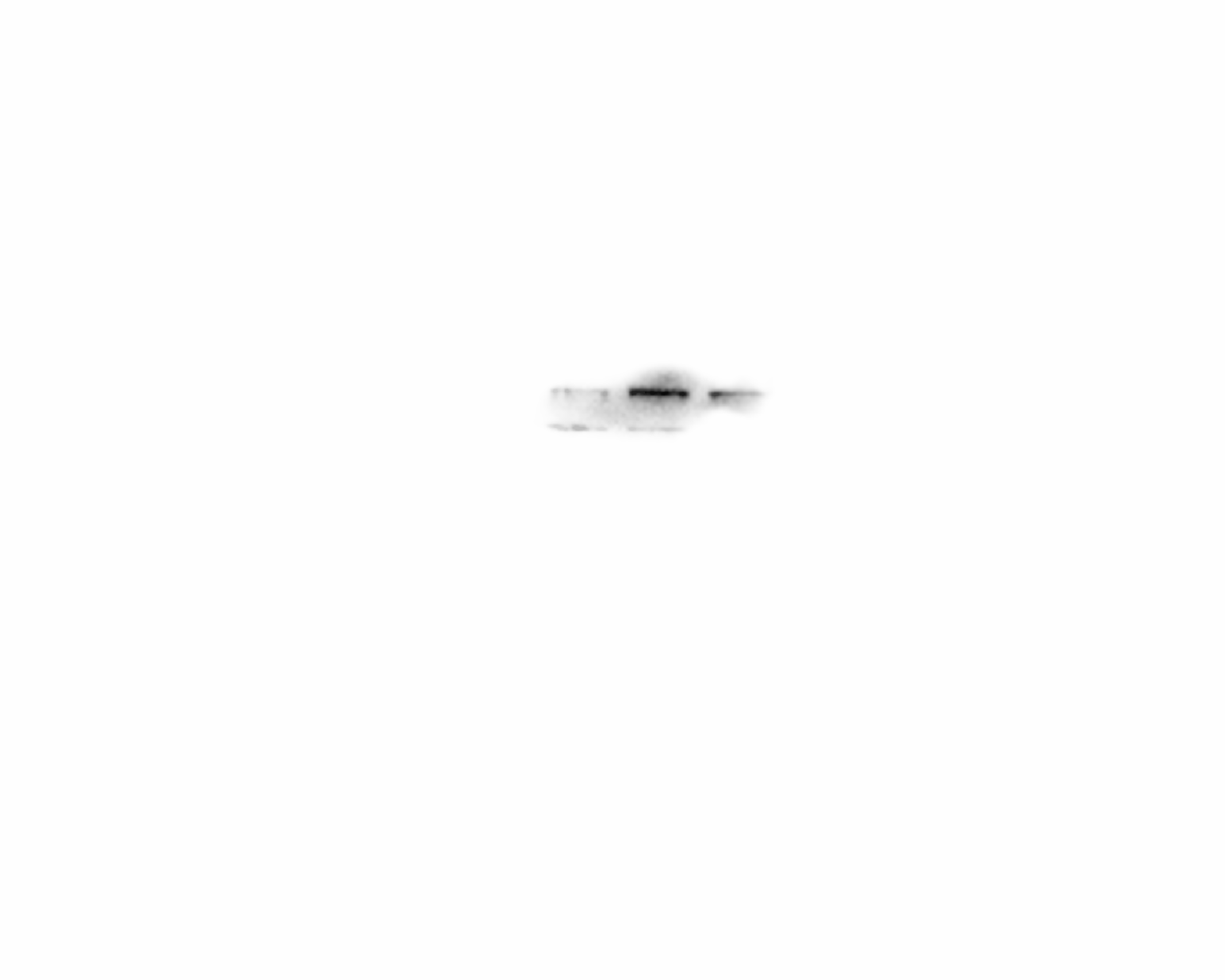

Supplement: Supplemental Information 3 [file peerj-12-18324-s003.zip › pstat1+stat1 2_2(Chemiluminescence).tif]

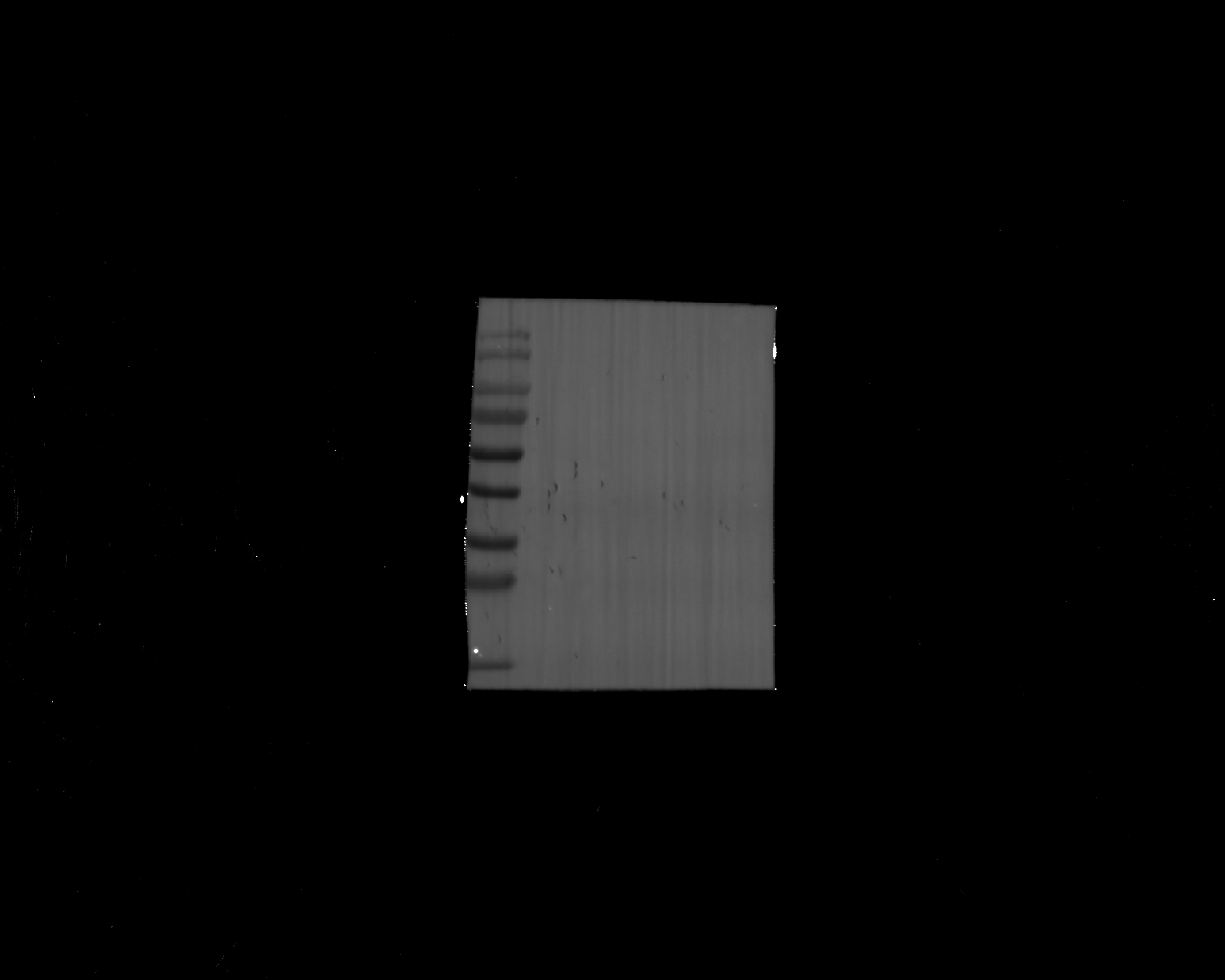

Supplement: Supplemental Information 3 [file peerj-12-18324-s003.zip › pstat1+stat1 2_2(Colorimetric).tif]

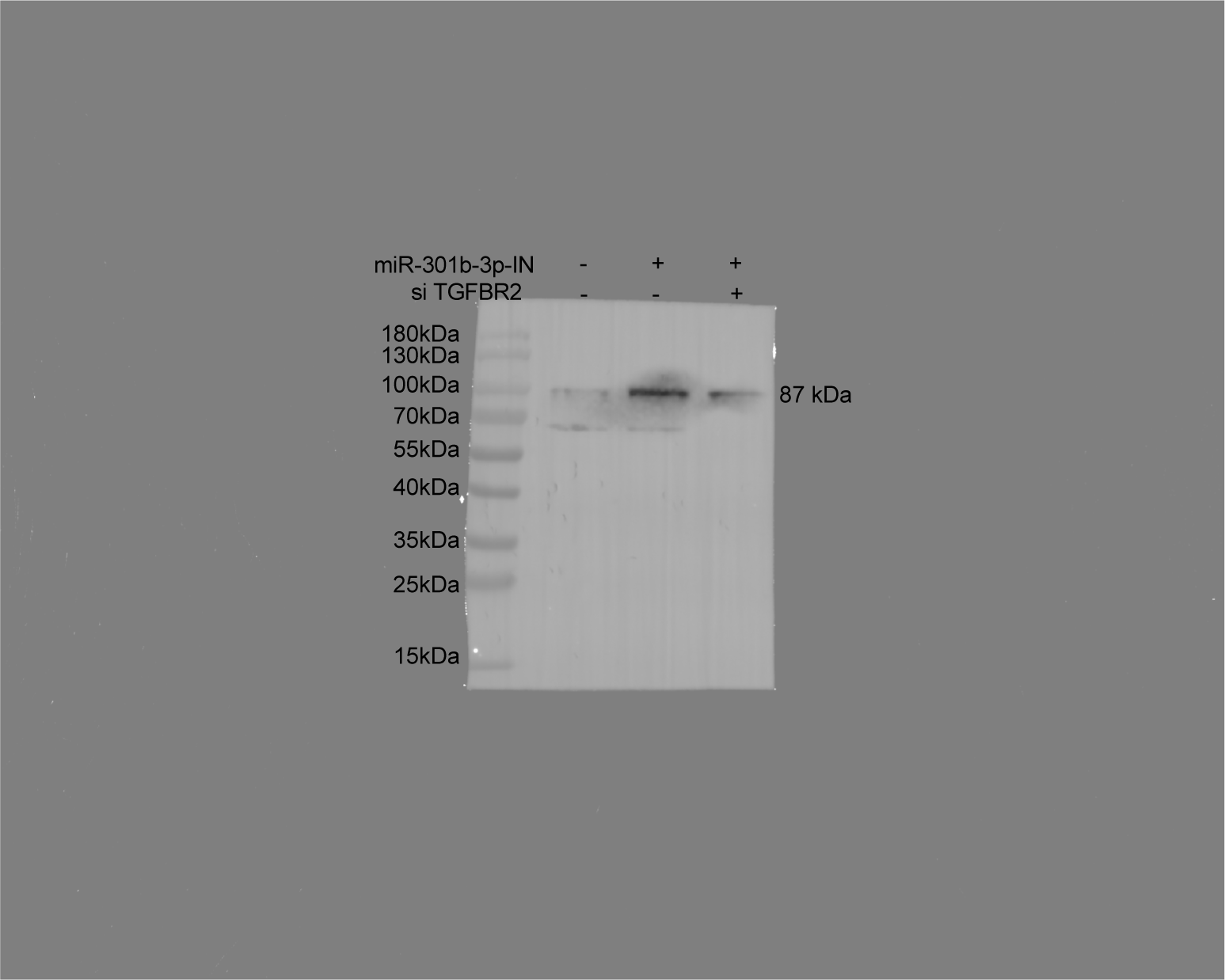

Supplement: Supplemental Information 3 [file peerj-12-18324-s003.zip › pstat1+stat1 2_2(Composite)-01.tif]

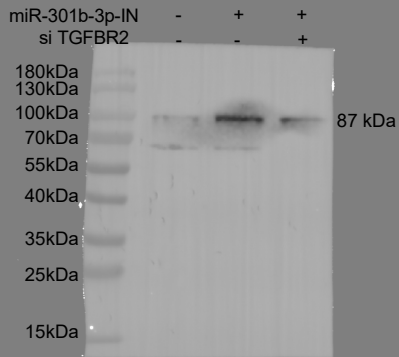

Supplement: Supplemental Information 3 [file peerj-12-18324-s003.zip › pstat1+stat1 2_2(Composite).pdf]

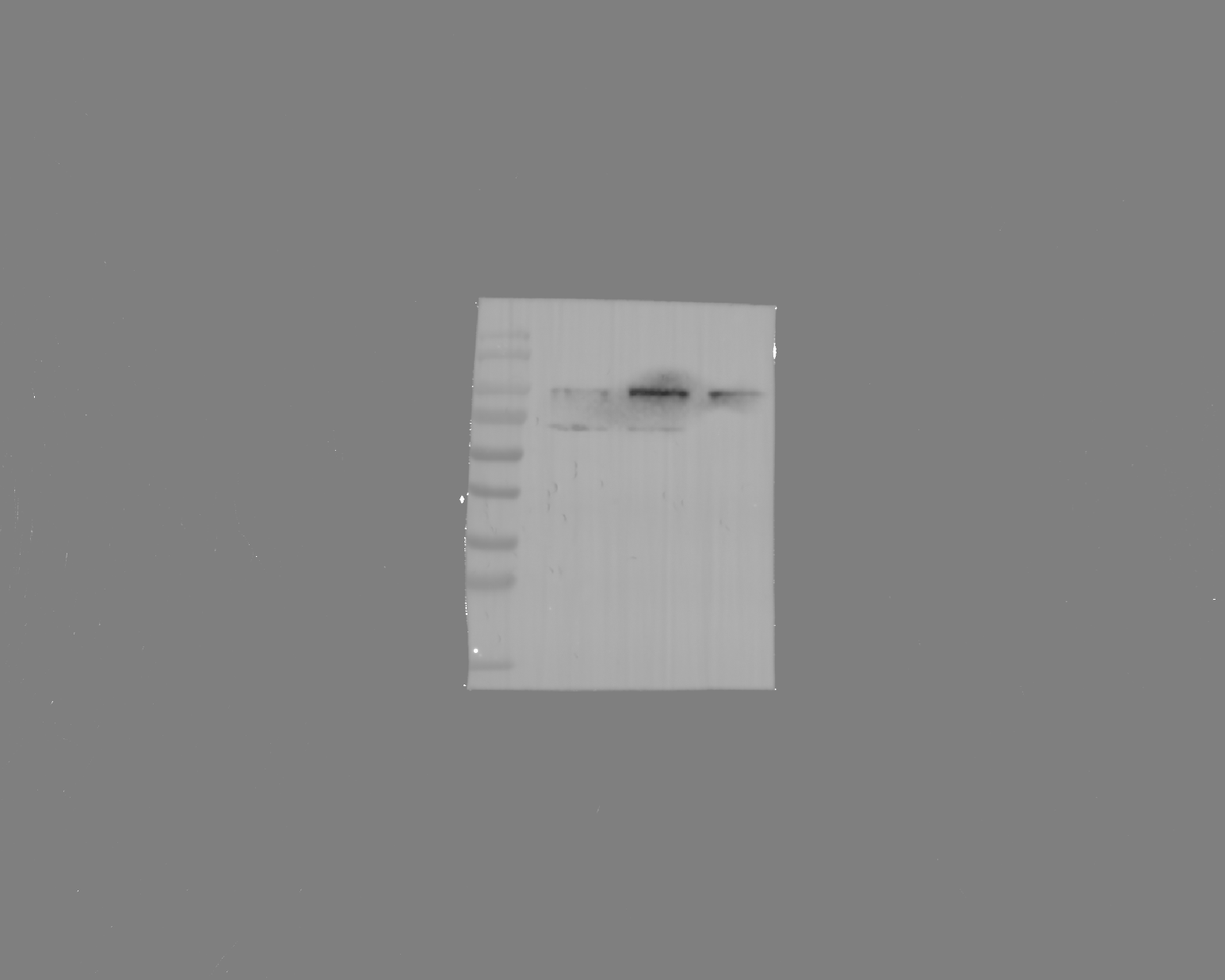

Supplement: Supplemental Information 3 [file peerj-12-18324-s003.zip › pstat1+stat1 2_2(Composite).tif]

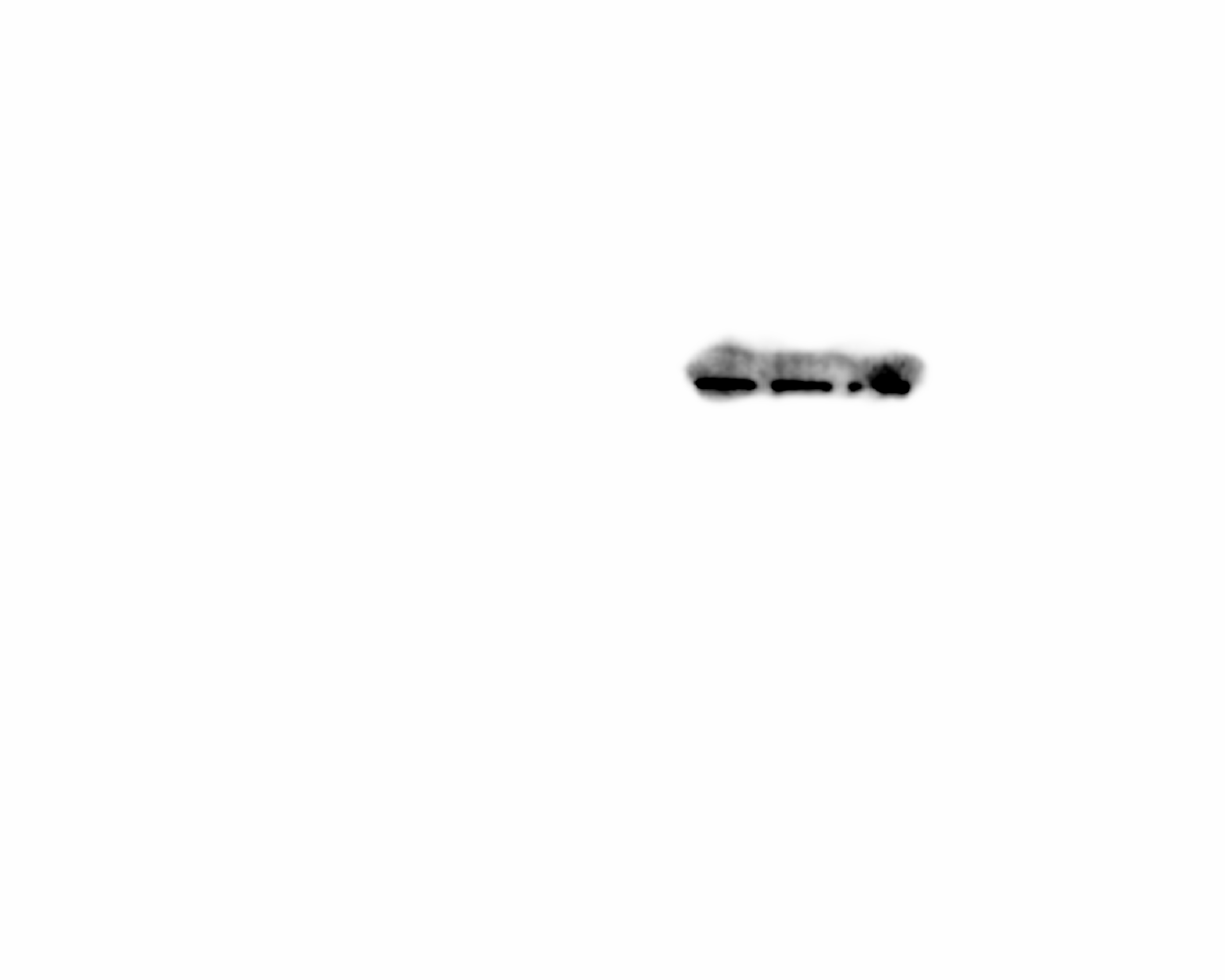

Supplement: Supplemental Information 3 [file peerj-12-18324-s003.zip › pstat1+stat1 3_1(Chemiluminescence).tif]

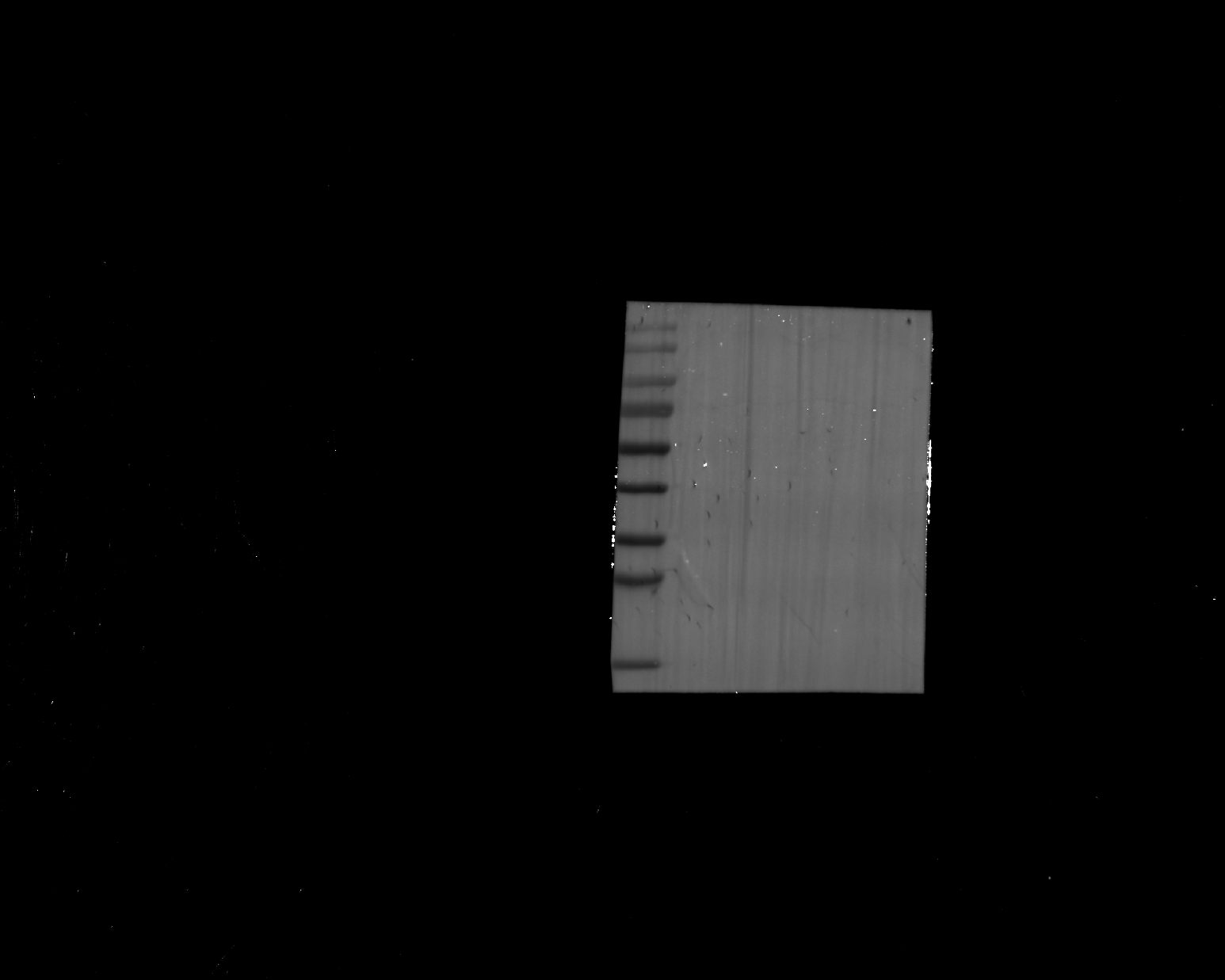

Supplement: Supplemental Information 3 [file peerj-12-18324-s003.zip › pstat1+stat1 3_1(Colorimetric).tif]

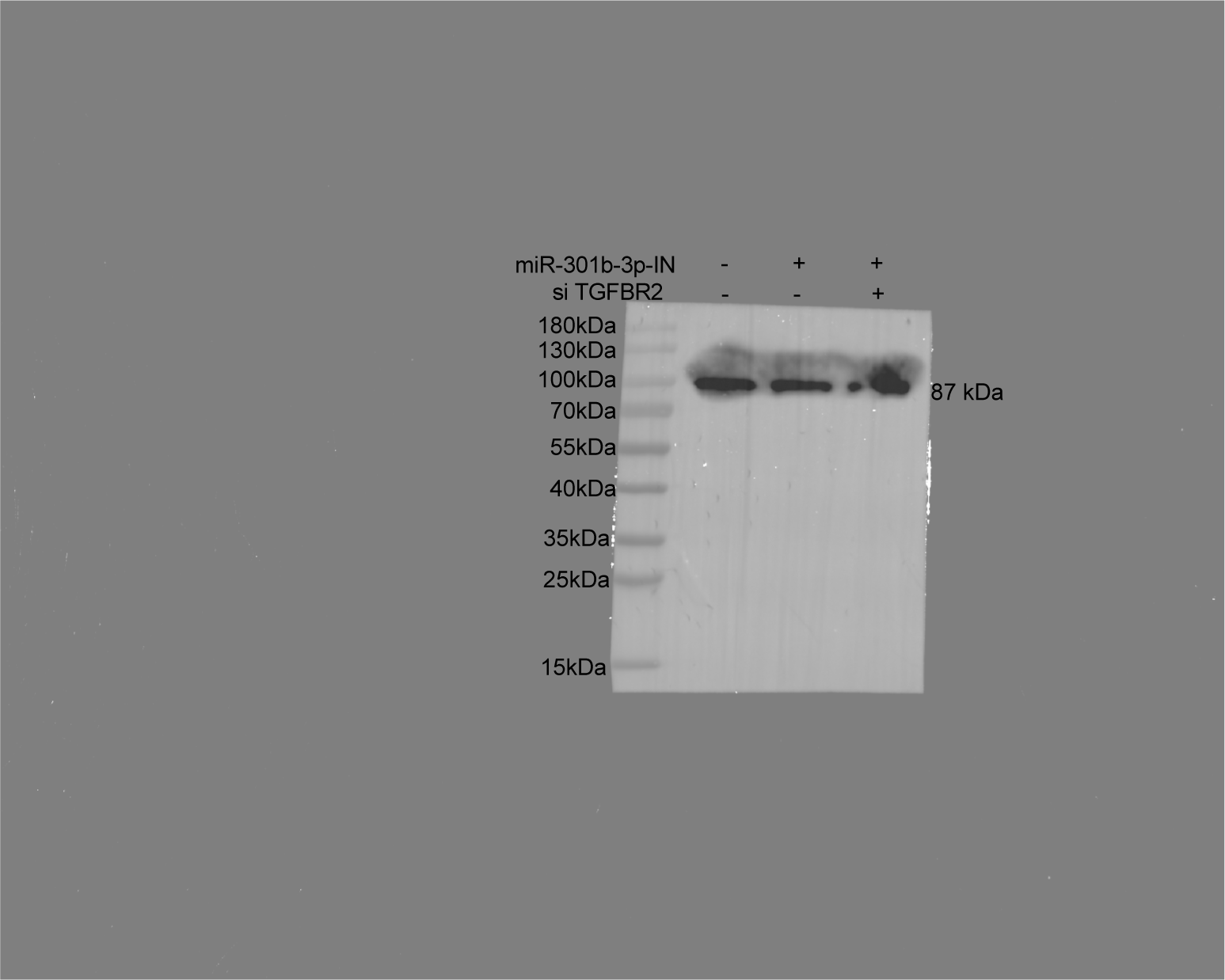

Supplement: Supplemental Information 3 [file peerj-12-18324-s003.zip › pstat1+stat1 3_1(Composite)-01.tif]

miR-301b-3p-IN

-

+

+

si TGFB $\beta$ 2

-

-

+

180kDa

130kDa

100kDa

70kDa

55kDa

40kDa

35kDa

25kDa

15kDa

87 kDa

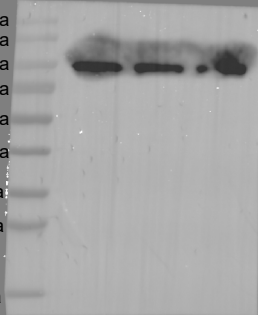

Supplement: Supplemental Information 3 [file peerj-12-18324-s003.zip › pstat1+stat1 3_1(Composite).pdf]

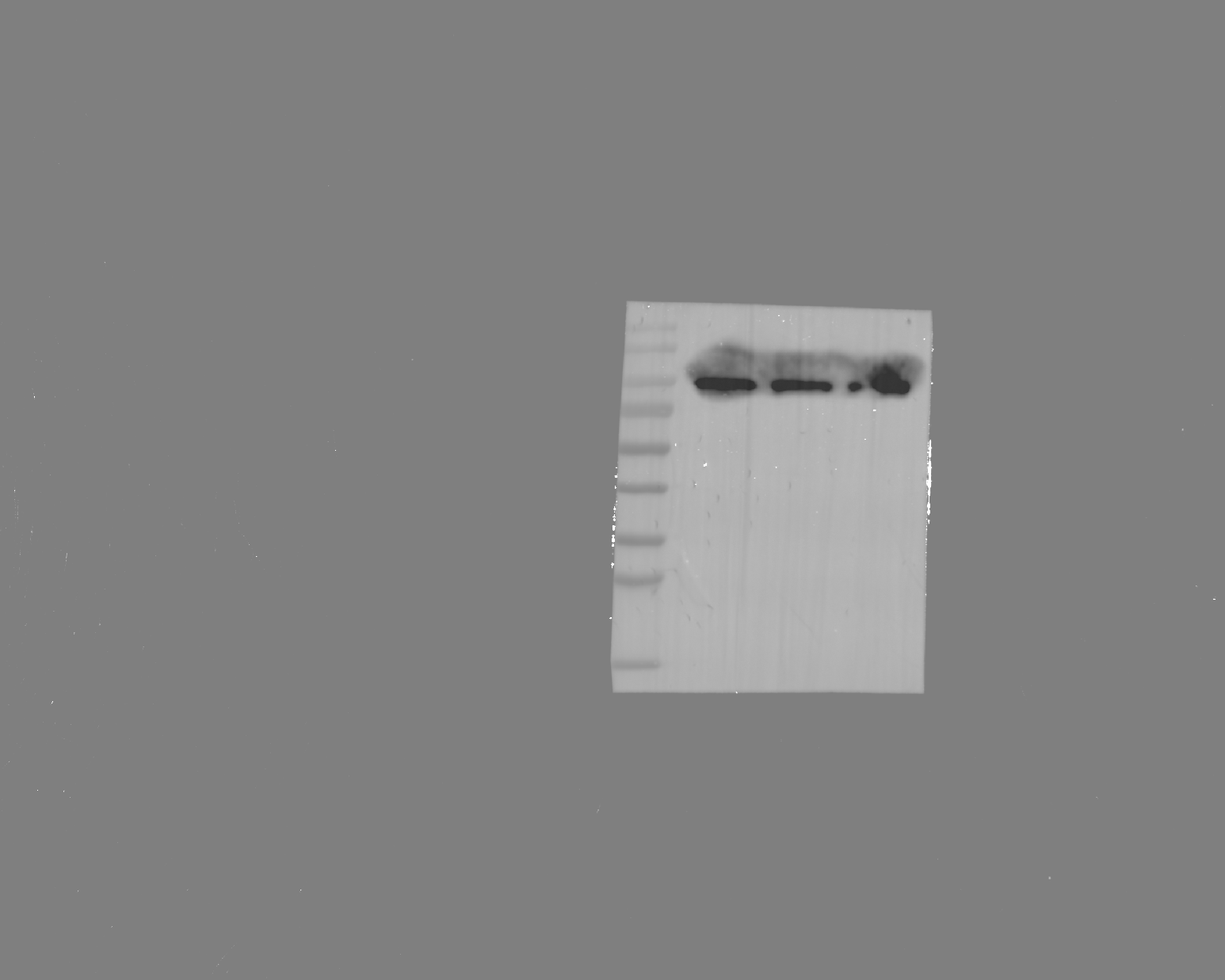

Supplement: Supplemental Information 3 [file peerj-12-18324-s003.zip › pstat1+stat1 3_1(Composite).tif]

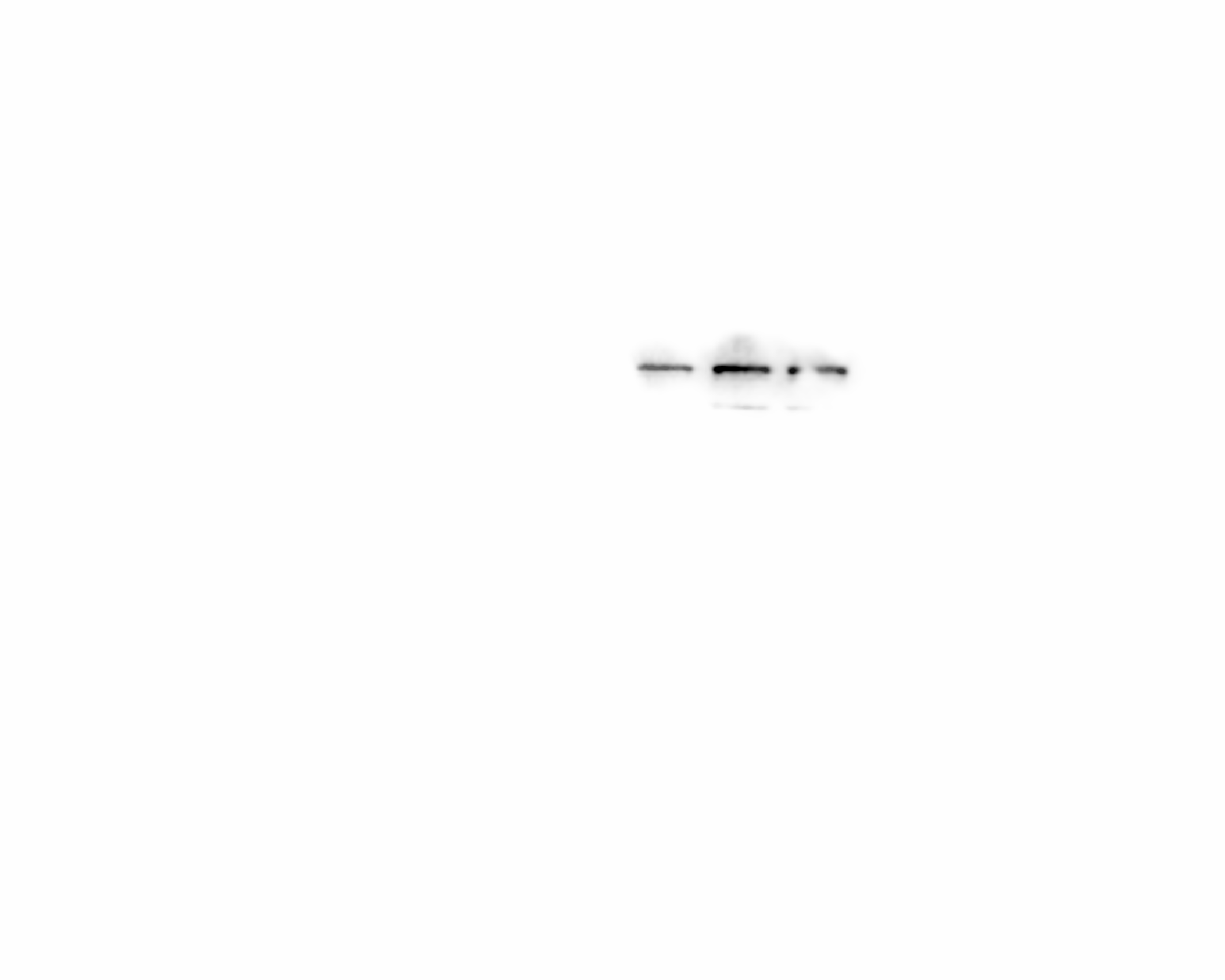

Supplement: Supplemental Information 3 [file peerj-12-18324-s003.zip › pstat1+stat1 3_2(Chemiluminescence).tif]

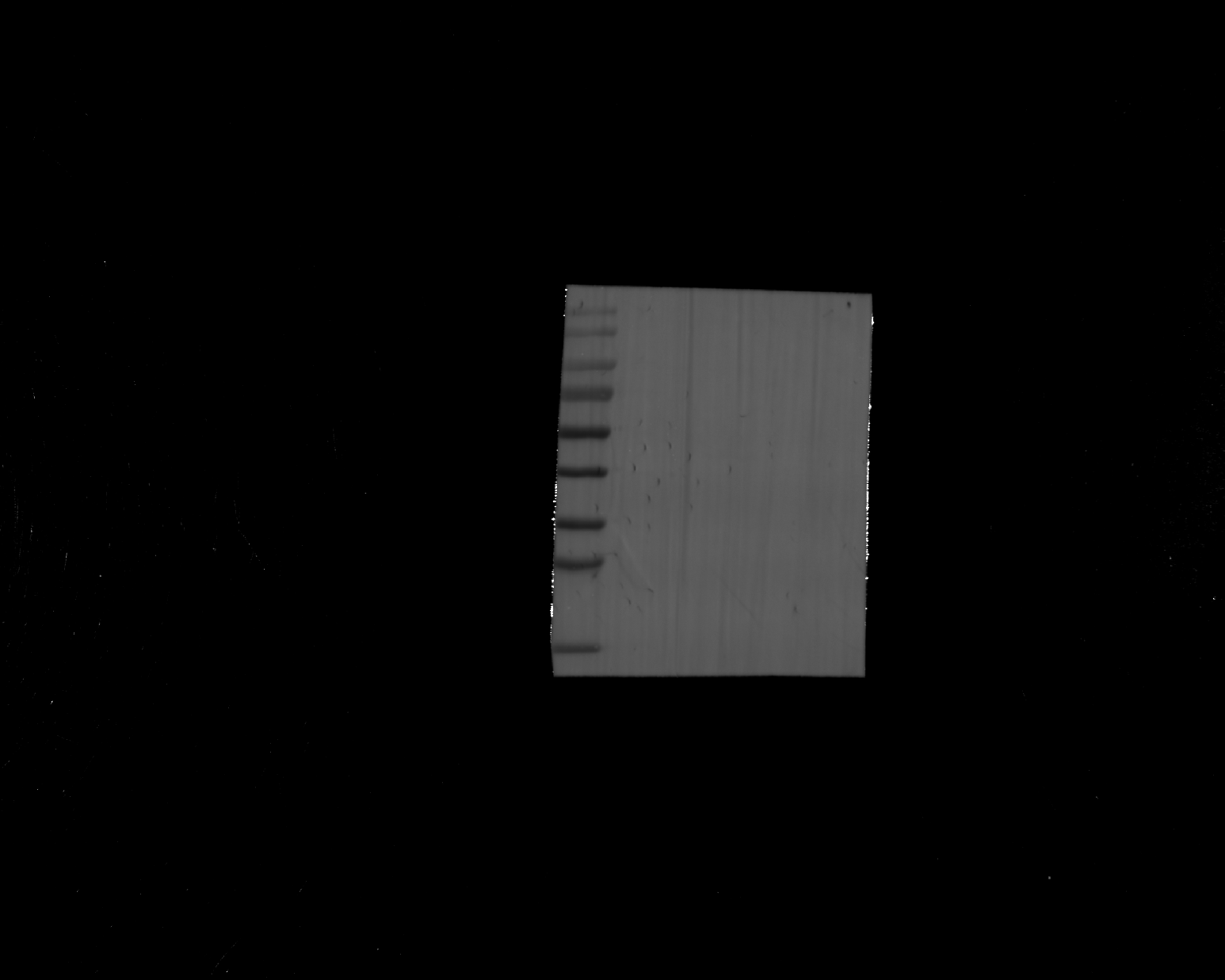

Supplement: Supplemental Information 3 [file peerj-12-18324-s003.zip › pstat1+stat1 3_2(Colorimetric).tif]

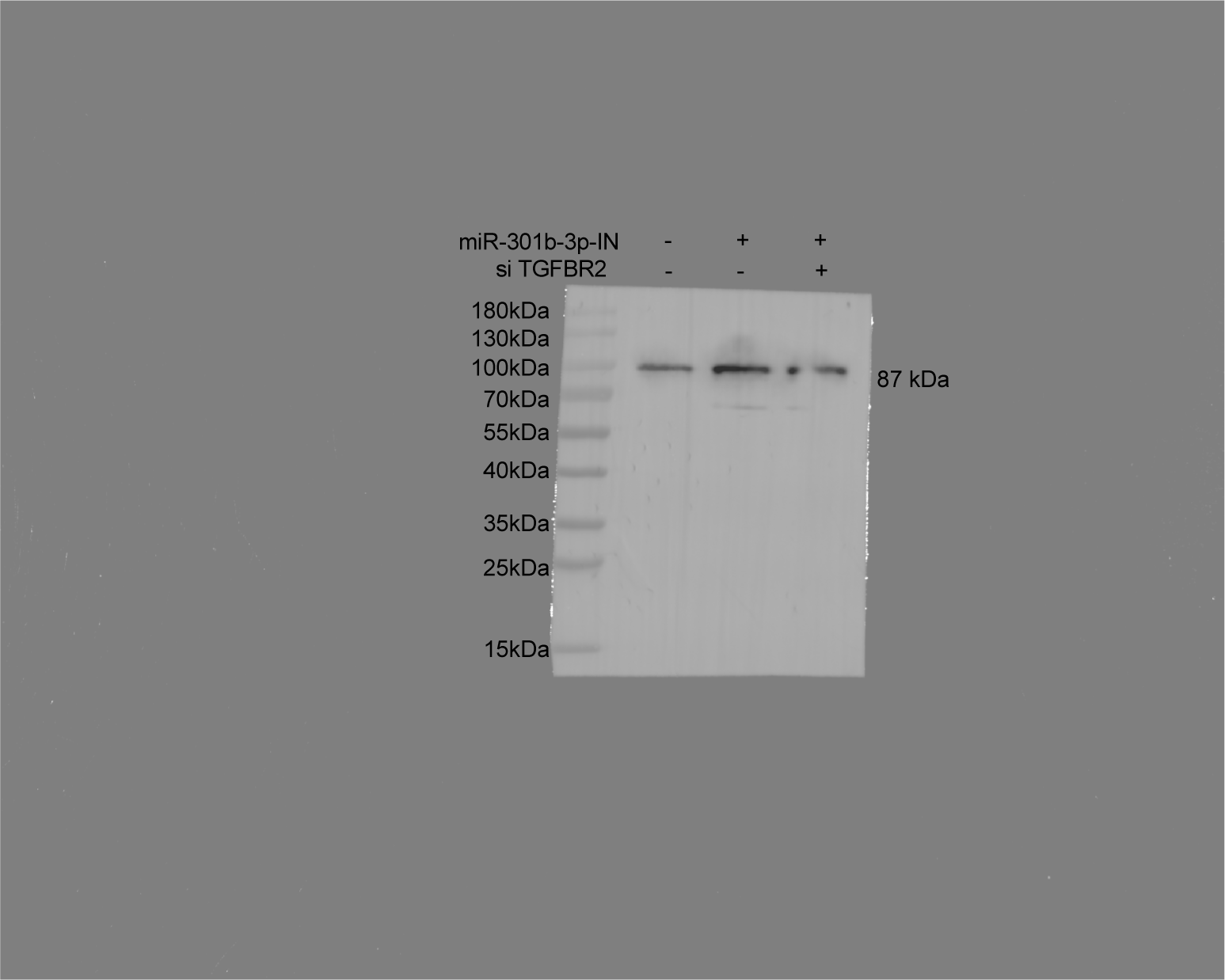

Supplement: Supplemental Information 3 [file peerj-12-18324-s003.zip › pstat1+stat1 3_2(Composite)-01.tif]

miR-301b-3p-IN  
si TGFR2

|   |   |   |
|---|---|---|
| - | + | + |
| - | - | + |

180kDa  
130kDa  
100kDa  
70kDa  
55kDa  
40kDa  
35kDa  
25kDa  
15kDa

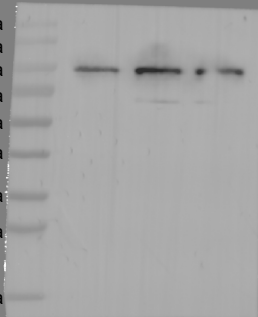

87 kDa

Supplement: Supplemental Information 3 [file peerj-12-18324-s003.zip › pstat1+stat1 3_2(Composite).pdf]

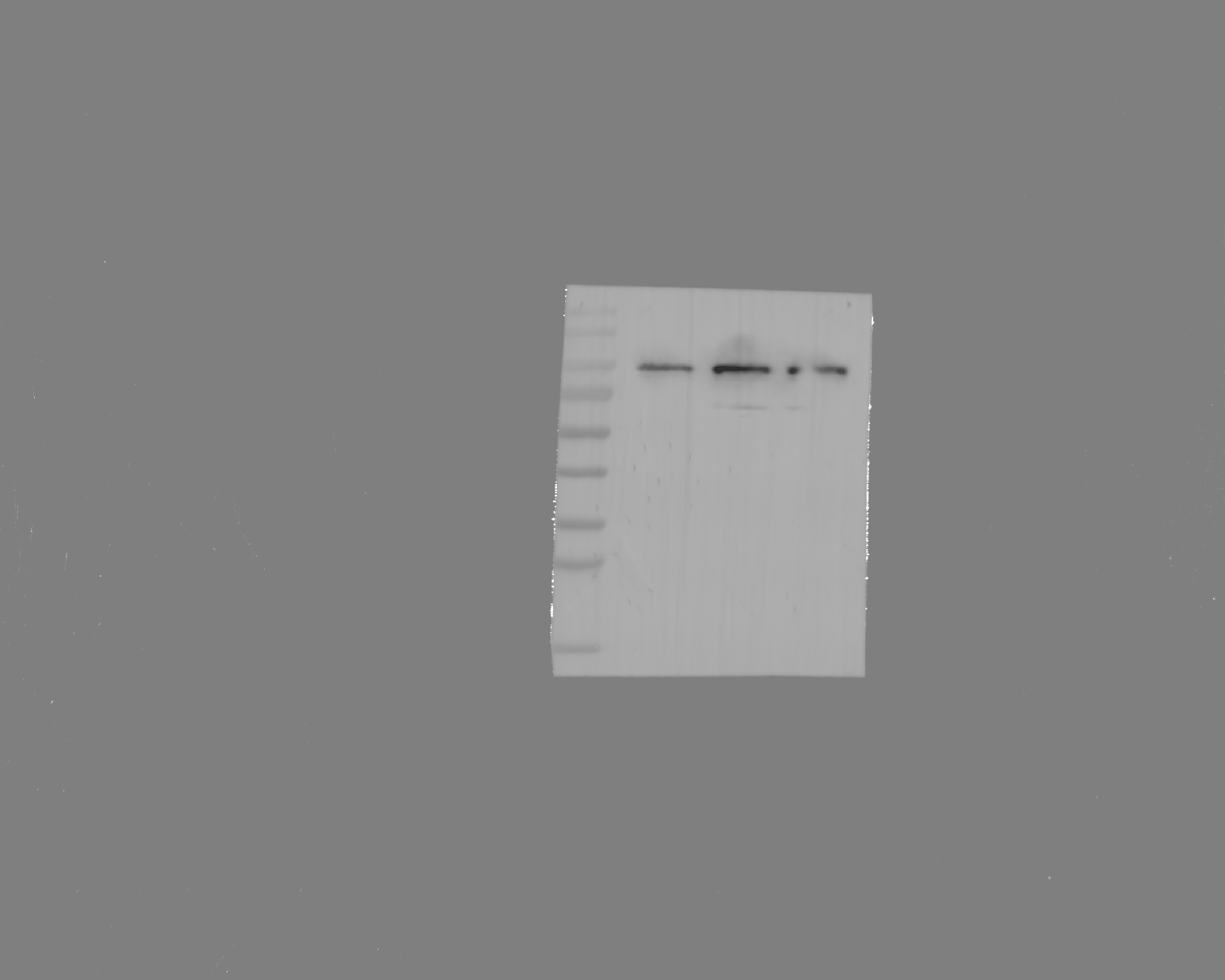

Supplement: Supplemental Information 3 [file peerj-12-18324-s003.zip › pstat1+stat1 3_2(Composite).tif]

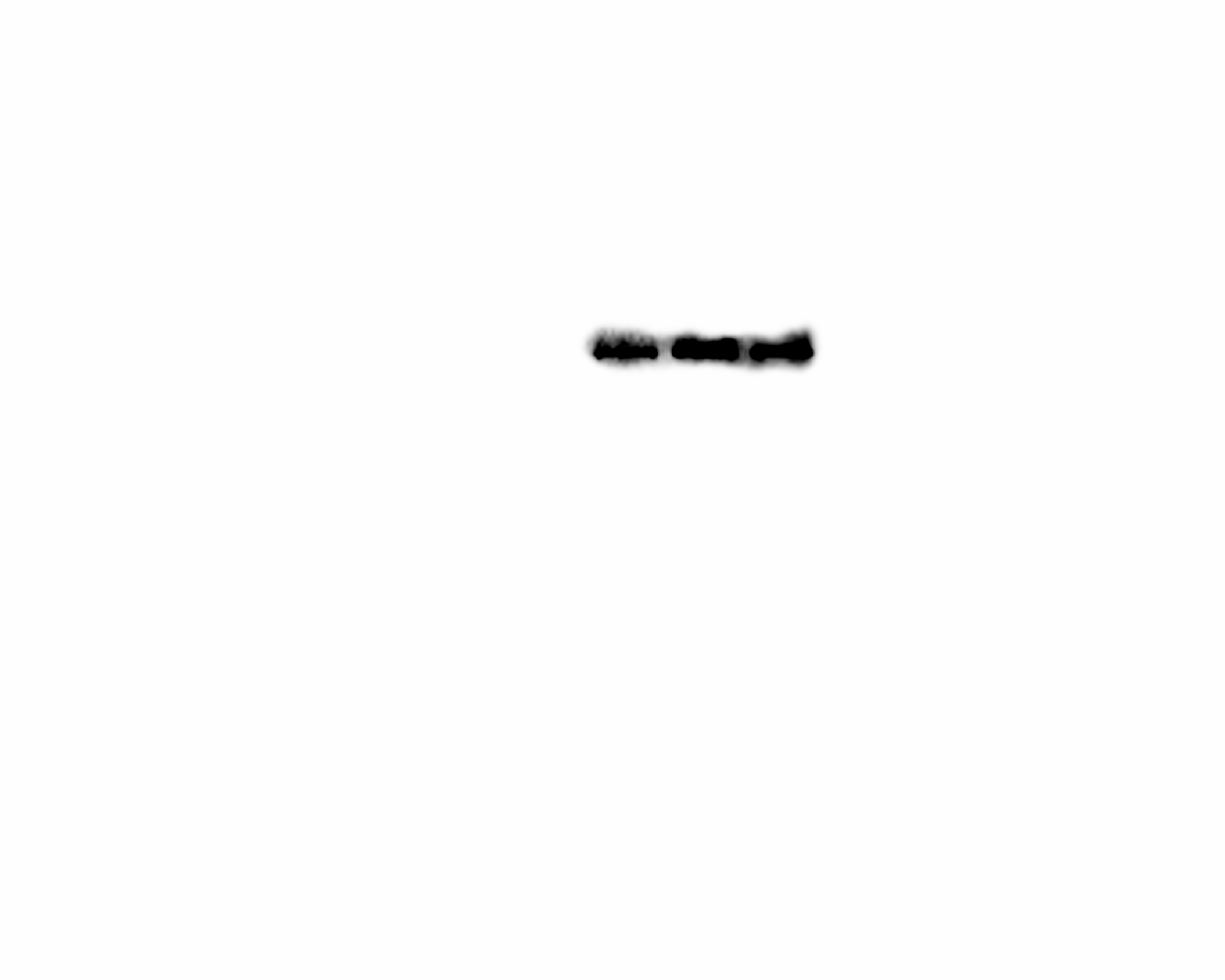

Supplement: Supplemental Information 4 [file peerj-12-18324-s004.zip › pstat3+stat3 1_1(Chemiluminescence).tif]

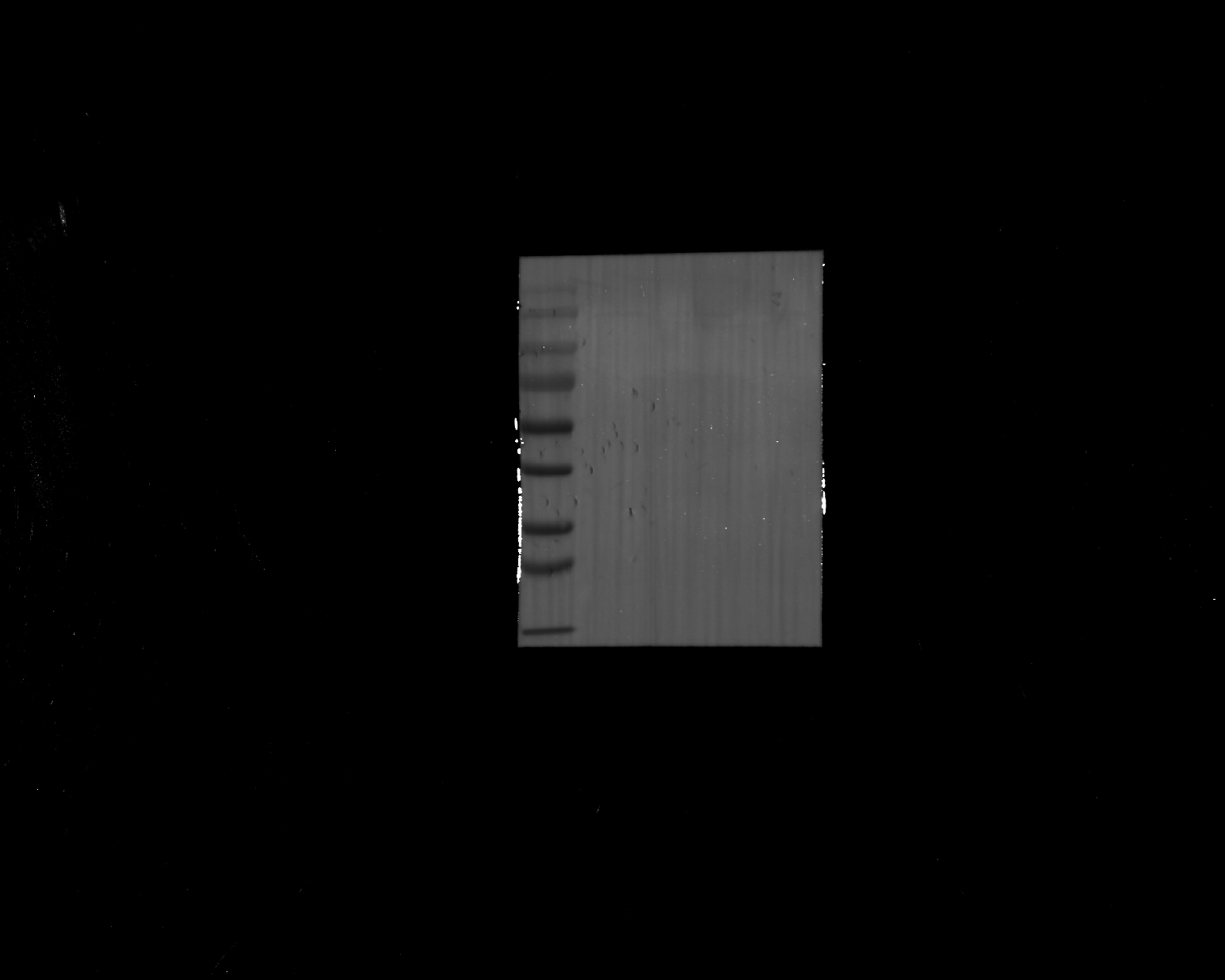

Supplement: Supplemental Information 4 [file peerj-12-18324-s004.zip › pstat3+stat3 1_1(Colorimetric).tif]

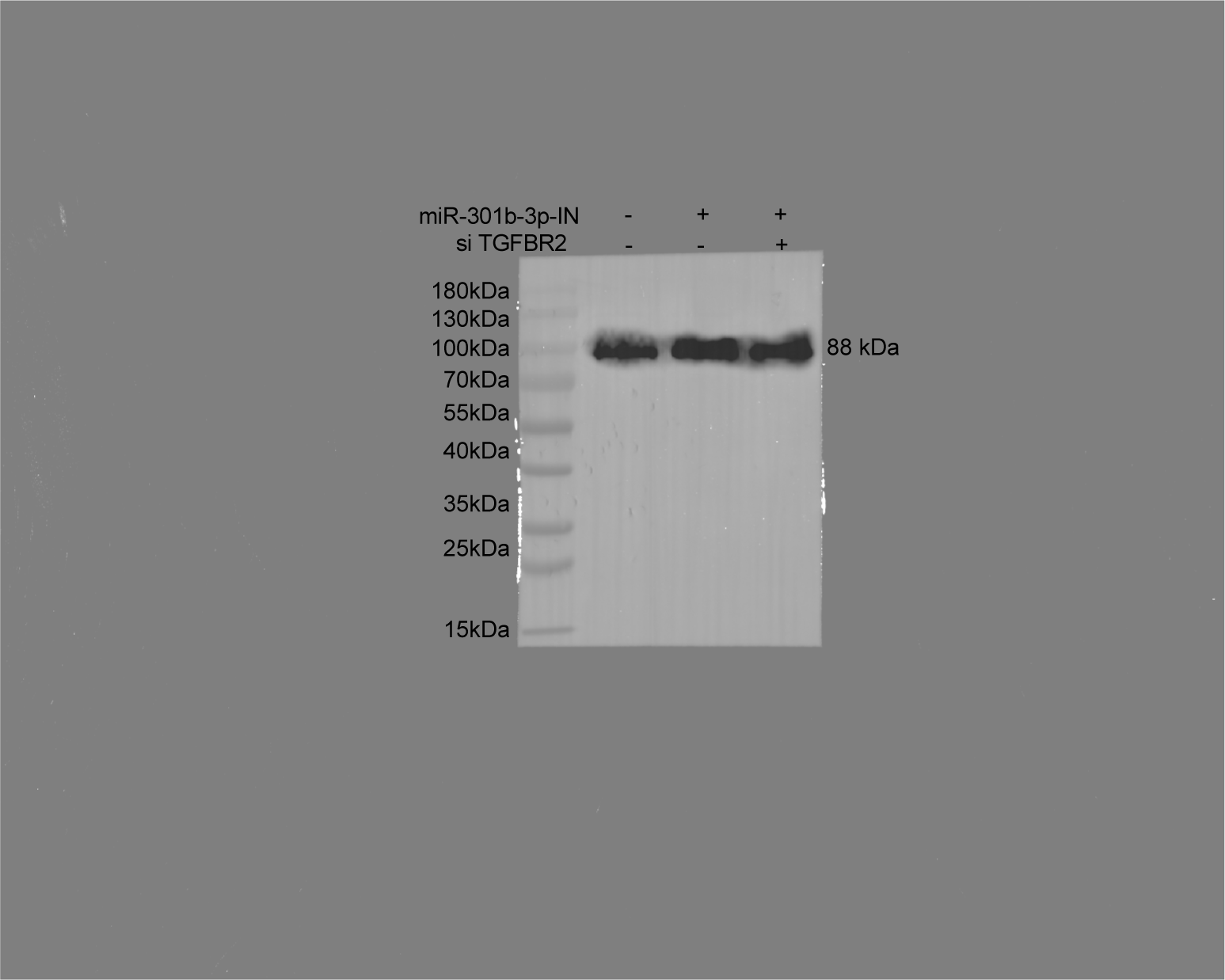

Supplement: Supplemental Information 4 [file peerj-12-18324-s004.zip › pstat3+stat3 1_1(Composite)-01.tif]

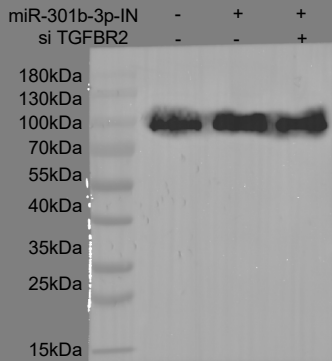

Supplement: Supplemental Information 4 [file peerj-12-18324-s004.zip › pstat3+stat3 1_1(Composite).pdf]

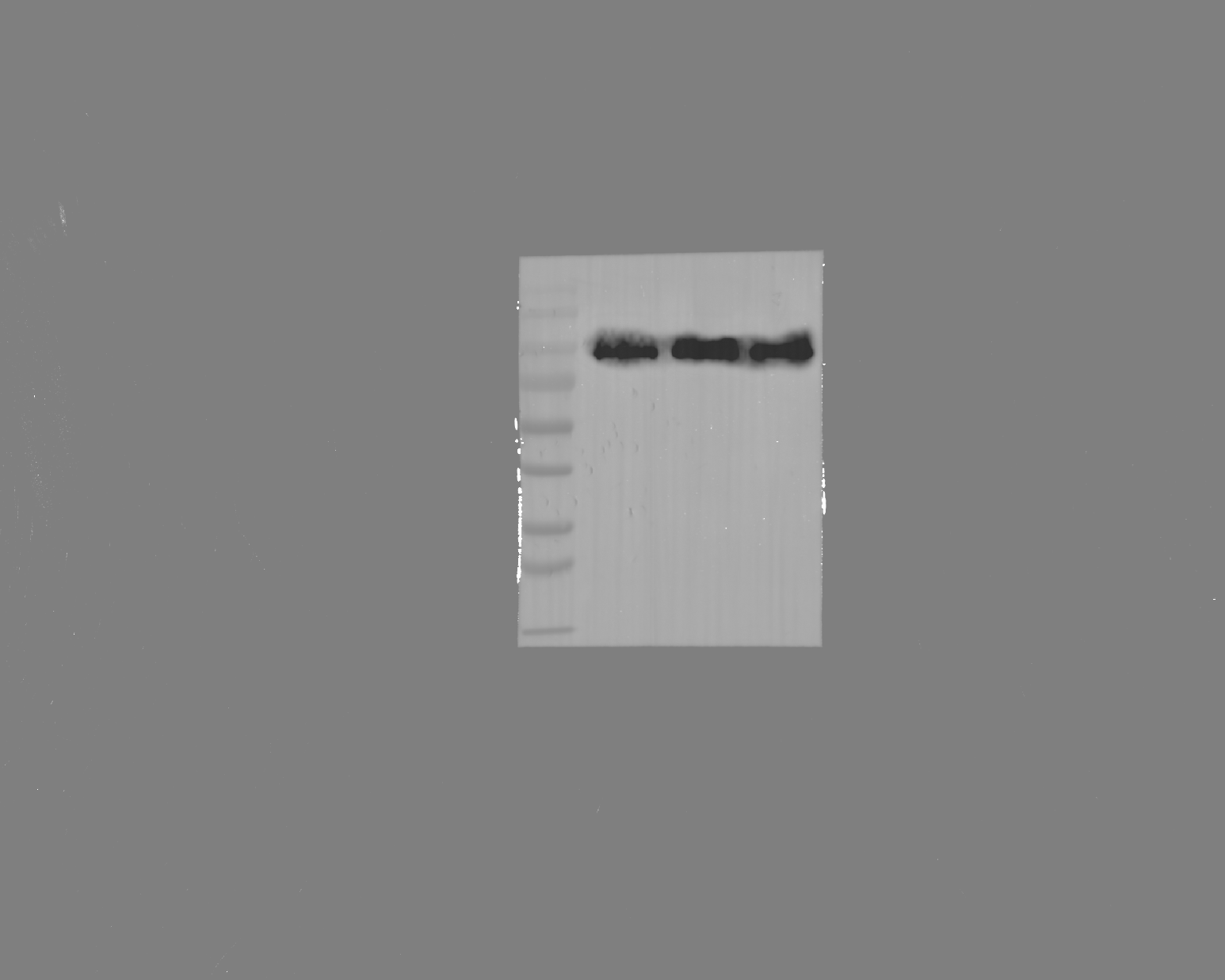

Supplement: Supplemental Information 4 [file peerj-12-18324-s004.zip › pstat3+stat3 1_1(Composite).tif]

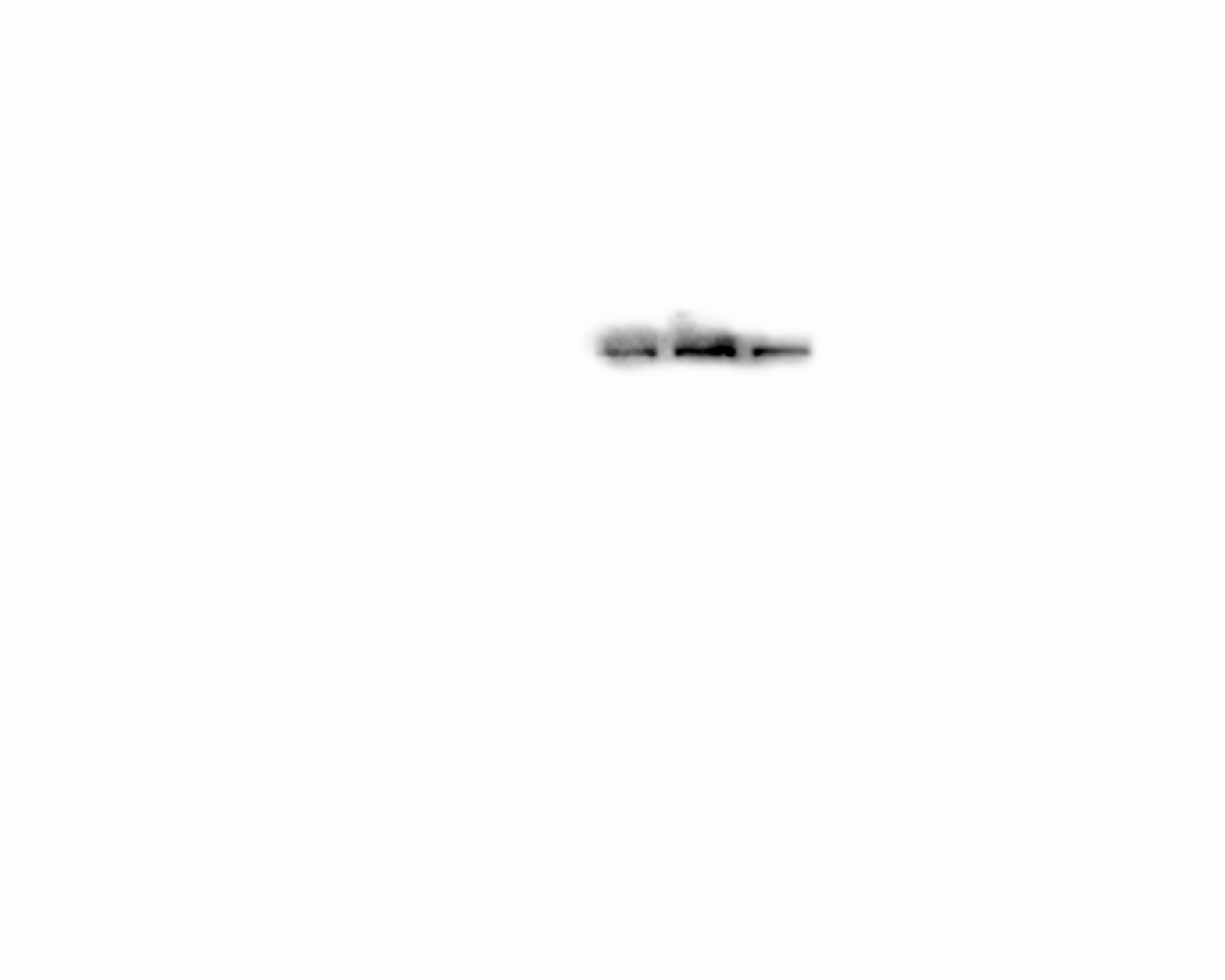

Supplement: Supplemental Information 4 [file peerj-12-18324-s004.zip › pstat3+stat3 1_2(Chemiluminescence).tif]

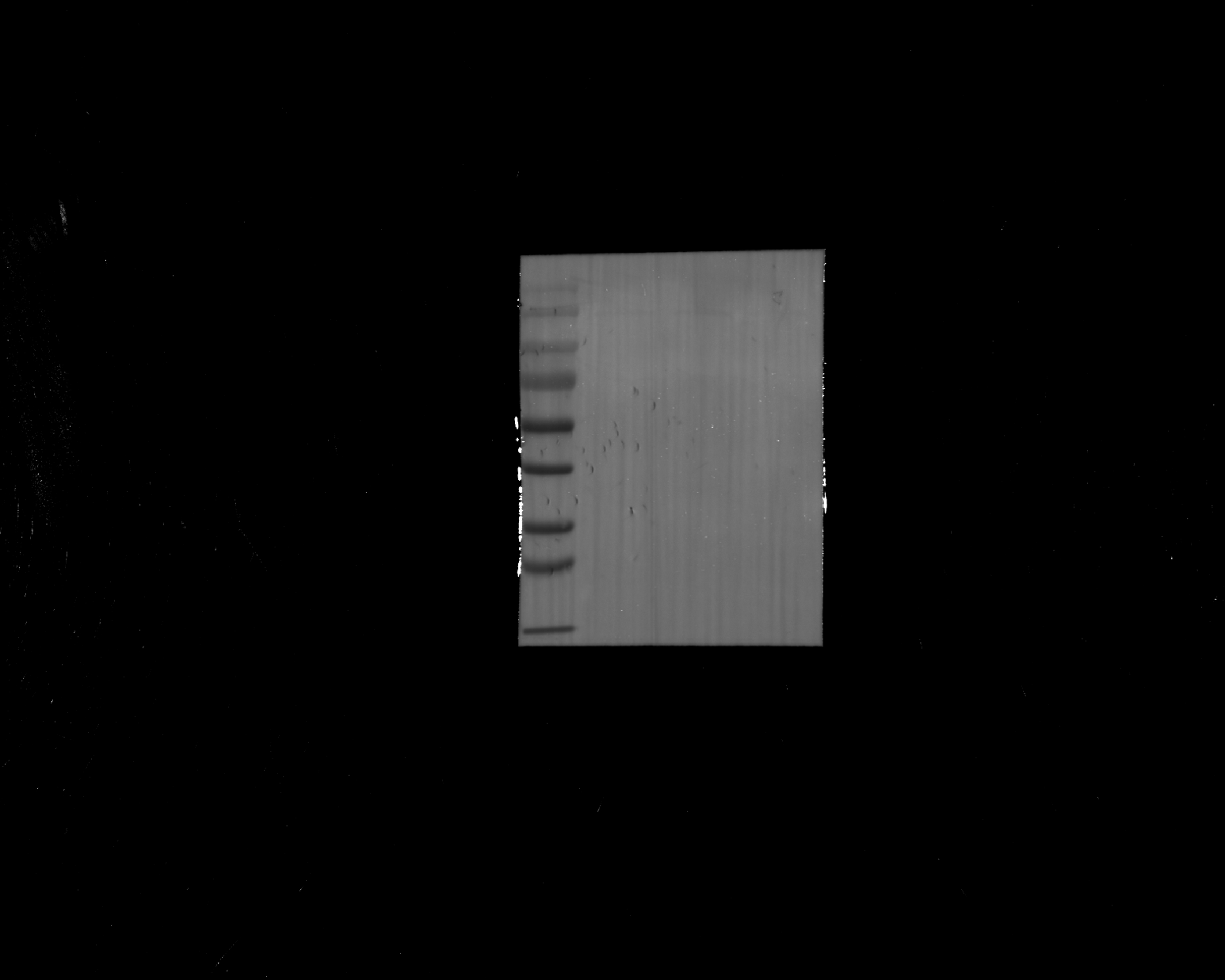

Supplement: Supplemental Information 4 [file peerj-12-18324-s004.zip › pstat3+stat3 1_2(Colorimetric).tif]

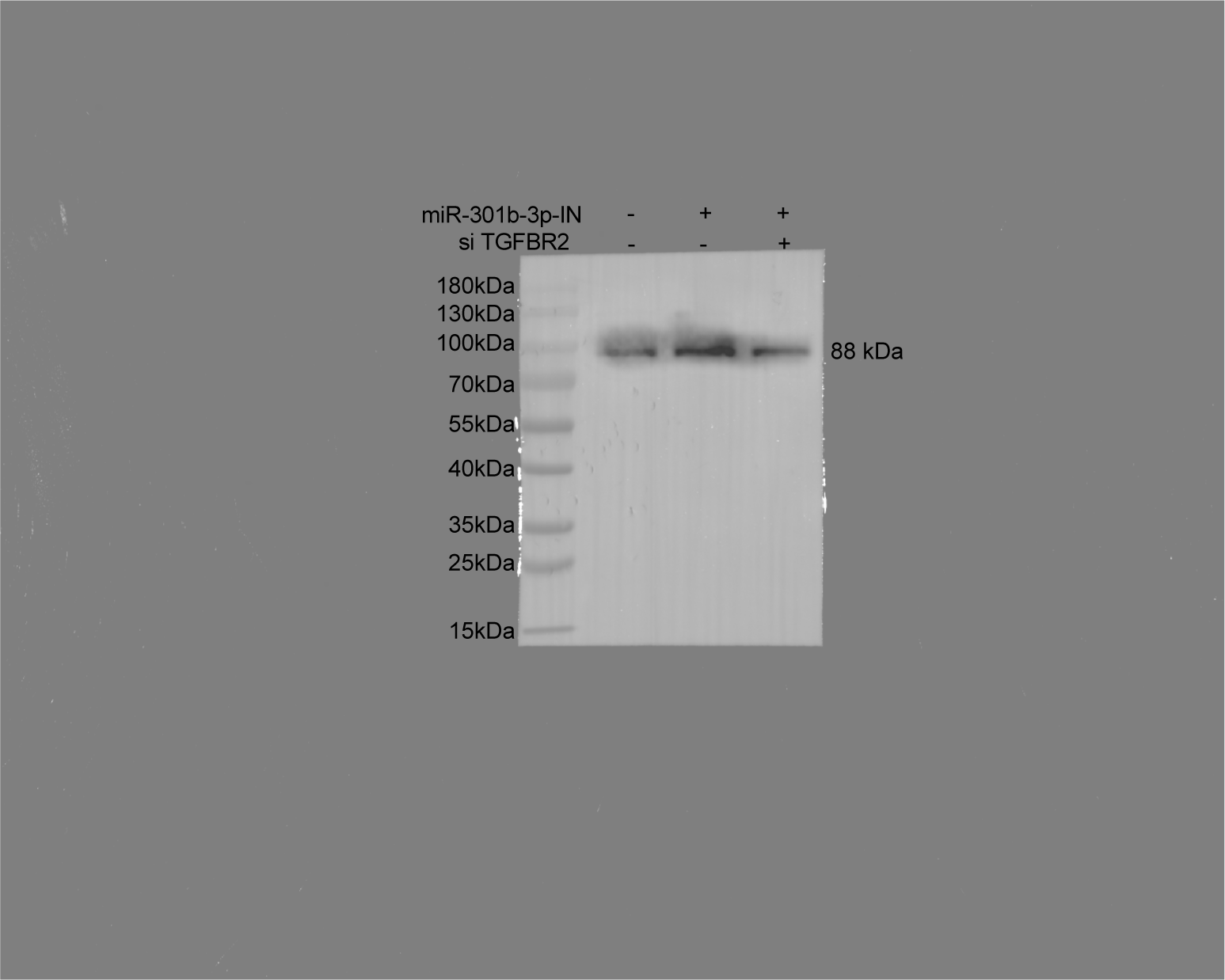

Supplement: Supplemental Information 4 [file peerj-12-18324-s004.zip › pstat3+stat3 1_2(Composite)-01.tif]

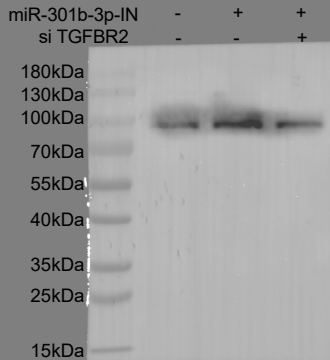

88 kDa

Supplement: Supplemental Information 4 [file peerj-12-18324-s004.zip › pstat3+stat3 1_2(Composite).pdf]

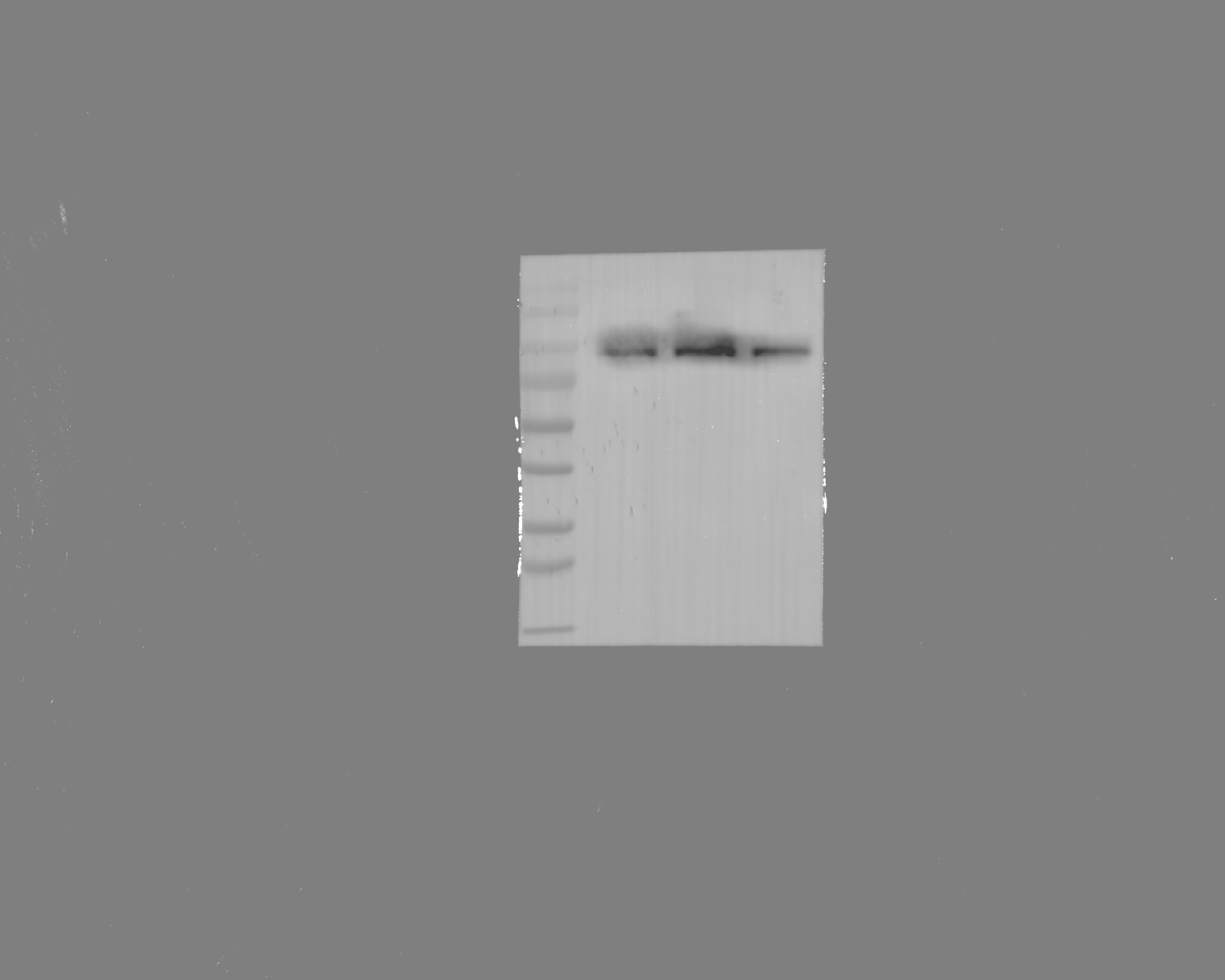

Supplement: Supplemental Information 4 [file peerj-12-18324-s004.zip › pstat3+stat3 1_2(Composite).tif]

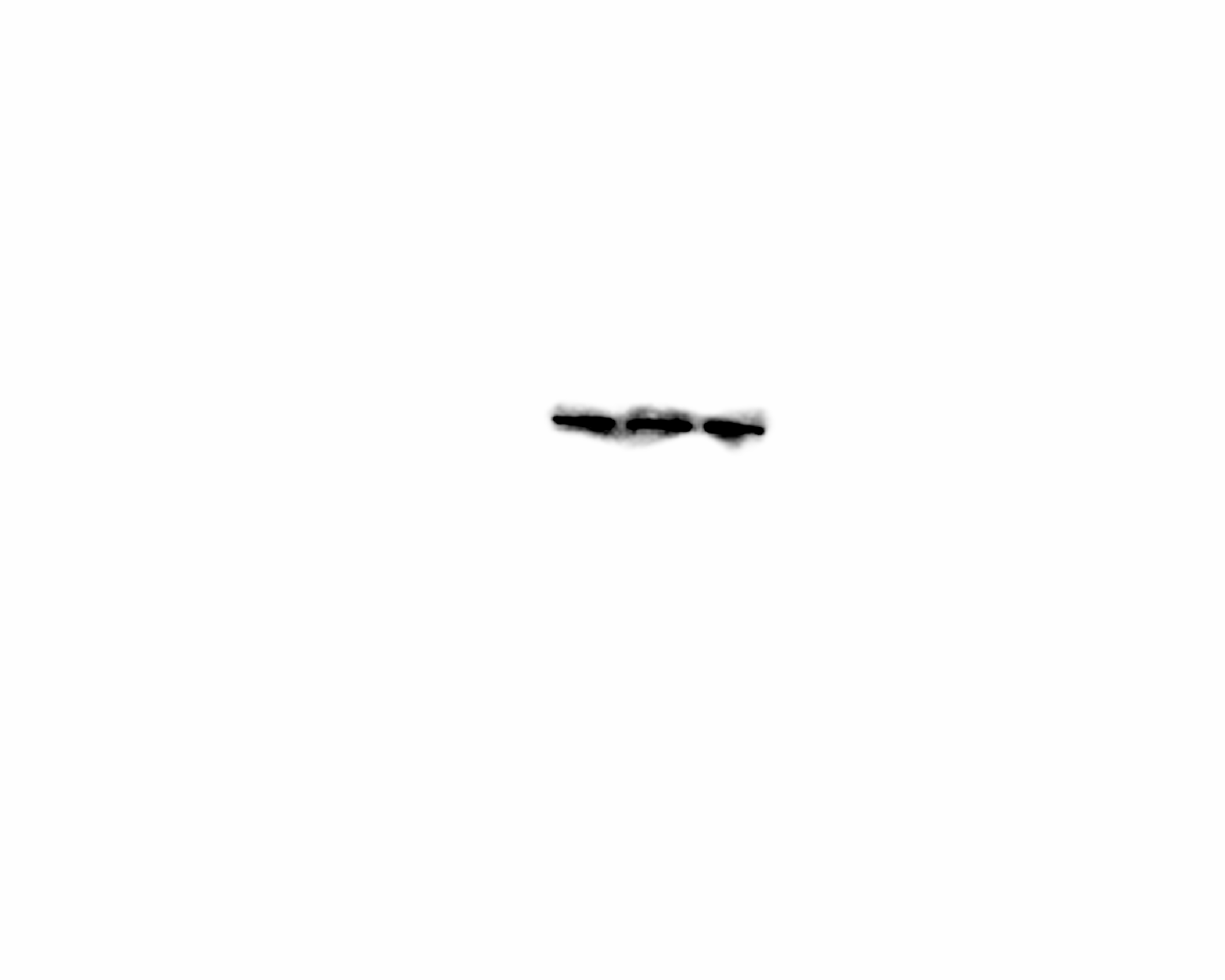

Supplement: Supplemental Information 4 [file peerj-12-18324-s004.zip › pstat3+stat3 2_1(Chemiluminescence).tif]

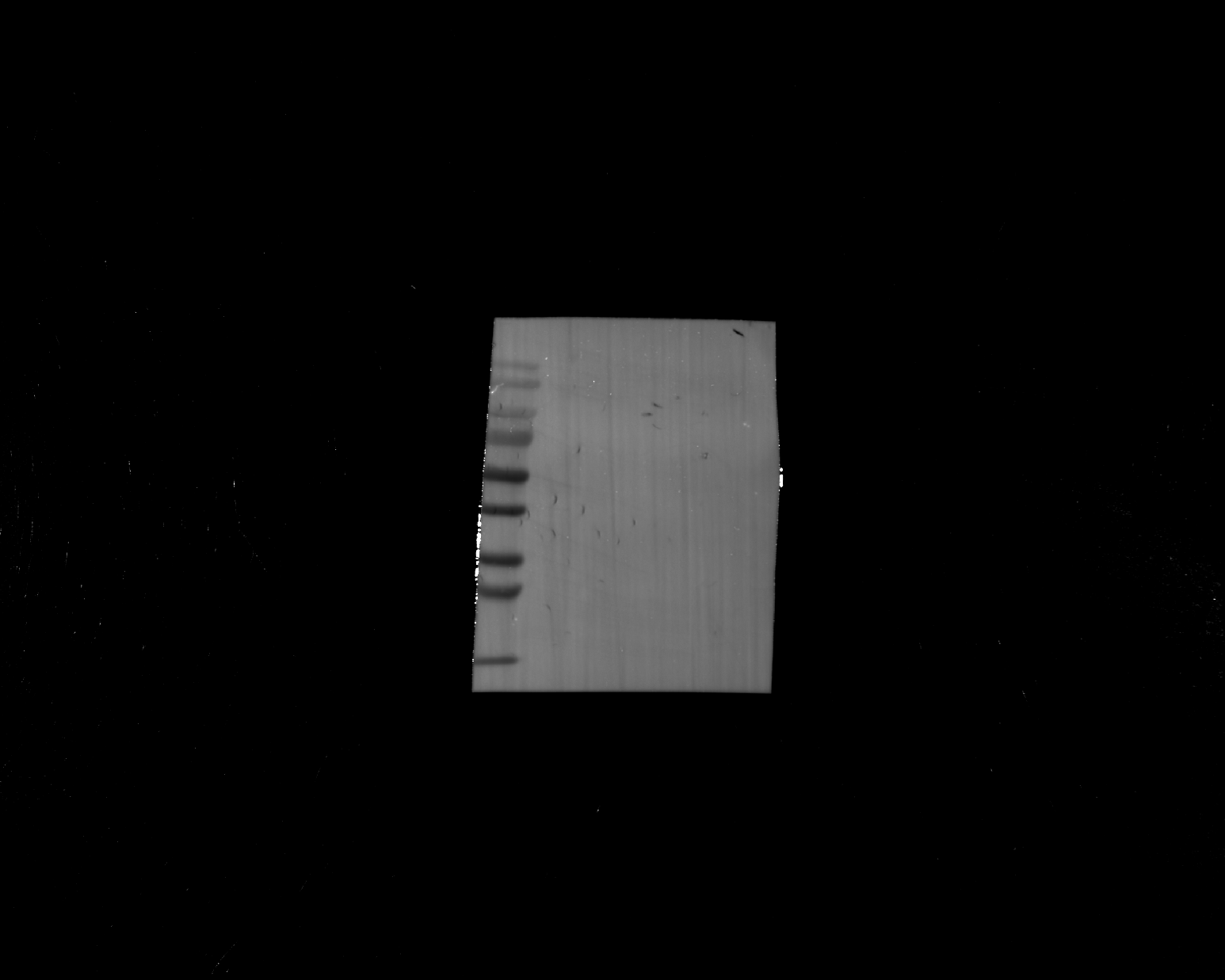

Supplement: Supplemental Information 4 [file peerj-12-18324-s004.zip › pstat3+stat3 2_1(Colorimetric).tif]

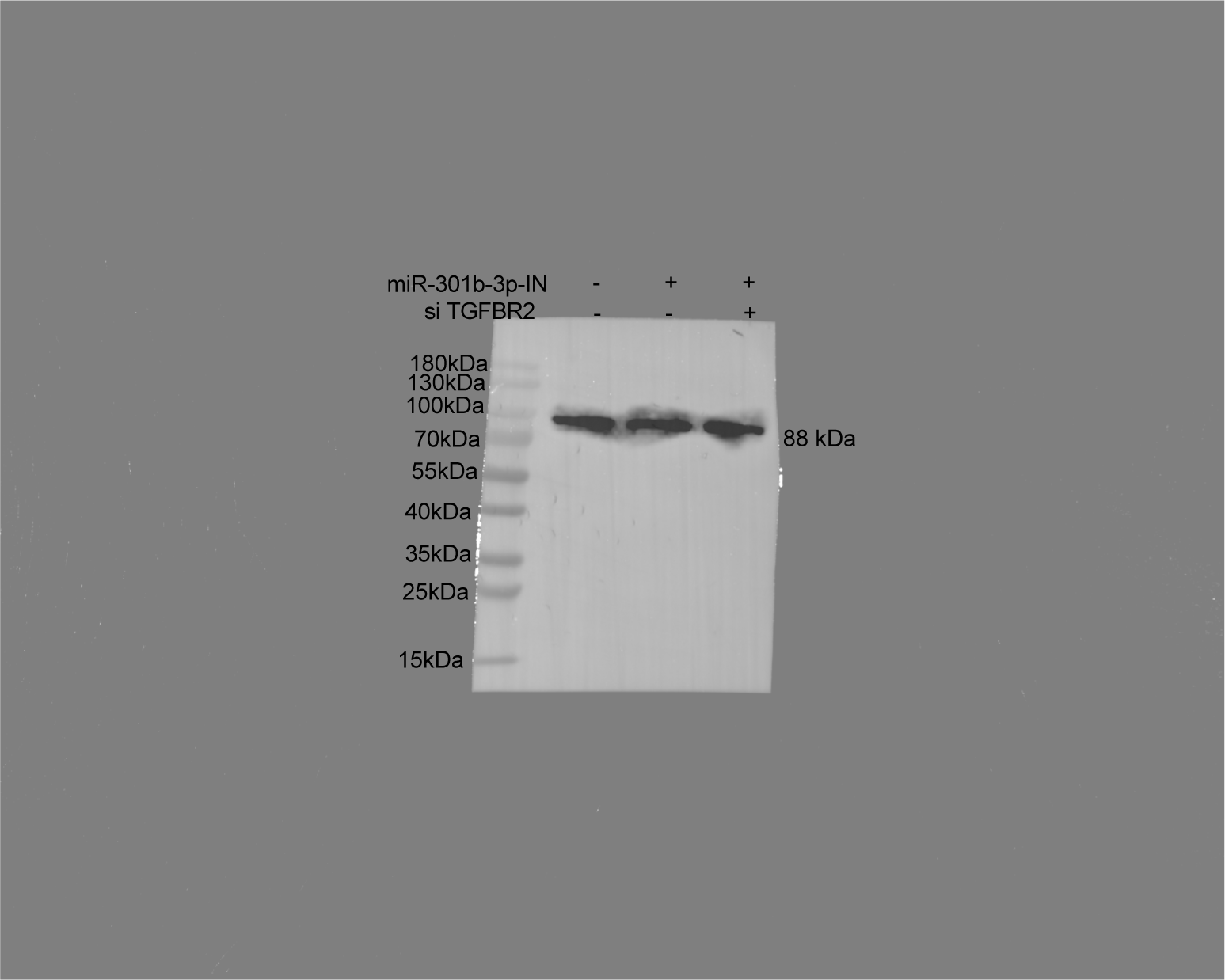

Supplement: Supplemental Information 4 [file peerj-12-18324-s004.zip › pstat3+stat3 2_1(Composite)-01.tif]

|                |   |   |   |
|----------------|---|---|---|
| miR-301b-3p-IN | - | + | + |
| si TGFR2       | - | - | + |

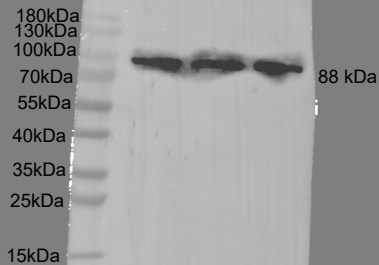

Supplement: Supplemental Information 4 [file peerj-12-18324-s004.zip › pstat3+stat3 2_1(Composite).pdf]

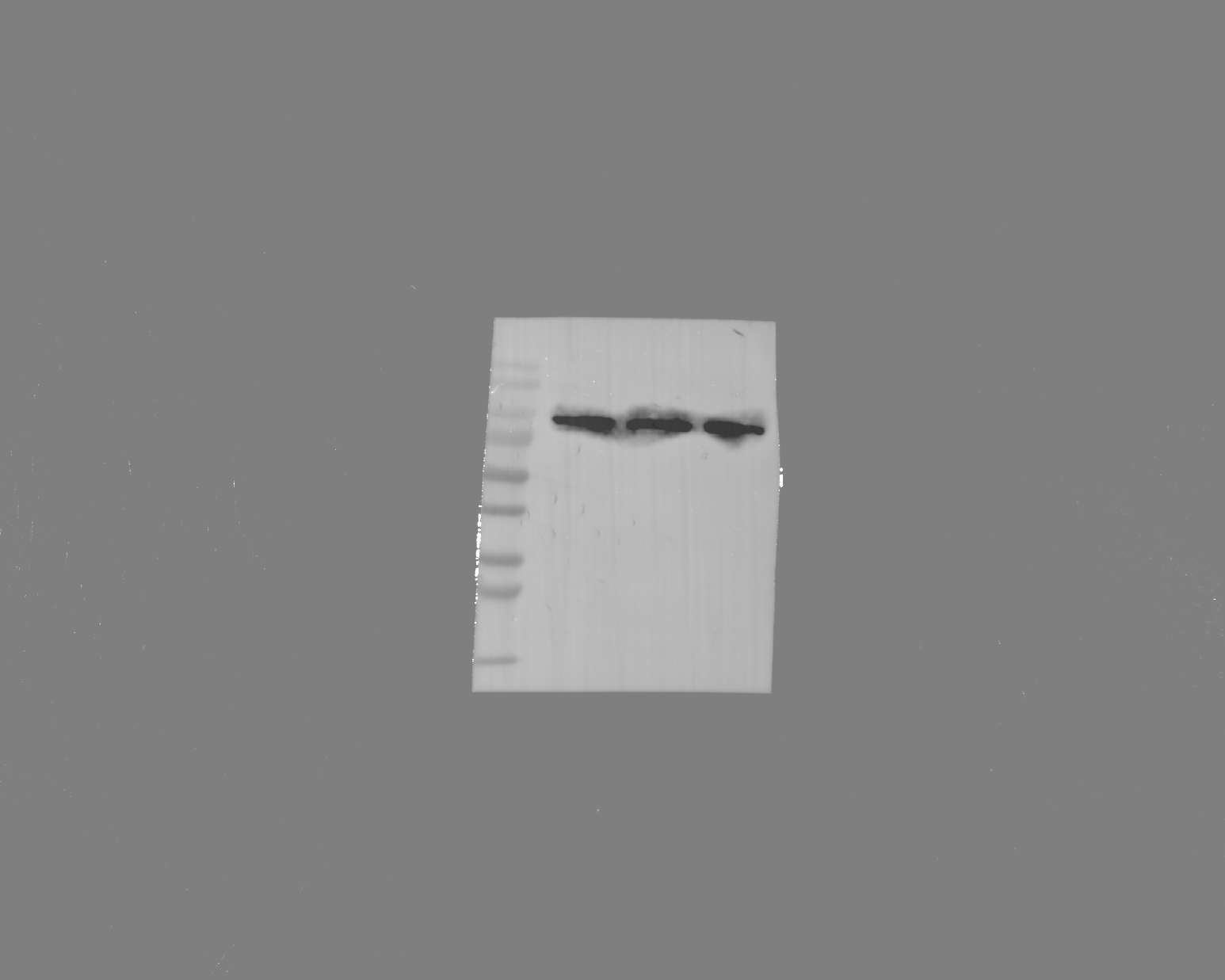

Supplement: Supplemental Information 4 [file peerj-12-18324-s004.zip › pstat3+stat3 2_1(Composite).tif]

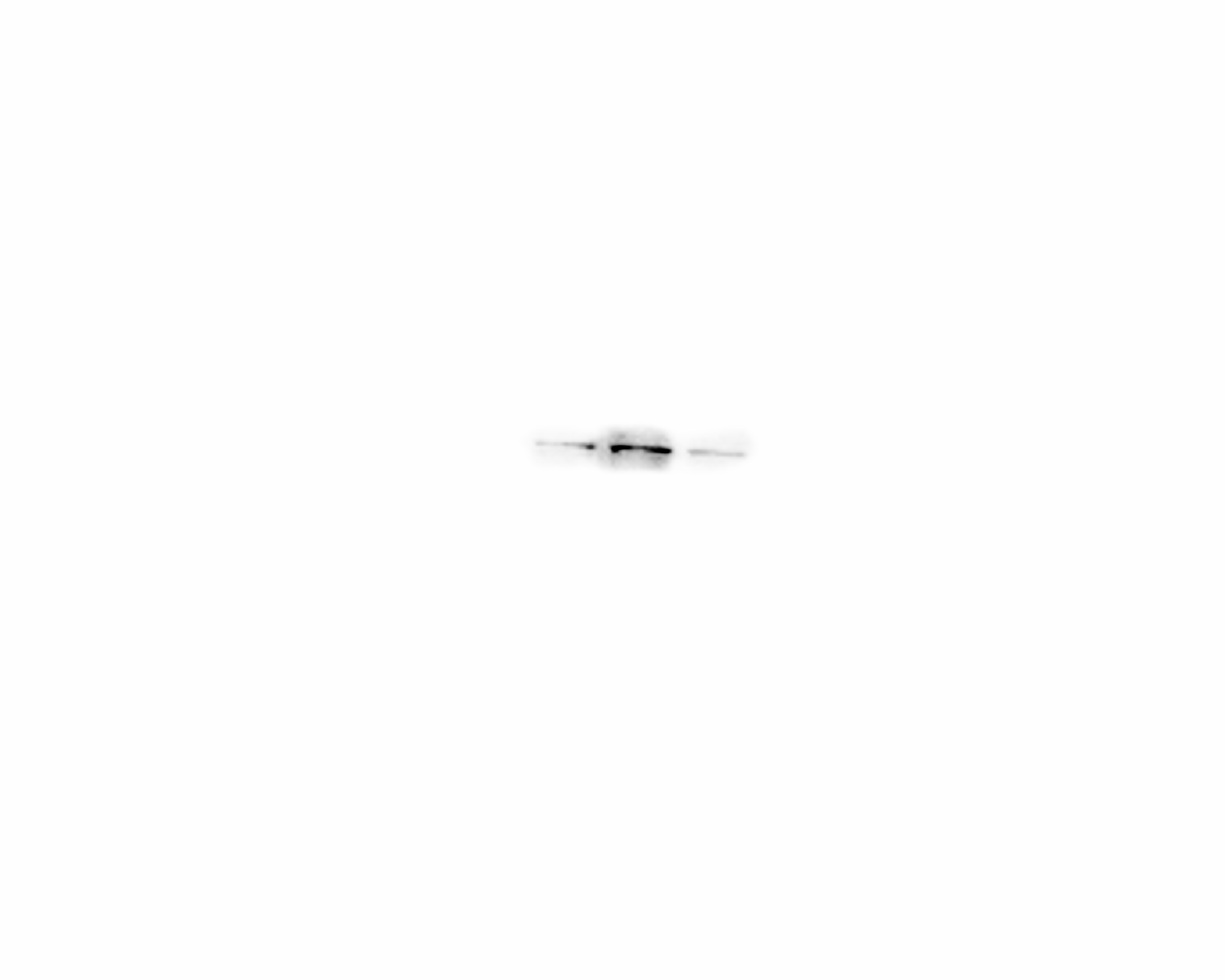

Supplement: Supplemental Information 4 [file peerj-12-18324-s004.zip › pstat3+stat3 2_2(Chemiluminescence).tif]

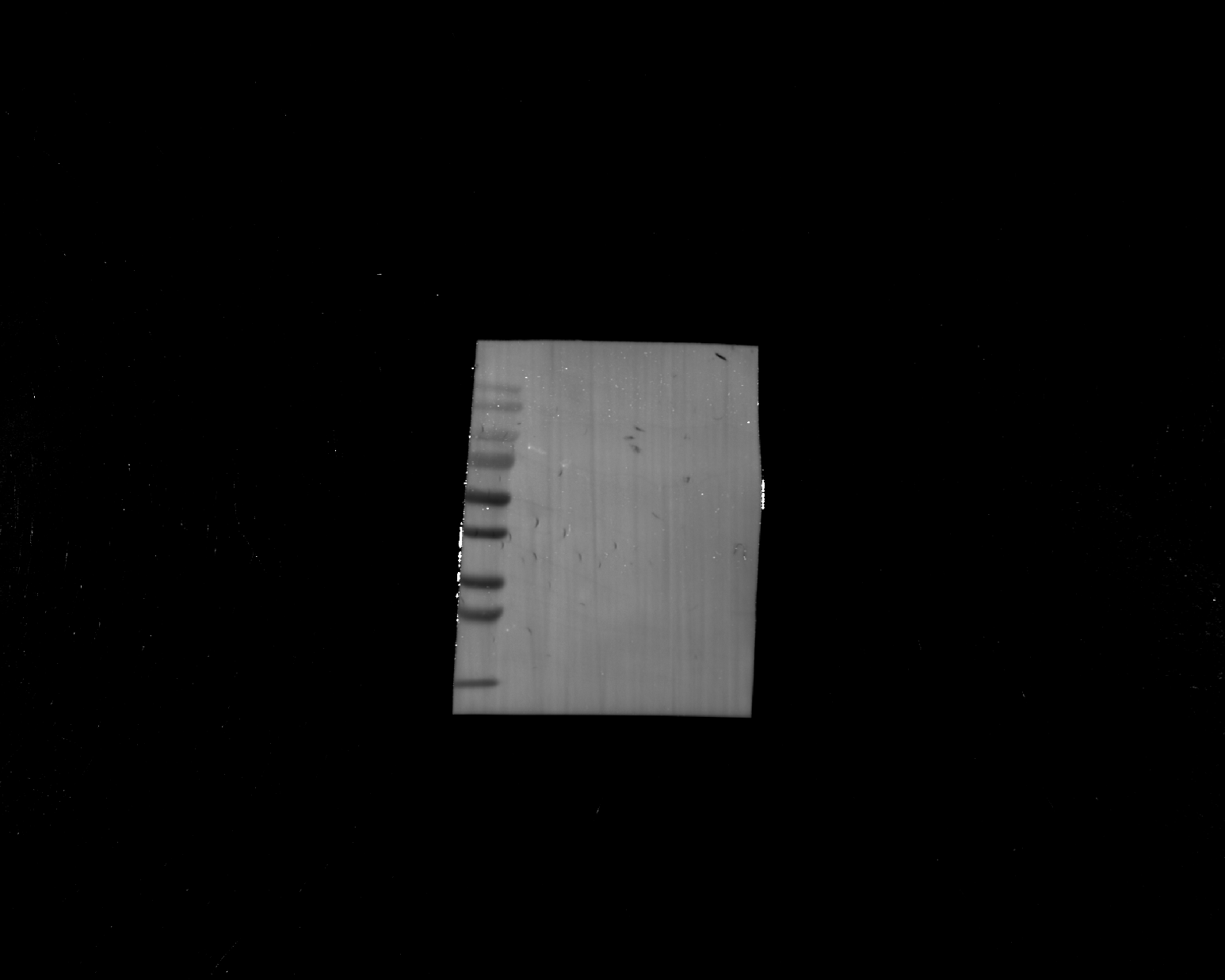

Supplement: Supplemental Information 4 [file peerj-12-18324-s004.zip › pstat3+stat3 2_2(Colorimetric).tif]

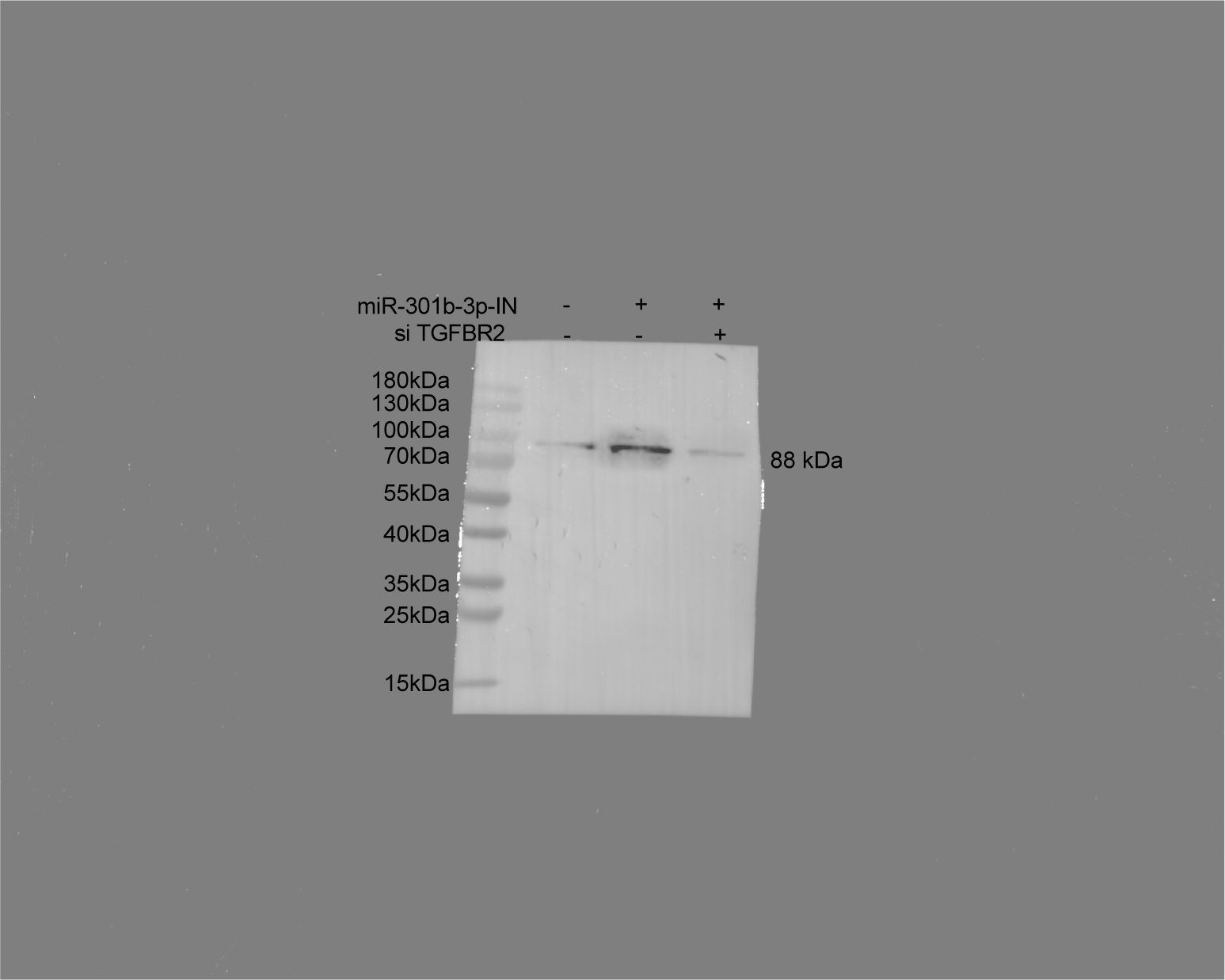

Supplement: Supplemental Information 4 [file peerj-12-18324-s004.zip › pstat3+stat3 2_2(Composite)-01.tif]

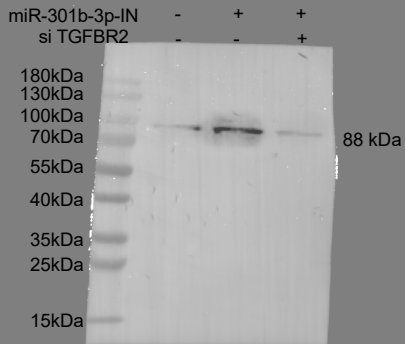

Supplement: Supplemental Information 4 [file peerj-12-18324-s004.zip › pstat3+stat3 2_2(Composite).pdf]

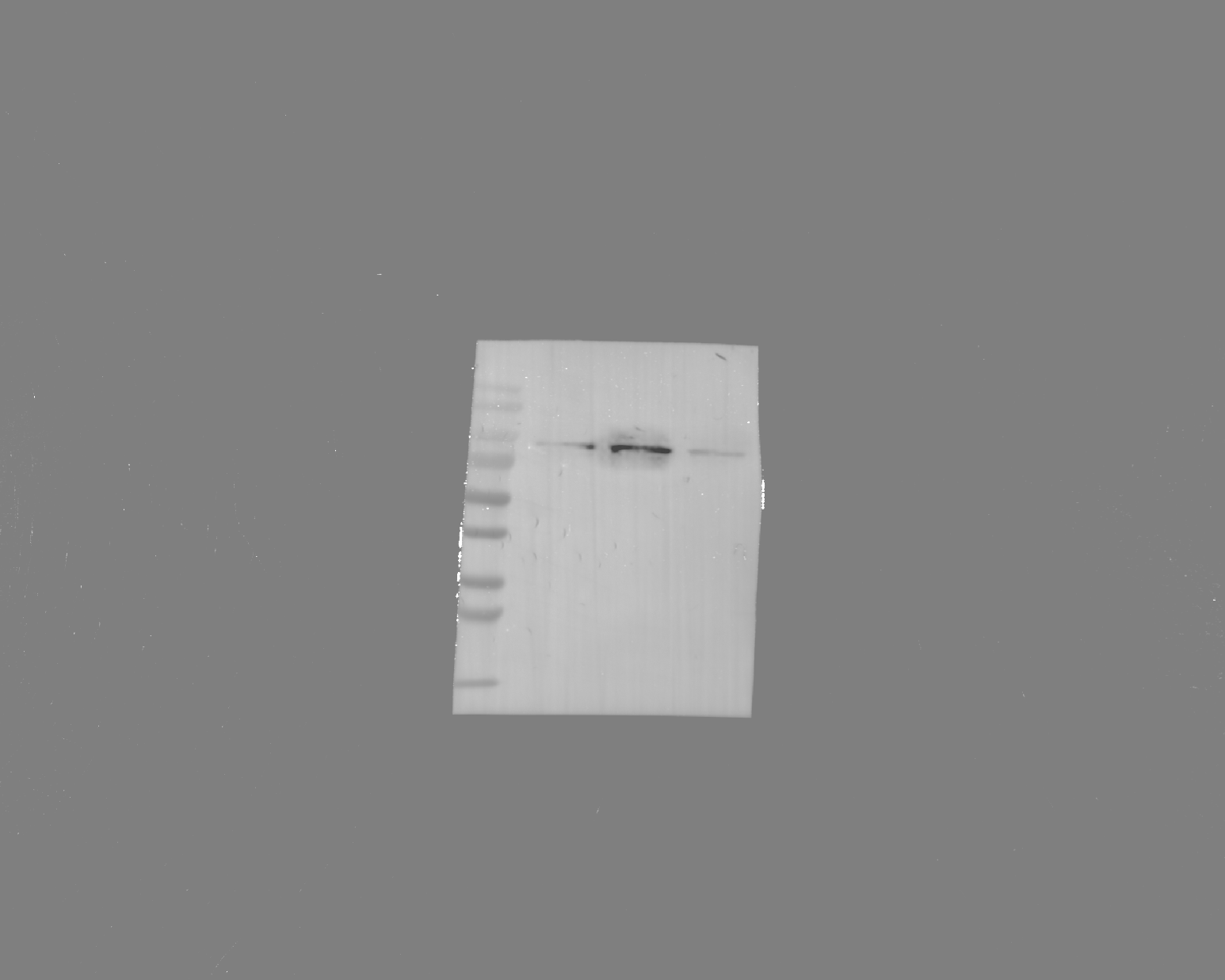

Supplement: Supplemental Information 4 [file peerj-12-18324-s004.zip › pstat3+stat3 2_2(Composite).tif]

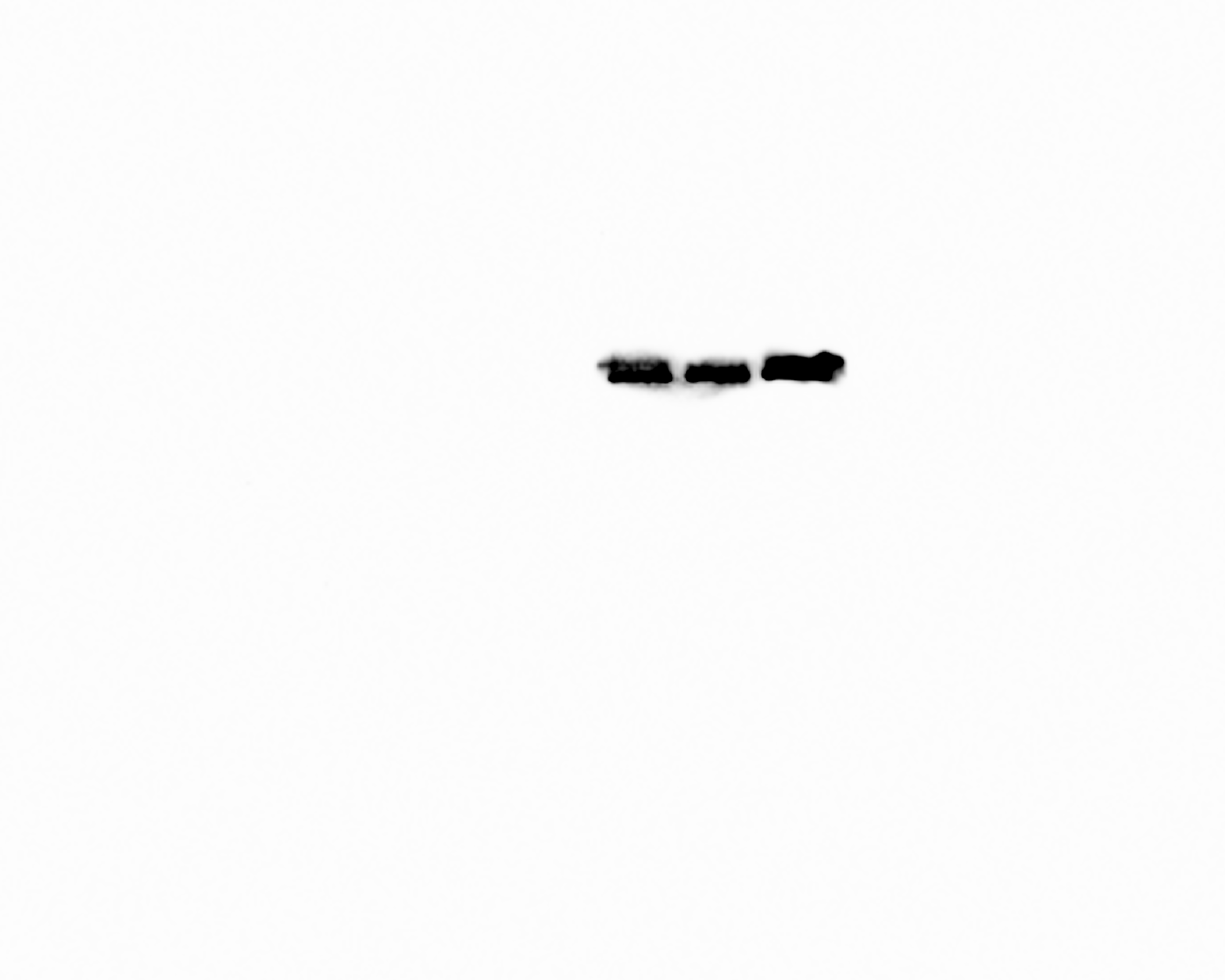

Supplement: Supplemental Information 4 [file peerj-12-18324-s004.zip › pstat3+stst3 3_1(Chemiluminescence).tif]

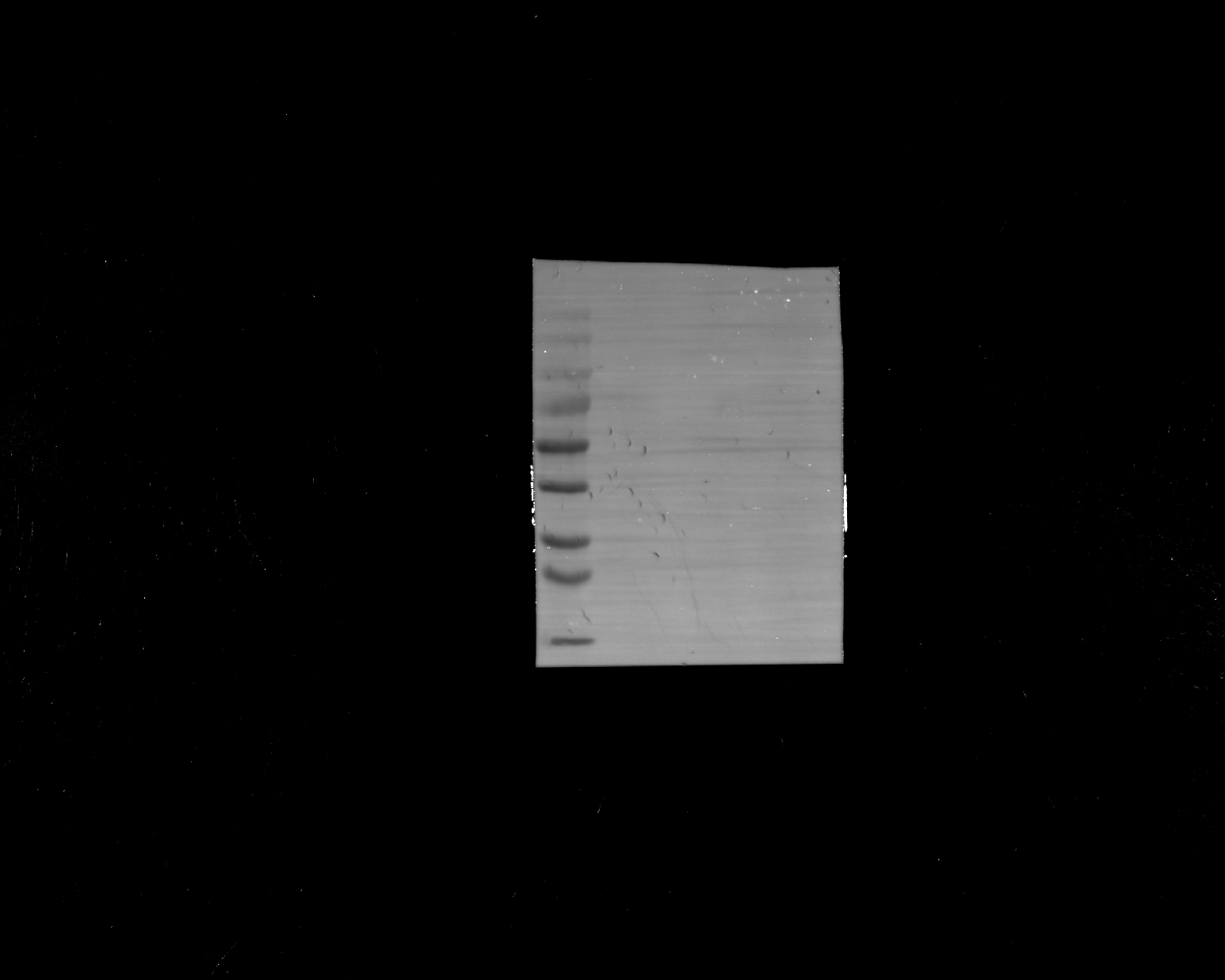

Supplement: Supplemental Information 4 [file peerj-12-18324-s004.zip › pstat3+stst3 3_1(Colorimetric).tif]

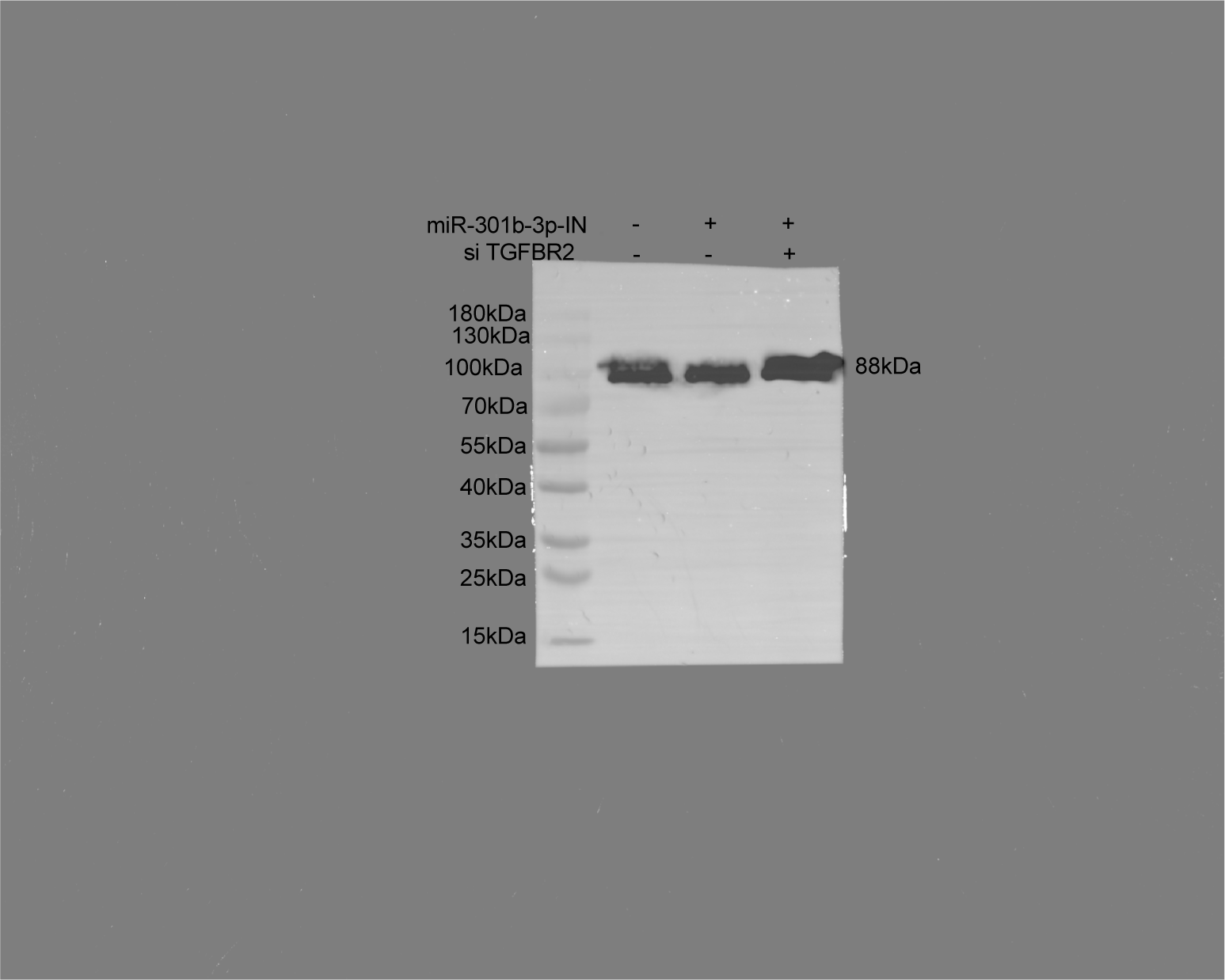

Supplement: Supplemental Information 4 [file peerj-12-18324-s004.zip › pstat3+stst3 3_1(Composite)-01.tif]

|                |   |   |   |
|----------------|---|---|---|
| miR-301b-3p-IN | - | + | + |
| si TGFBR2      | - | - | + |

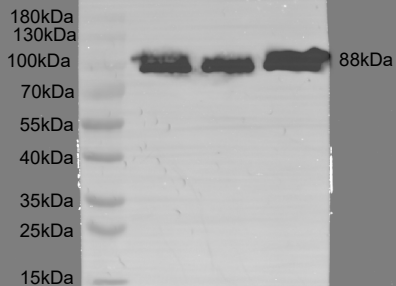

Supplement: Supplemental Information 4 [file peerj-12-18324-s004.zip › pstat3+stst3 3_1(Composite).pdf]

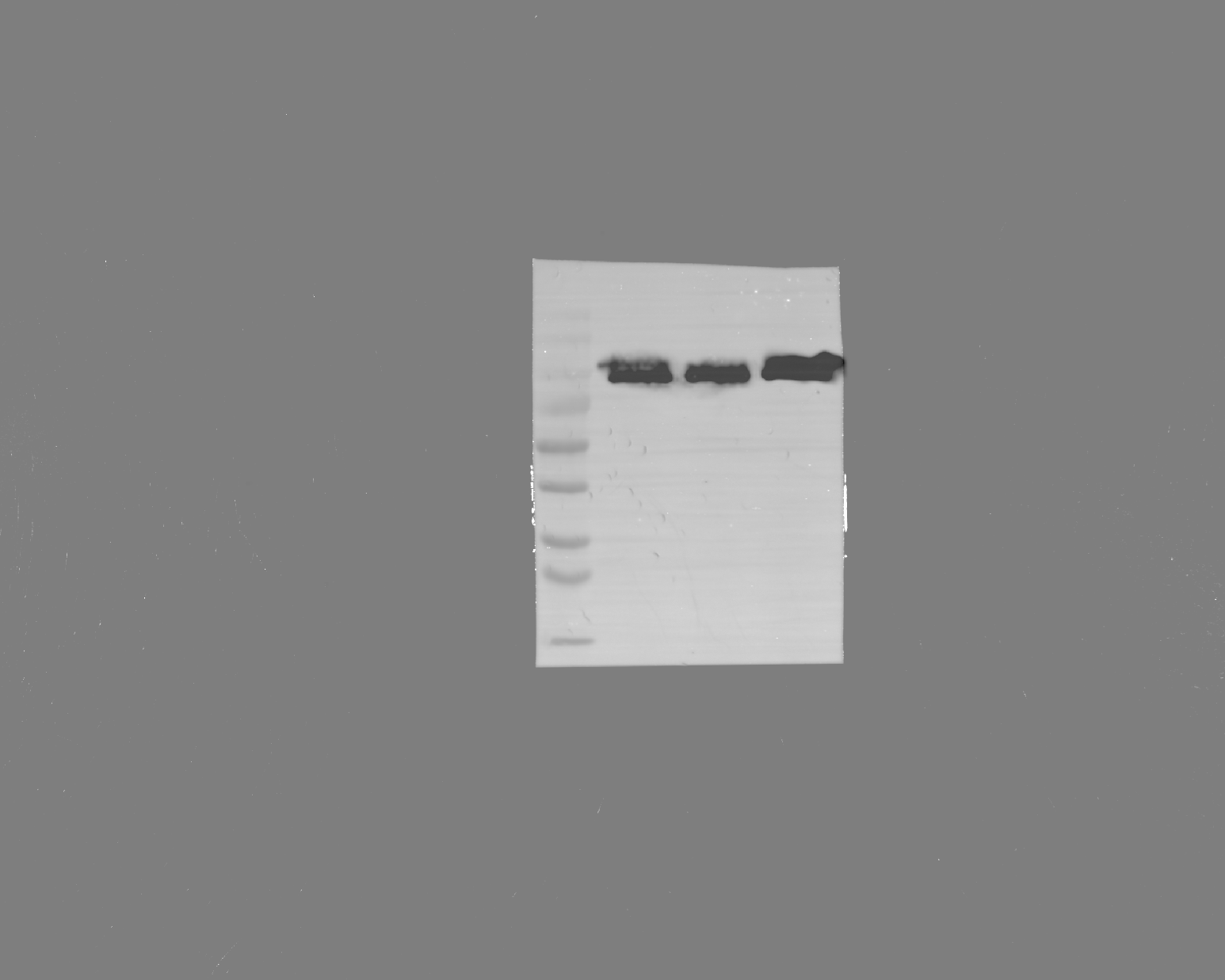

Supplement: Supplemental Information 4 [file peerj-12-18324-s004.zip › pstat3+stst3 3_1(Composite).tif]

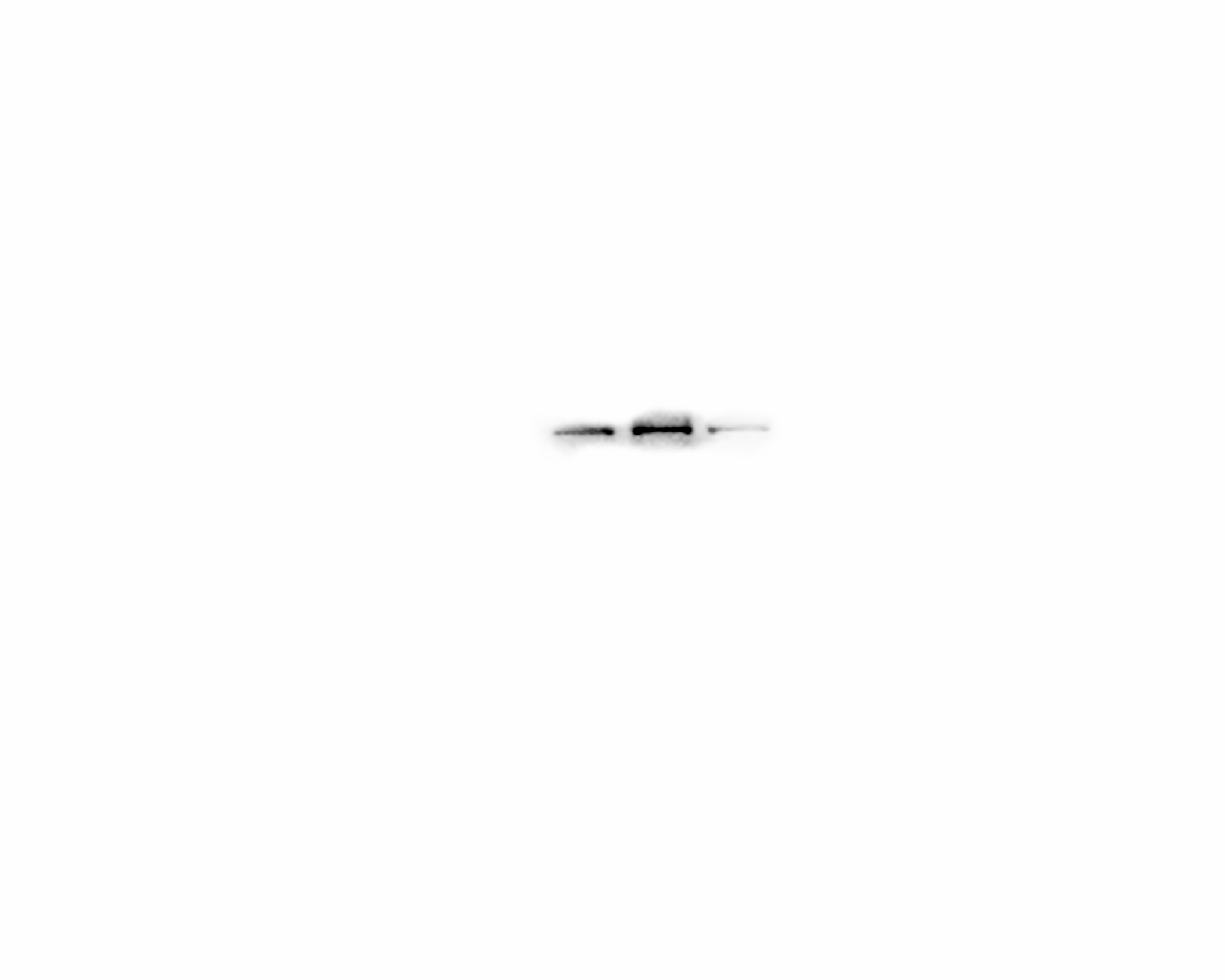

Supplement: Supplemental Information 4 [file peerj-12-18324-s004.zip › pstat3+stst3 3_2(Chemiluminescence).tif]

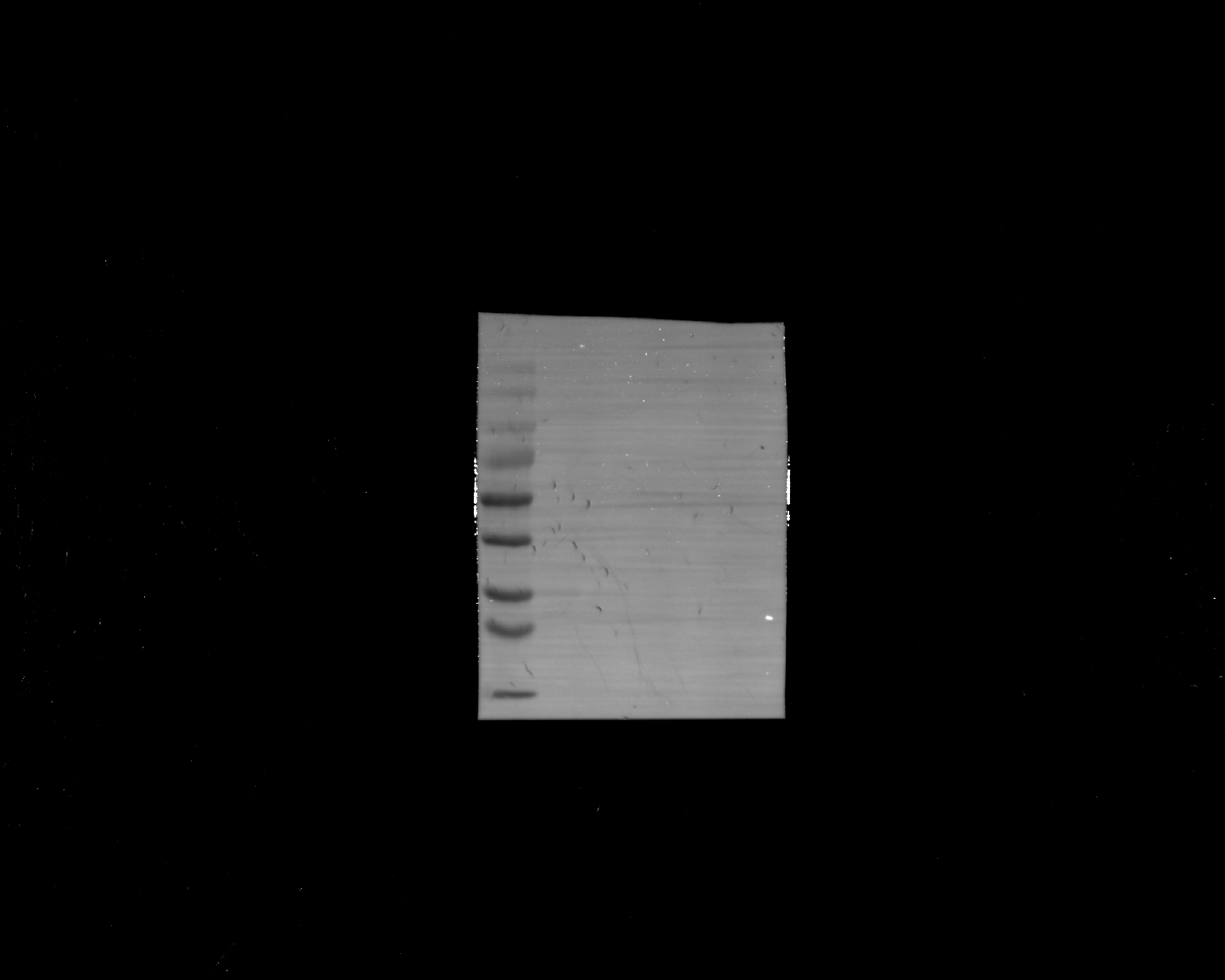

Supplement: Supplemental Information 4 [file peerj-12-18324-s004.zip › pstat3+stst3 3_2(Colorimetric).tif]

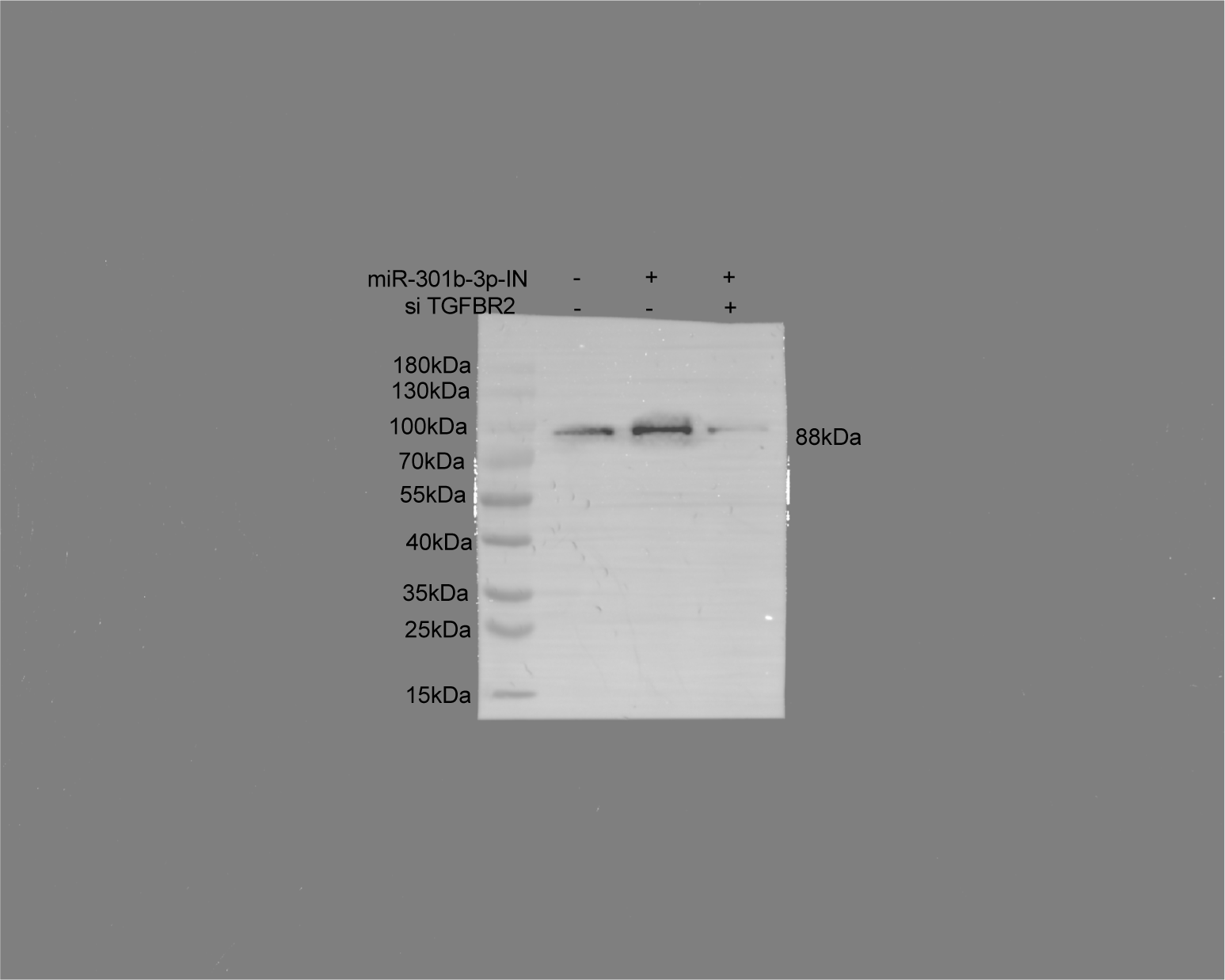

Supplement: Supplemental Information 4 [file peerj-12-18324-s004.zip › pstat3+stst3 3_2(Composite)-01.tif]

|                |   |   |   |
|----------------|---|---|---|
| miR-301b-3p-IN | - | + | + |
| si TGFBR2      | - | - | + |

180kDa  
130kDa  
100kDa  
70kDa  
55kDa  
40kDa  
35kDa  
25kDa  
15kDa

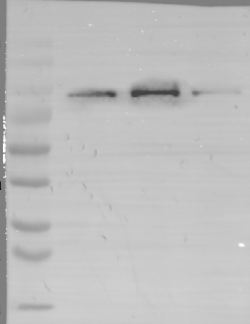

88kDa

Supplement: Supplemental Information 4 [file peerj-12-18324-s004.zip › pstat3+stst3 3_2(Composite).pdf]

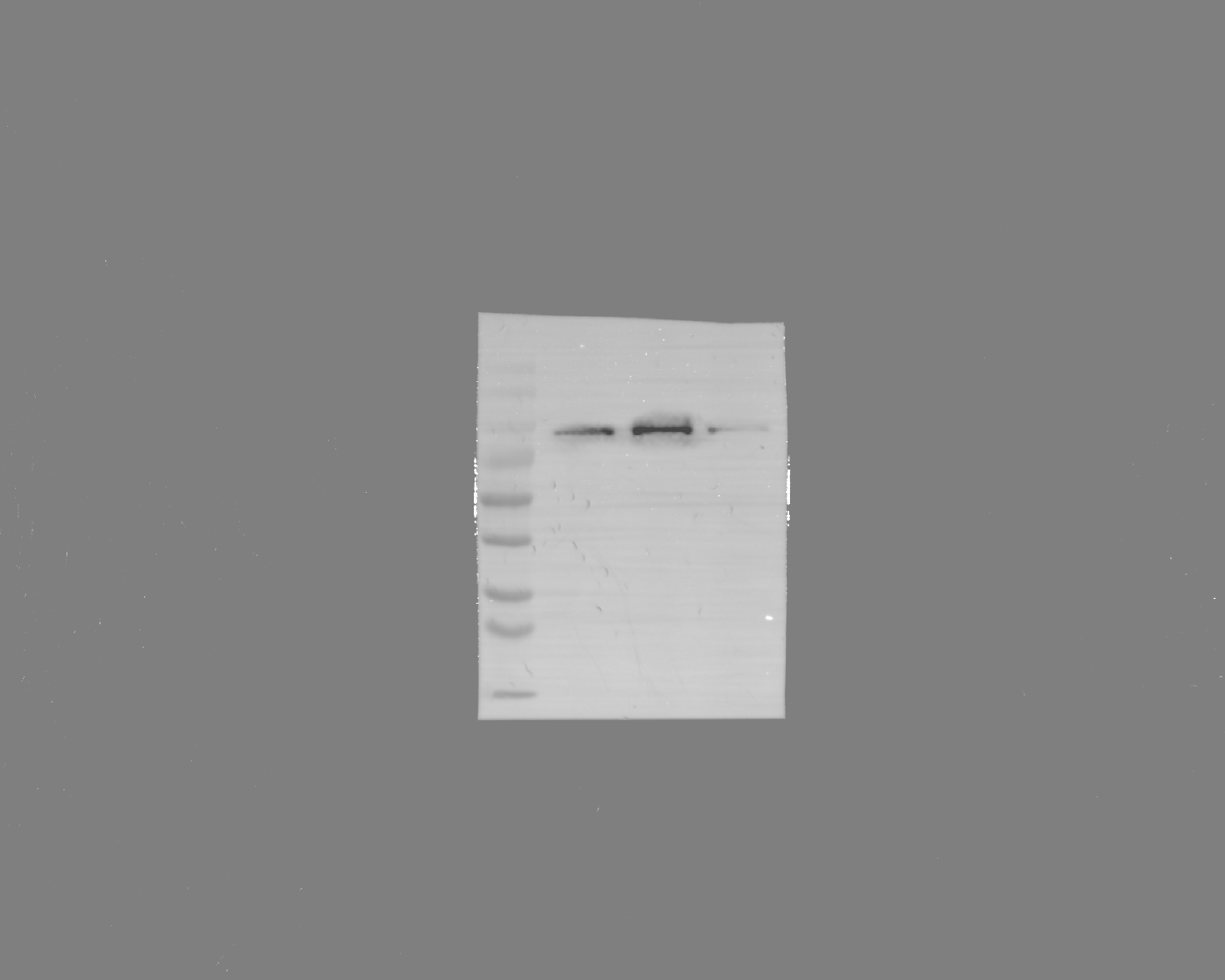

Supplement: Supplemental Information 4 [file peerj-12-18324-s004.zip › pstat3+stst3 3_2(Composite).tif]

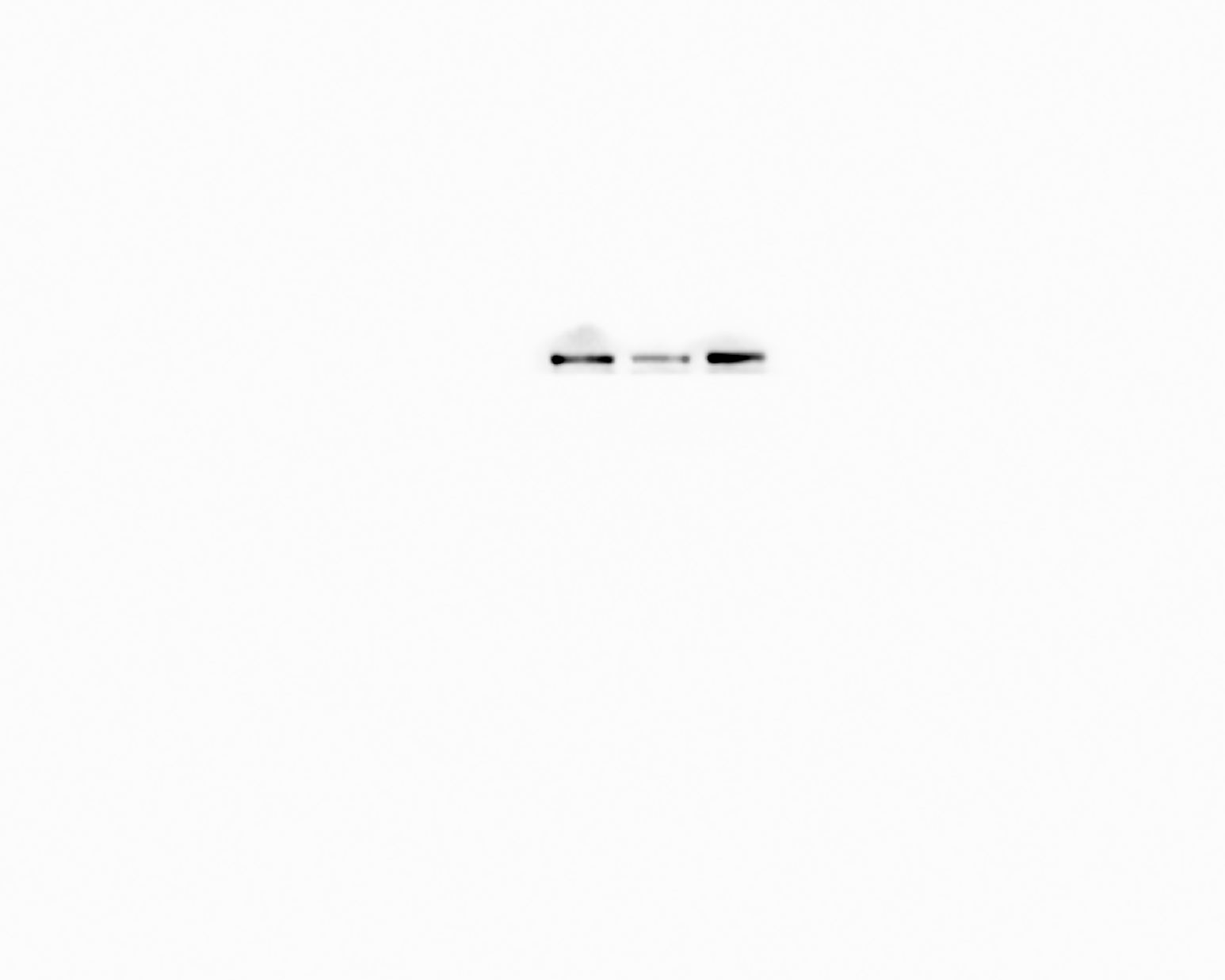

Supplement: Supplemental Information 5 [file peerj-12-18324-s005.zip › pstat6+stat6 1_1(Chemiluminescence).tif]

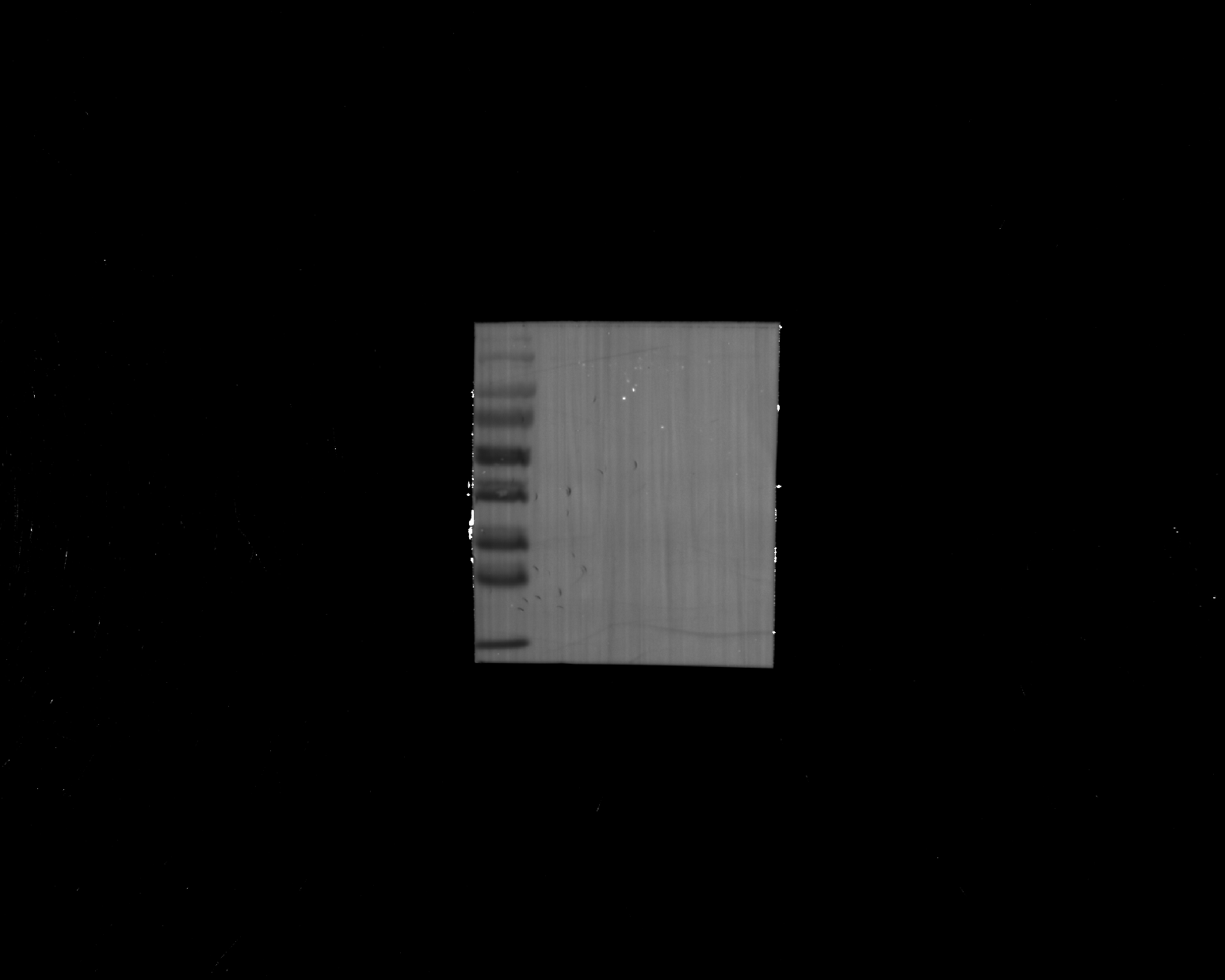

Supplement: Supplemental Information 5 [file peerj-12-18324-s005.zip › pstat6+stat6 1_1(Colorimetric).tif]

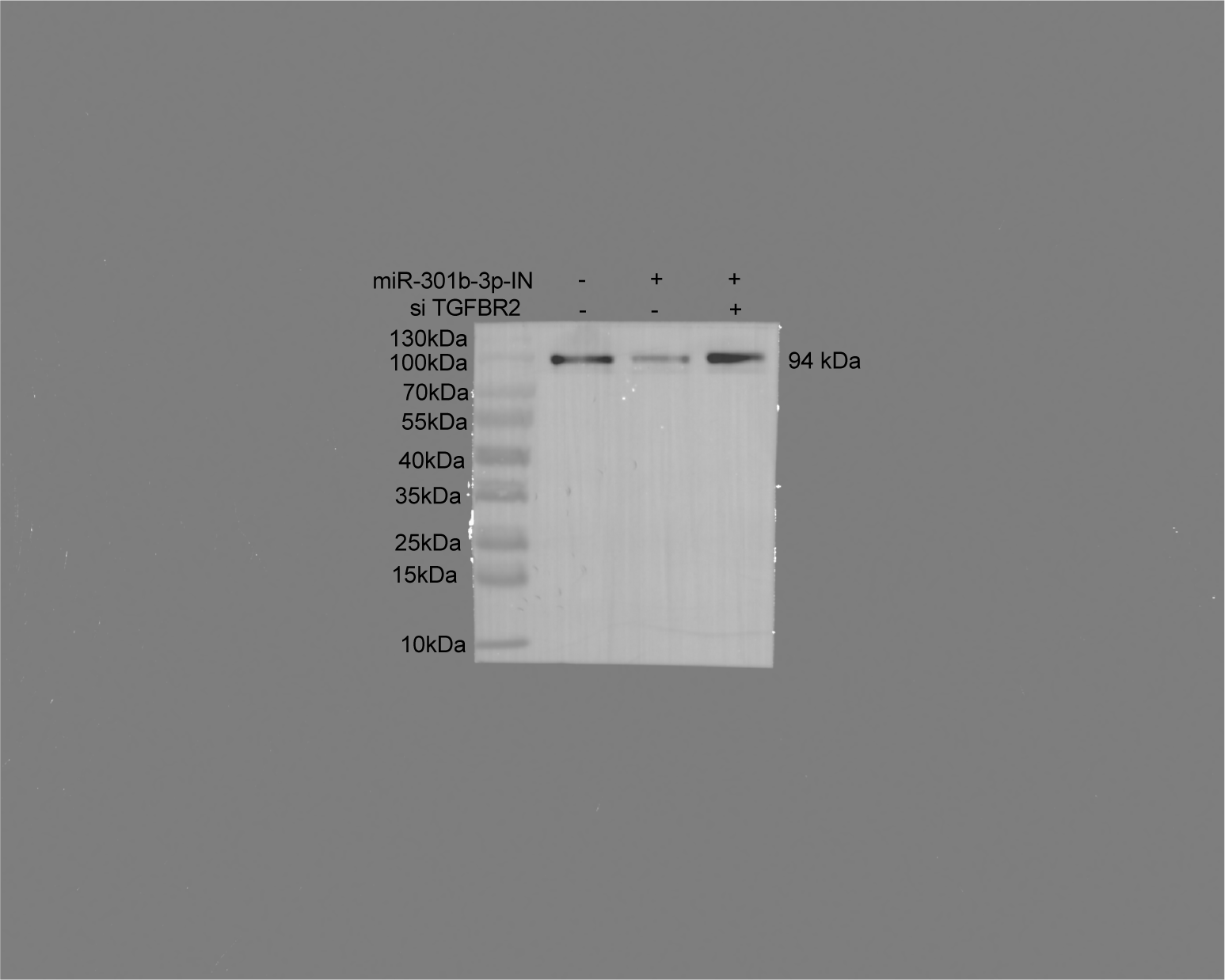

Supplement: Supplemental Information 5 [file peerj-12-18324-s005.zip › pstat6+stat6 1_1(Composite)-01.tif]

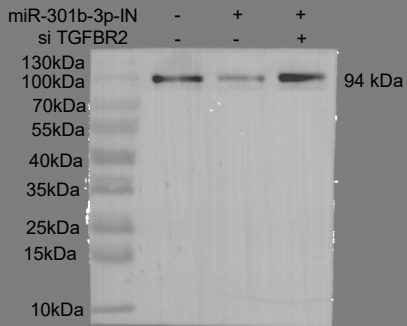

Supplement: Supplemental Information 5 [file peerj-12-18324-s005.zip › pstat6+stat6 1_1(Composite).pdf]

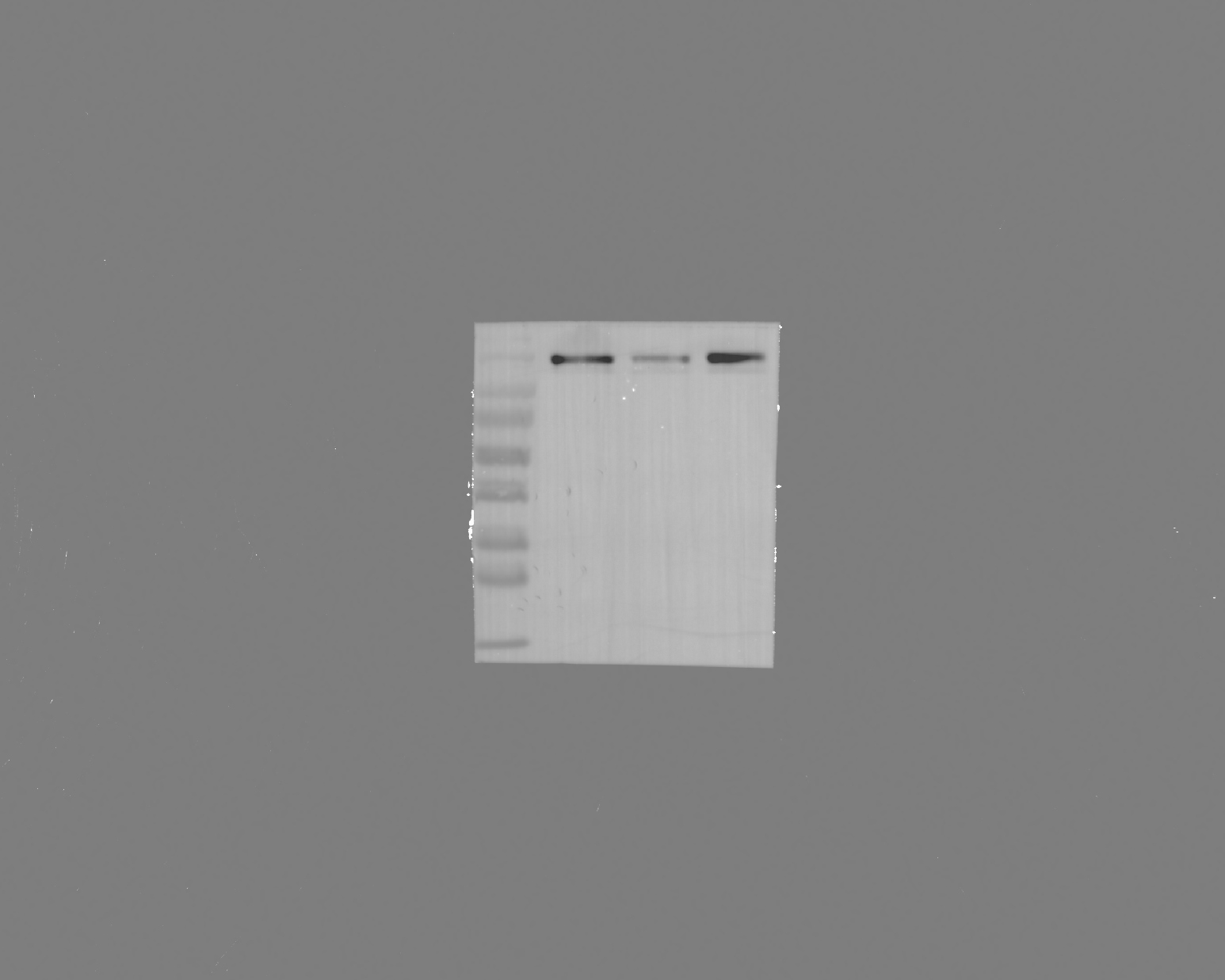

Supplement: Supplemental Information 5 [file peerj-12-18324-s005.zip › pstat6+stat6 1_1(Composite).tif]

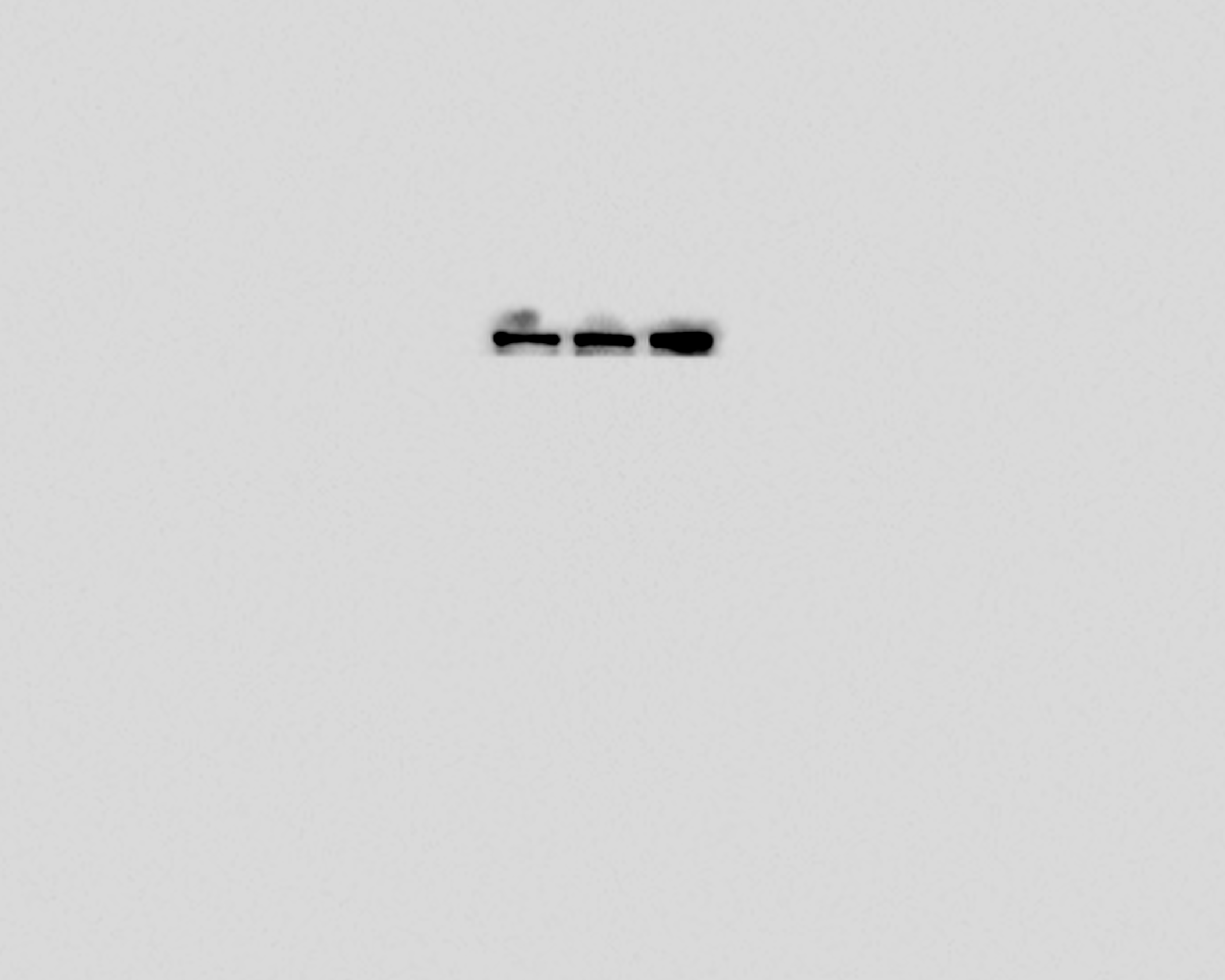

Supplement: Supplemental Information 5 [file peerj-12-18324-s005.zip › pstat6+stat6 1_2(Chemiluminescence).tif]

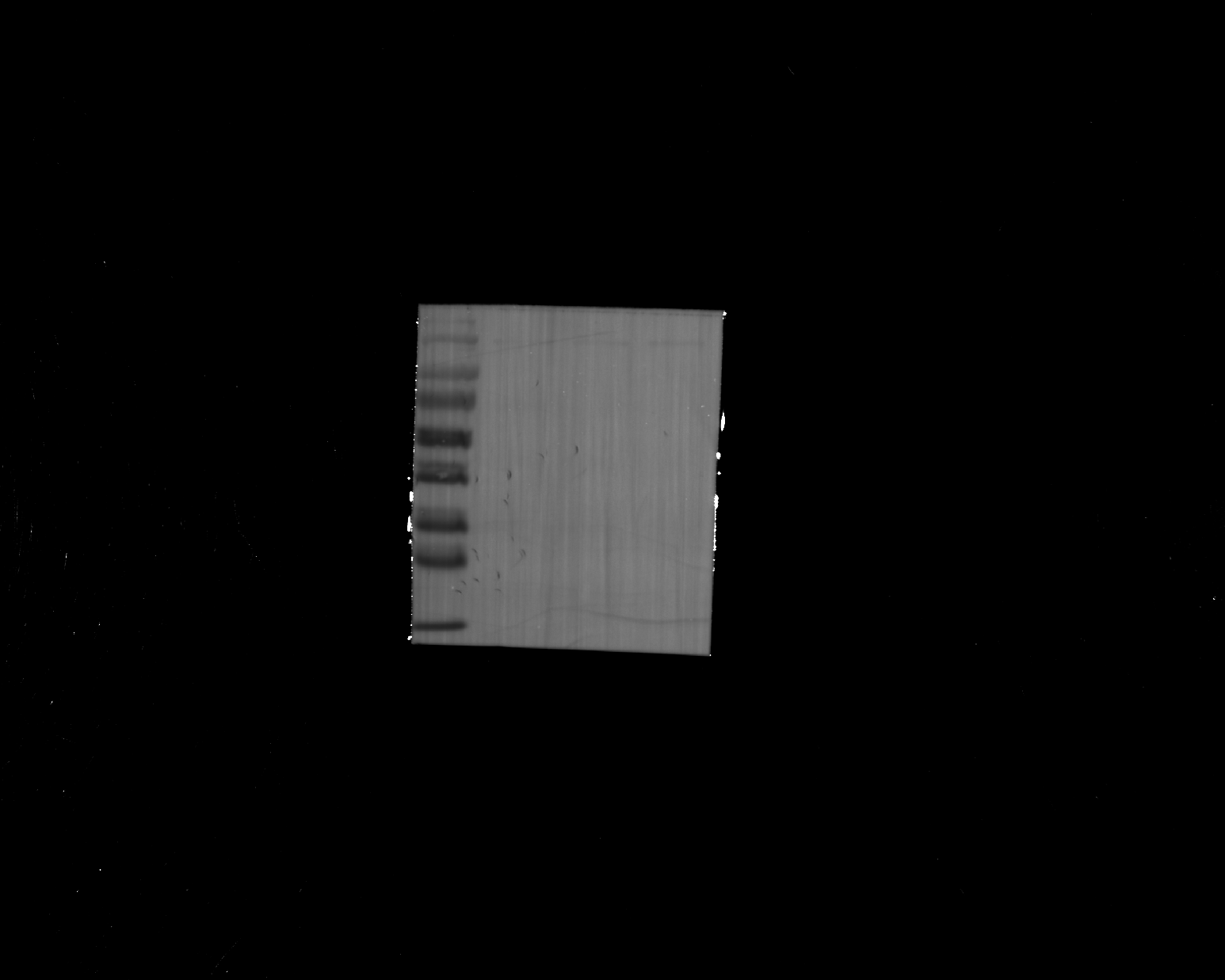

Supplement: Supplemental Information 5 [file peerj-12-18324-s005.zip › pstat6+stat6 1_2(Colorimetric).tif]

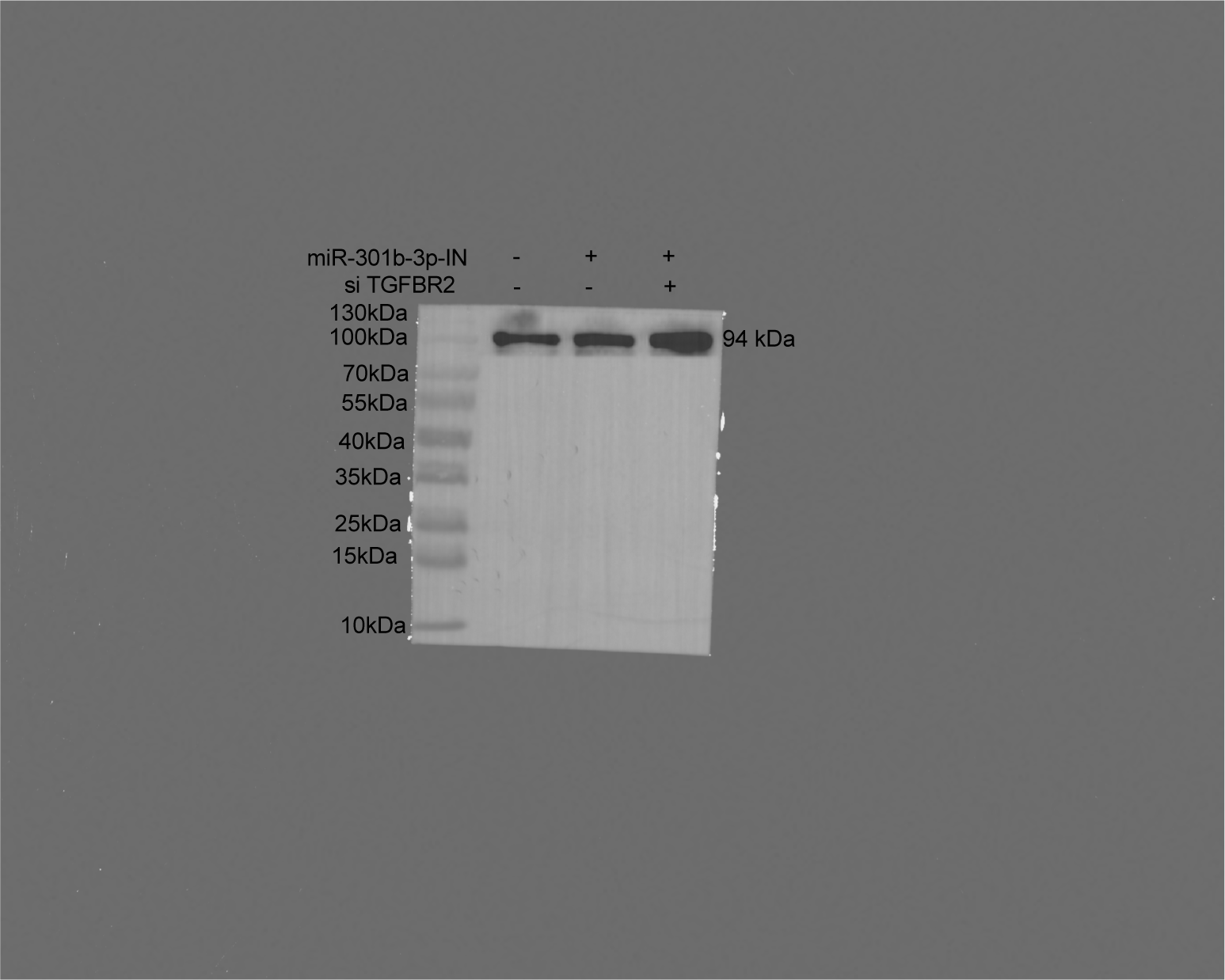

Supplement: Supplemental Information 5 [file peerj-12-18324-s005.zip › pstat6+stat6 1_2(Composite)-01.tif]

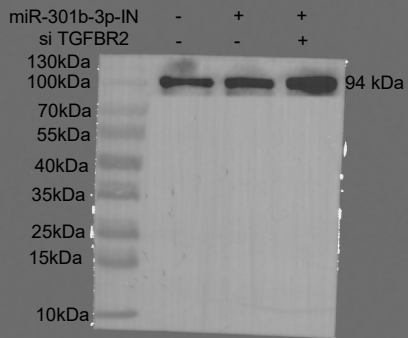

Supplement: Supplemental Information 5 [file peerj-12-18324-s005.zip › pstat6+stat6 1_2(Composite).pdf]

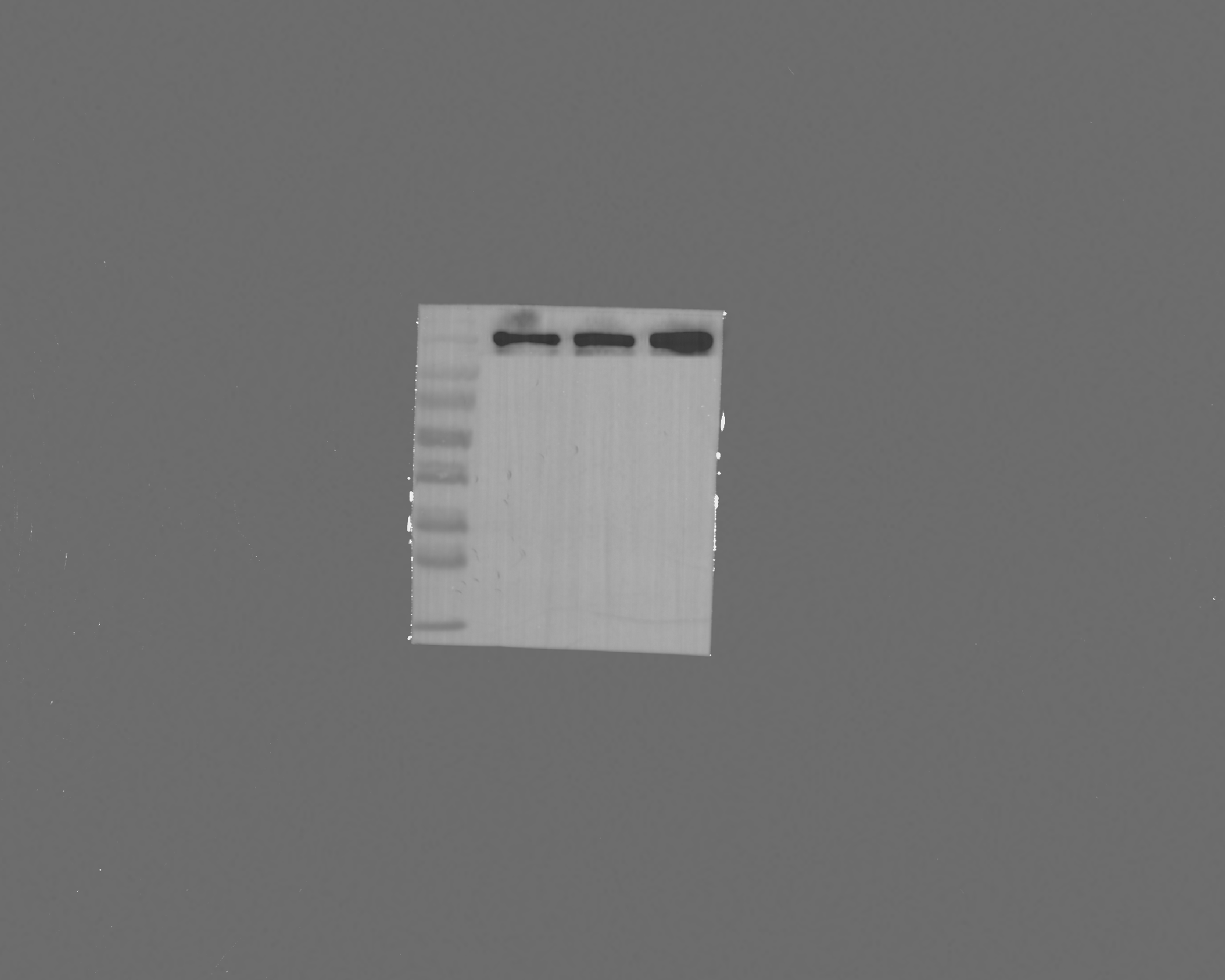

Supplement: Supplemental Information 5 [file peerj-12-18324-s005.zip › pstat6+stat6 1_2(Composite).tif]

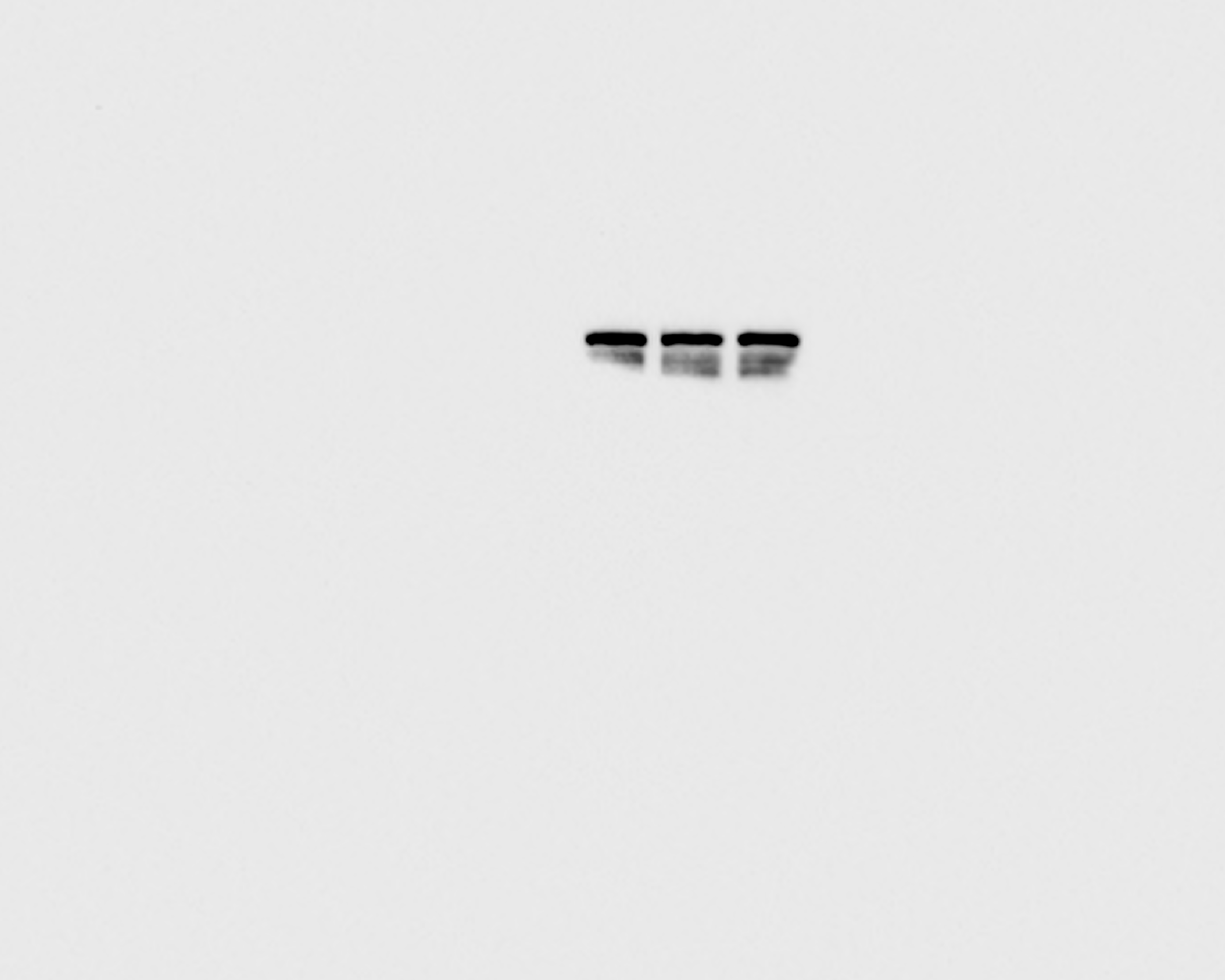

Supplement: Supplemental Information 5 [file peerj-12-18324-s005.zip › pstat6+stat6 2_1(Chemiluminescence).tif]

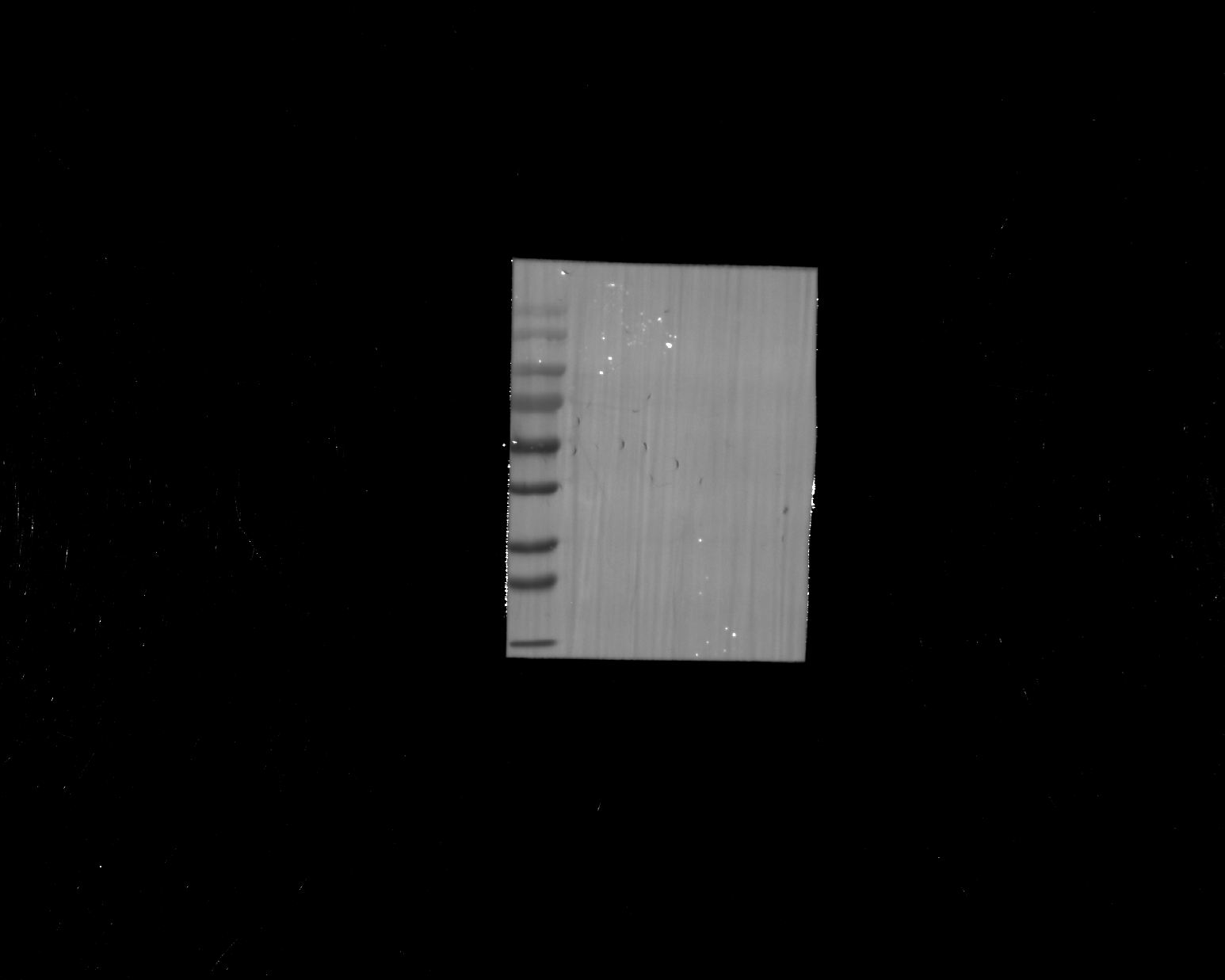

Supplement: Supplemental Information 5 [file peerj-12-18324-s005.zip › pstat6+stat6 2_1(Colorimetric).tif]

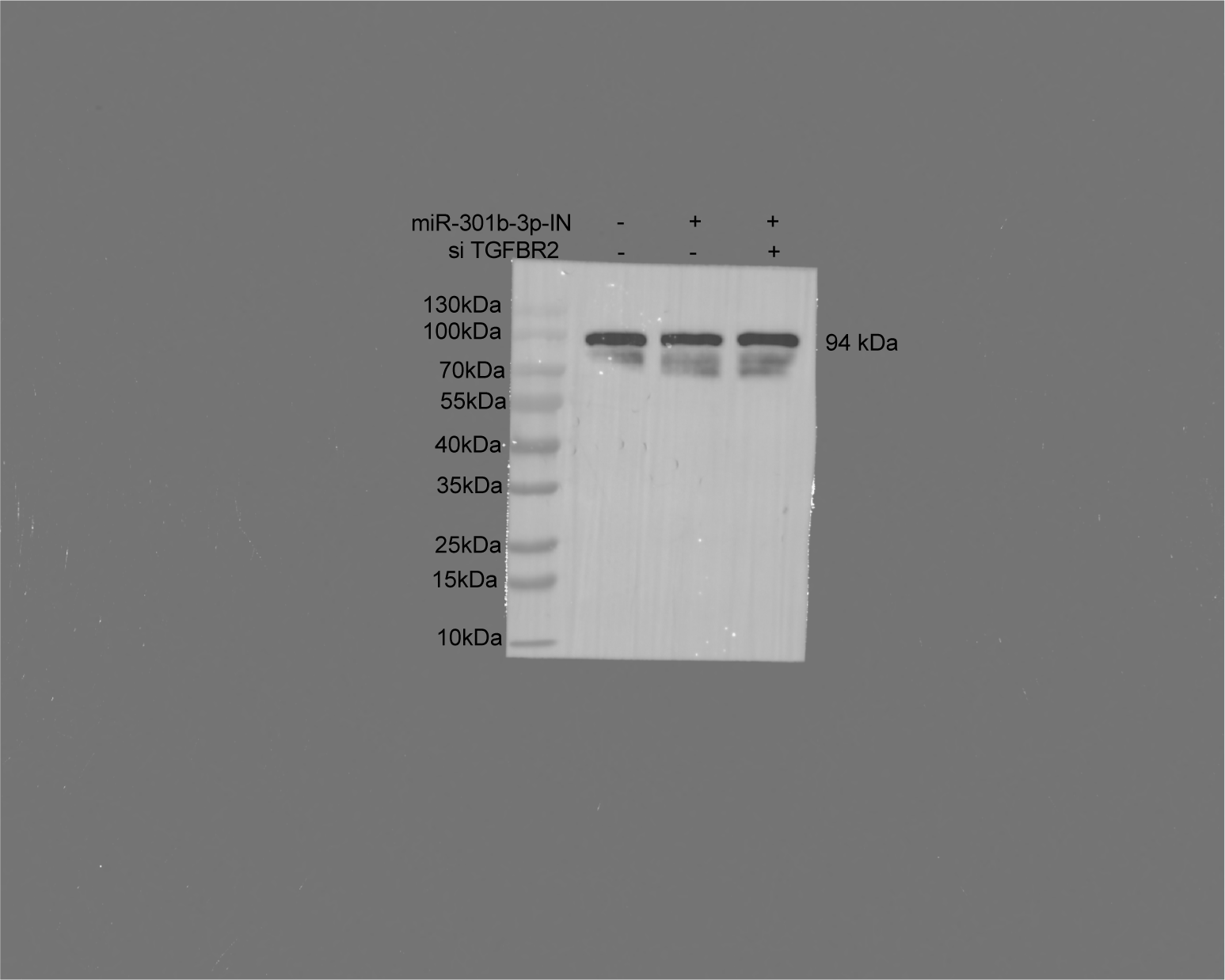

Supplement: Supplemental Information 5 [file peerj-12-18324-s005.zip › pstat6+stat6 2_1(Composite)-01.tif]

|                |   |   |   |
|----------------|---|---|---|
| miR-301b-3p-IN | - | + | + |
| si TGFBR2      | - | - | + |

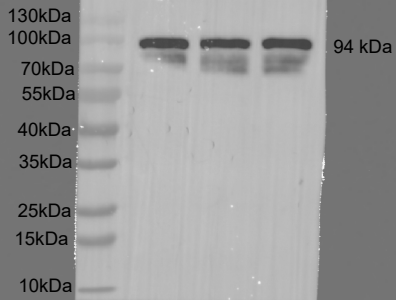

Supplement: Supplemental Information 5 [file peerj-12-18324-s005.zip › pstat6+stat6 2_1(Composite).pdf]

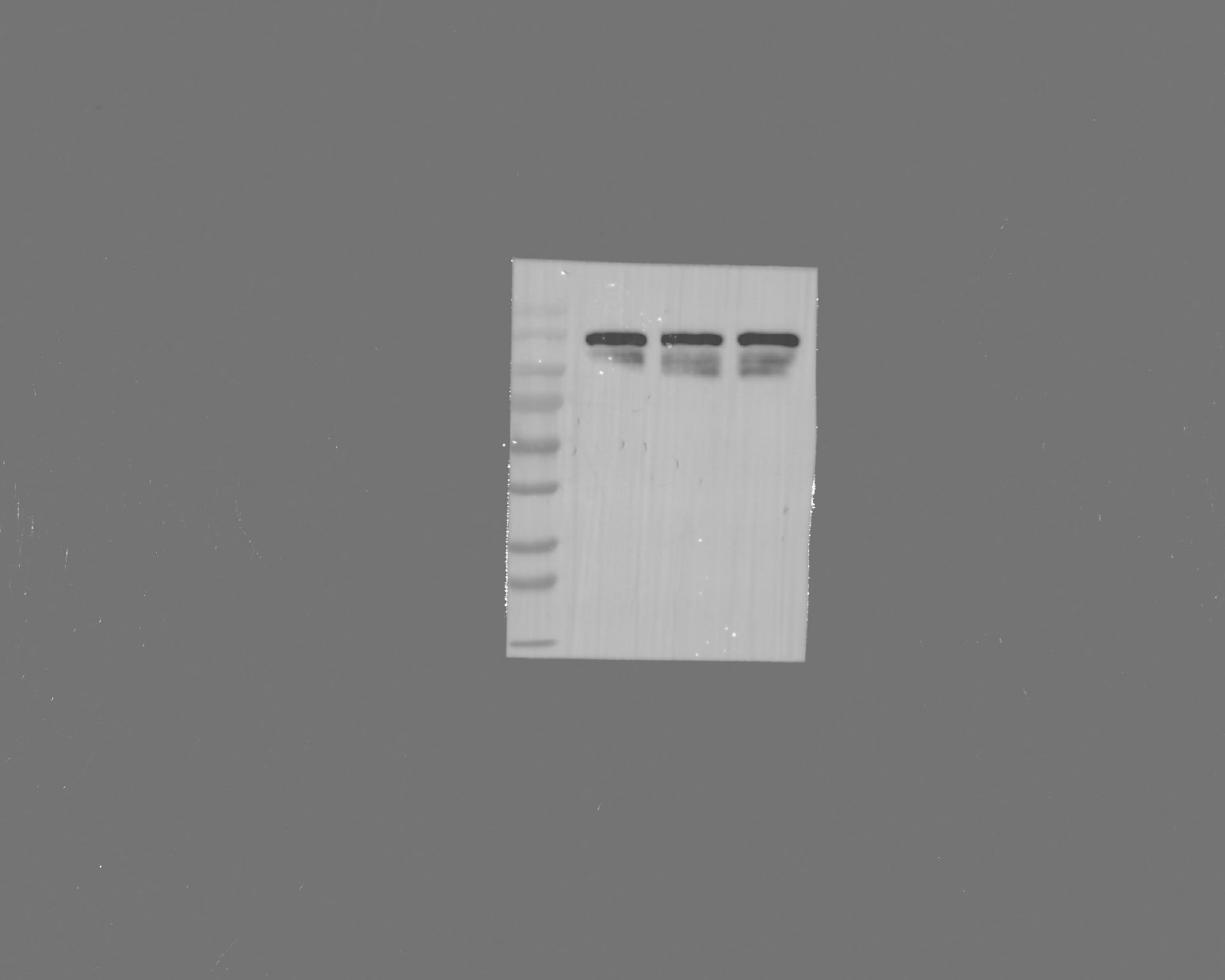

Supplement: Supplemental Information 5 [file peerj-12-18324-s005.zip › pstat6+stat6 2_1(Composite).tif]

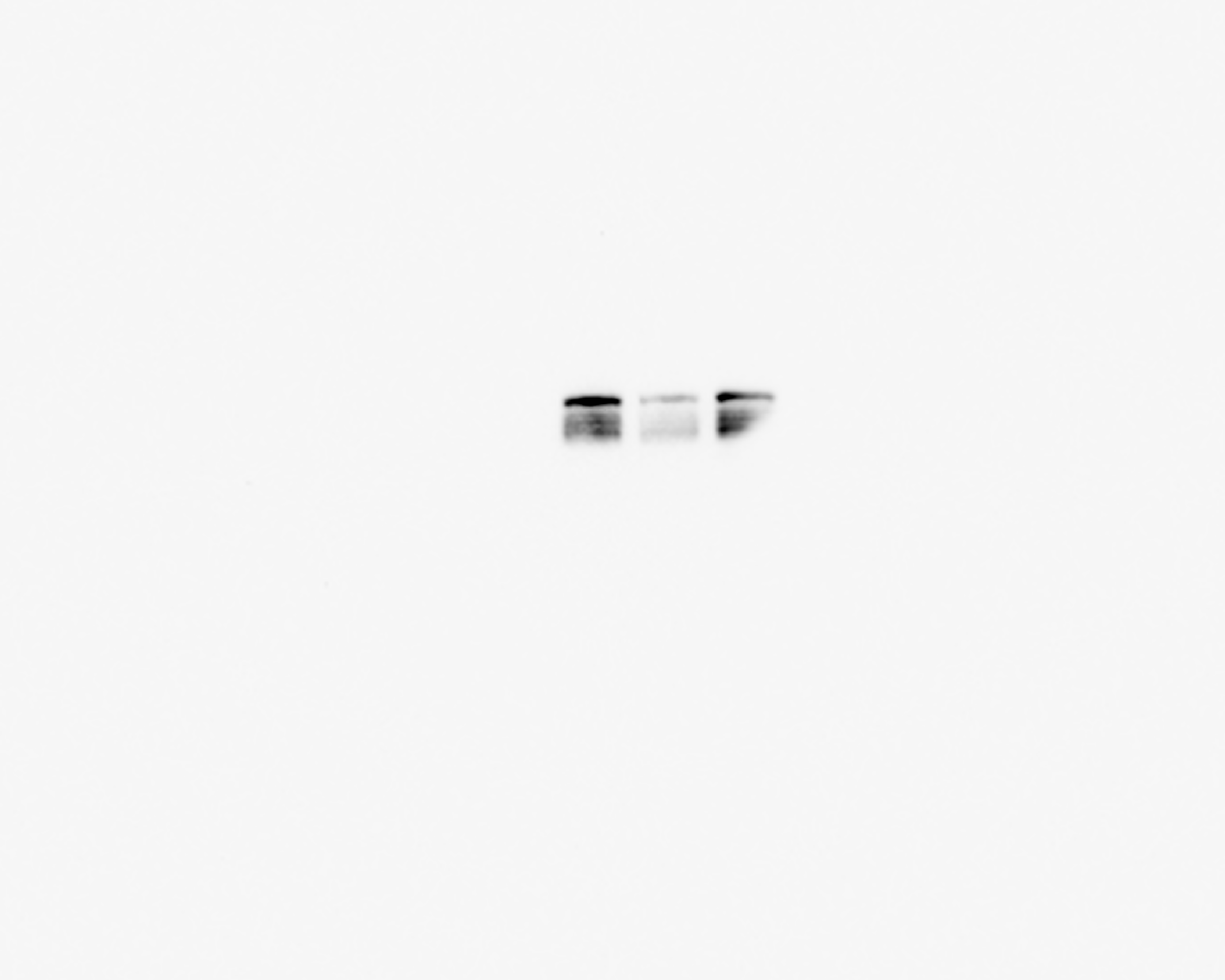

Supplement: Supplemental Information 5 [file peerj-12-18324-s005.zip › pstat6+stat6 2_2(Chemiluminescence).tif]

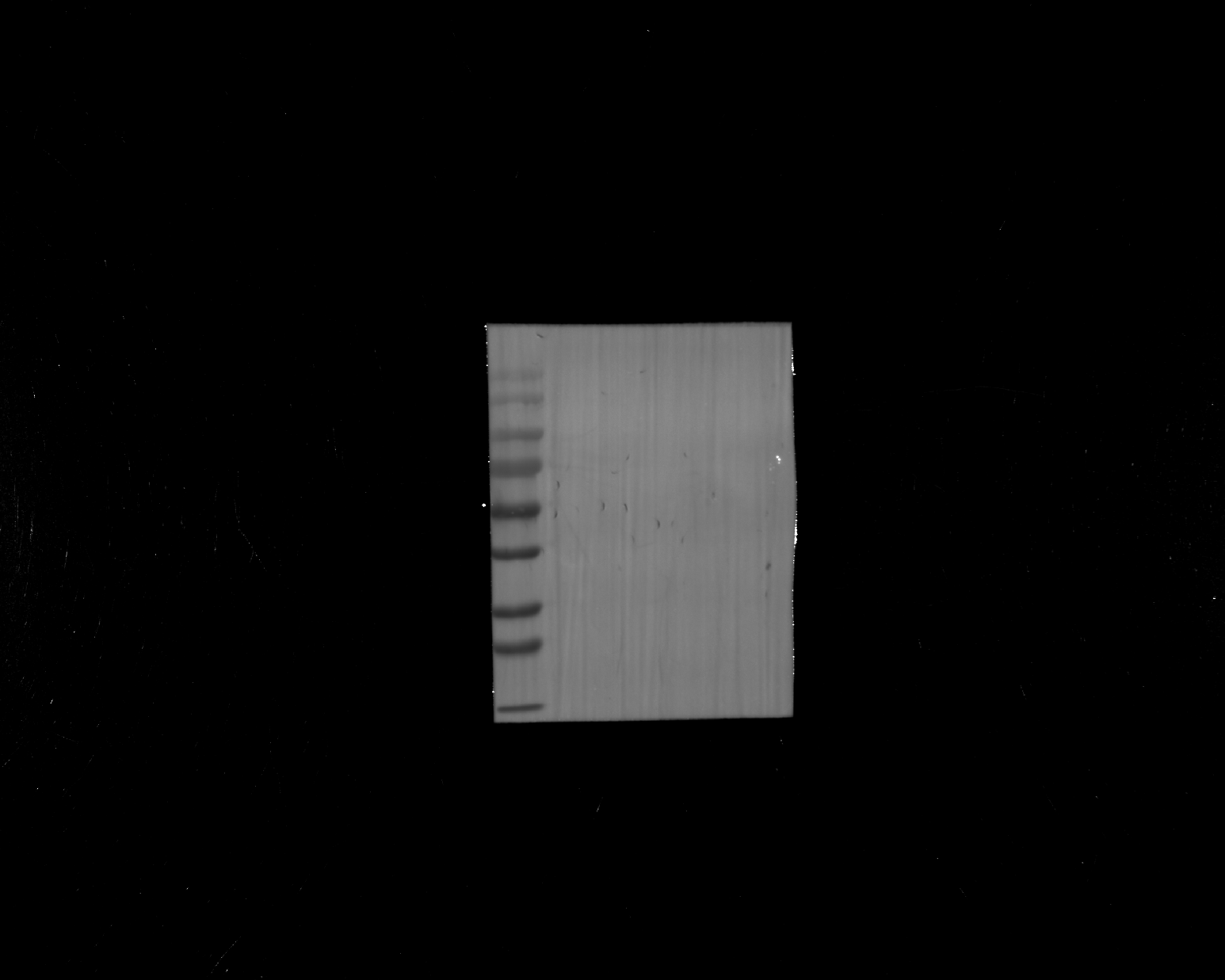

Supplement: Supplemental Information 5 [file peerj-12-18324-s005.zip › pstat6+stat6 2_2(Colorimetric).tif]

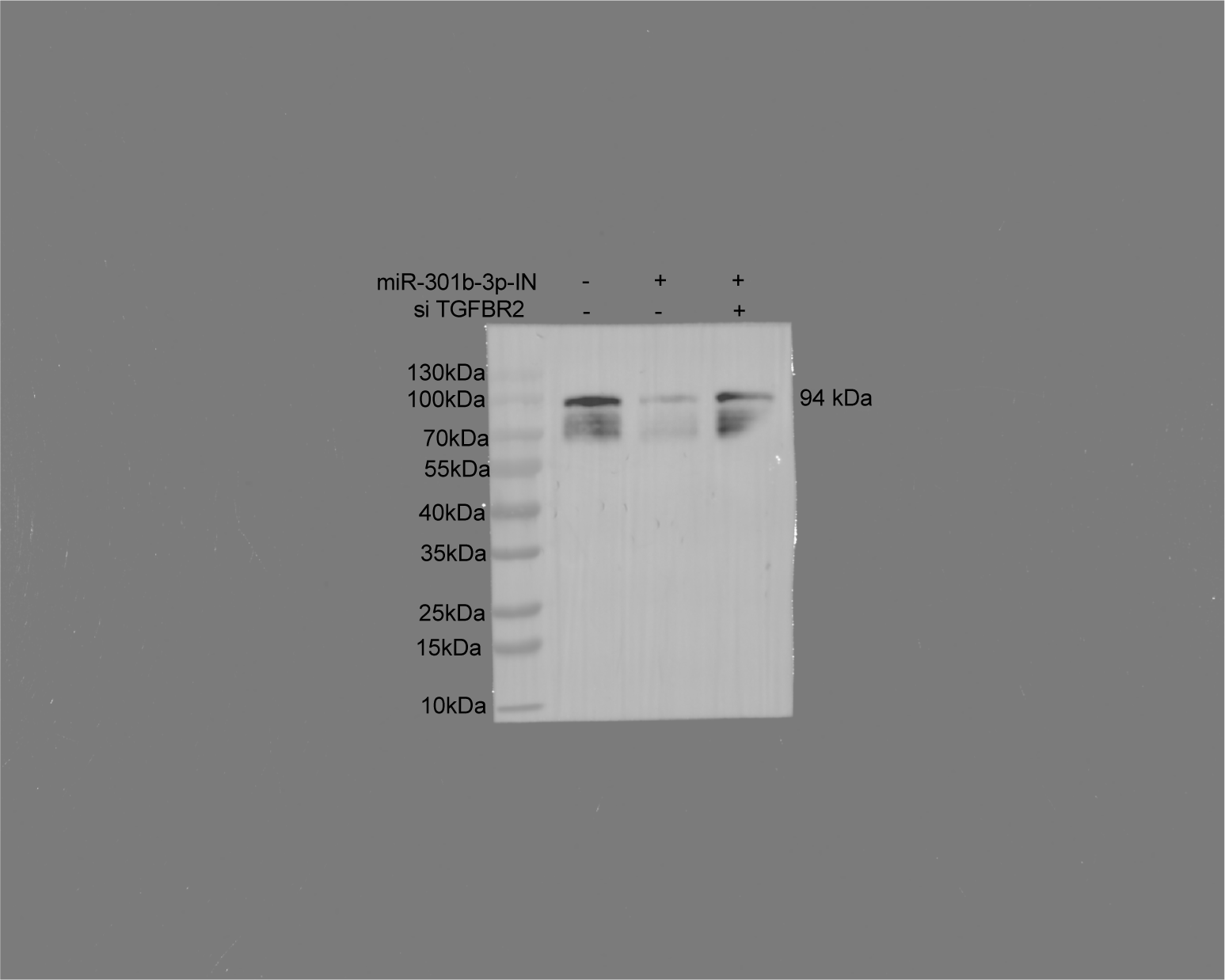

Supplement: Supplemental Information 5 [file peerj-12-18324-s005.zip › pstat6+stat6 2_2(Composite)-01.tif]

|                |   |   |   |
|----------------|---|---|---|
| miR-301b-3p-IN | - | + | + |
| si TGFBR2      | - | - | + |

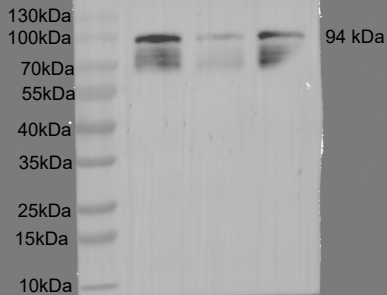

Supplement: Supplemental Information 5 [file peerj-12-18324-s005.zip › pstat6+stat6 2_2(Composite).pdf]

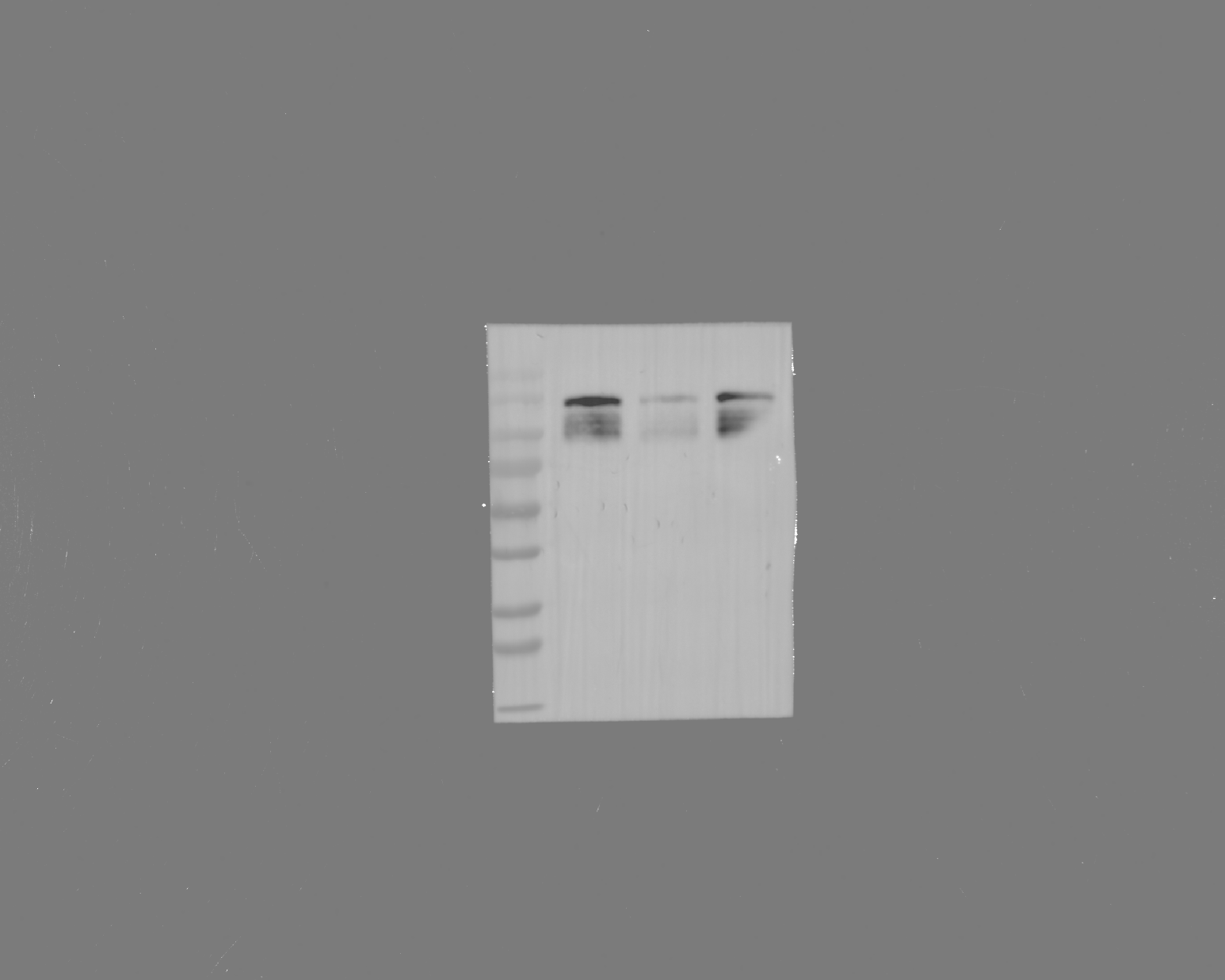

Supplement: Supplemental Information 5 [file peerj-12-18324-s005.zip › pstat6+stat6 2_2(Composite).tif]

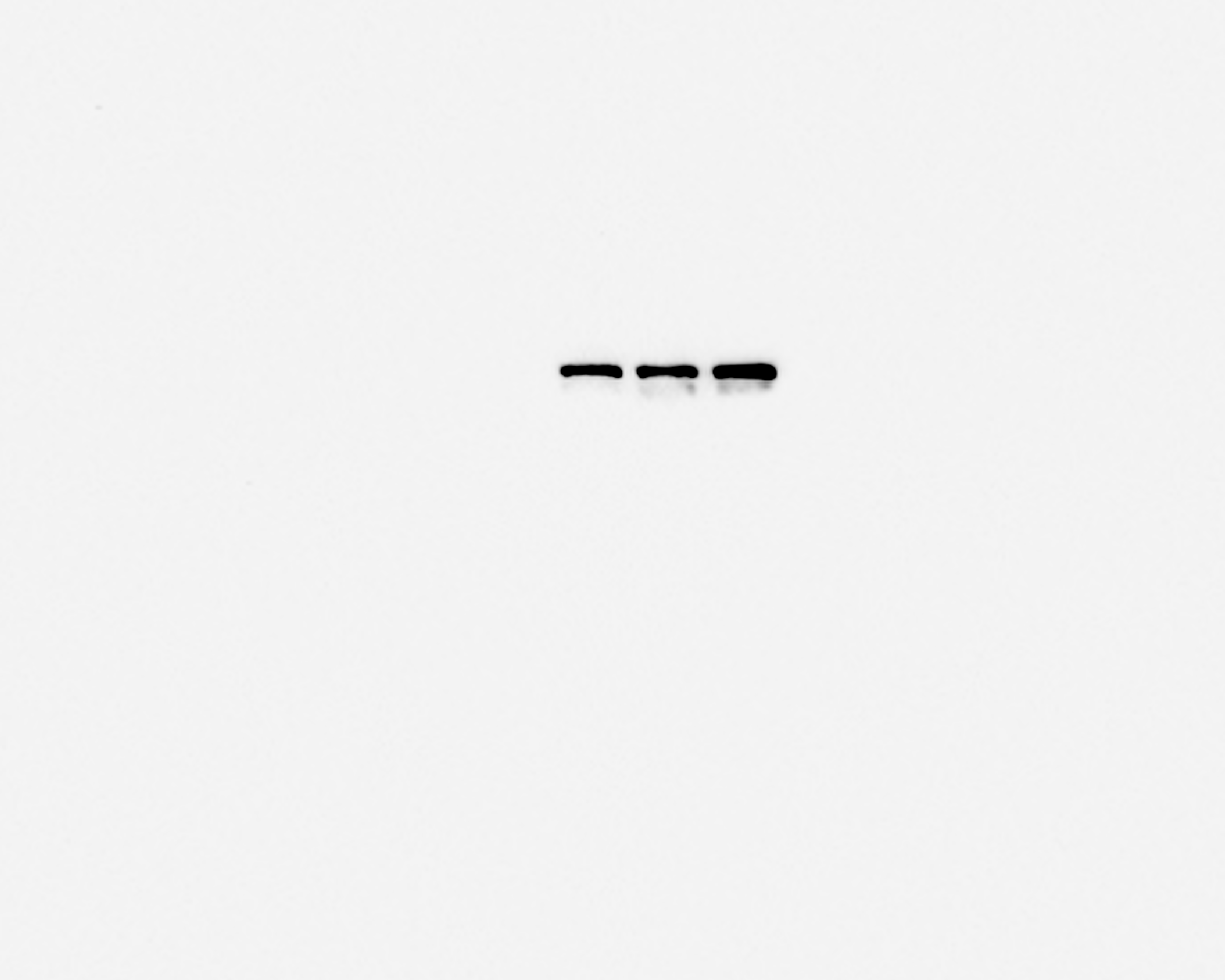

Supplement: Supplemental Information 5 [file peerj-12-18324-s005.zip › pstat6+stat6 3_1(Chemiluminescence).tif]

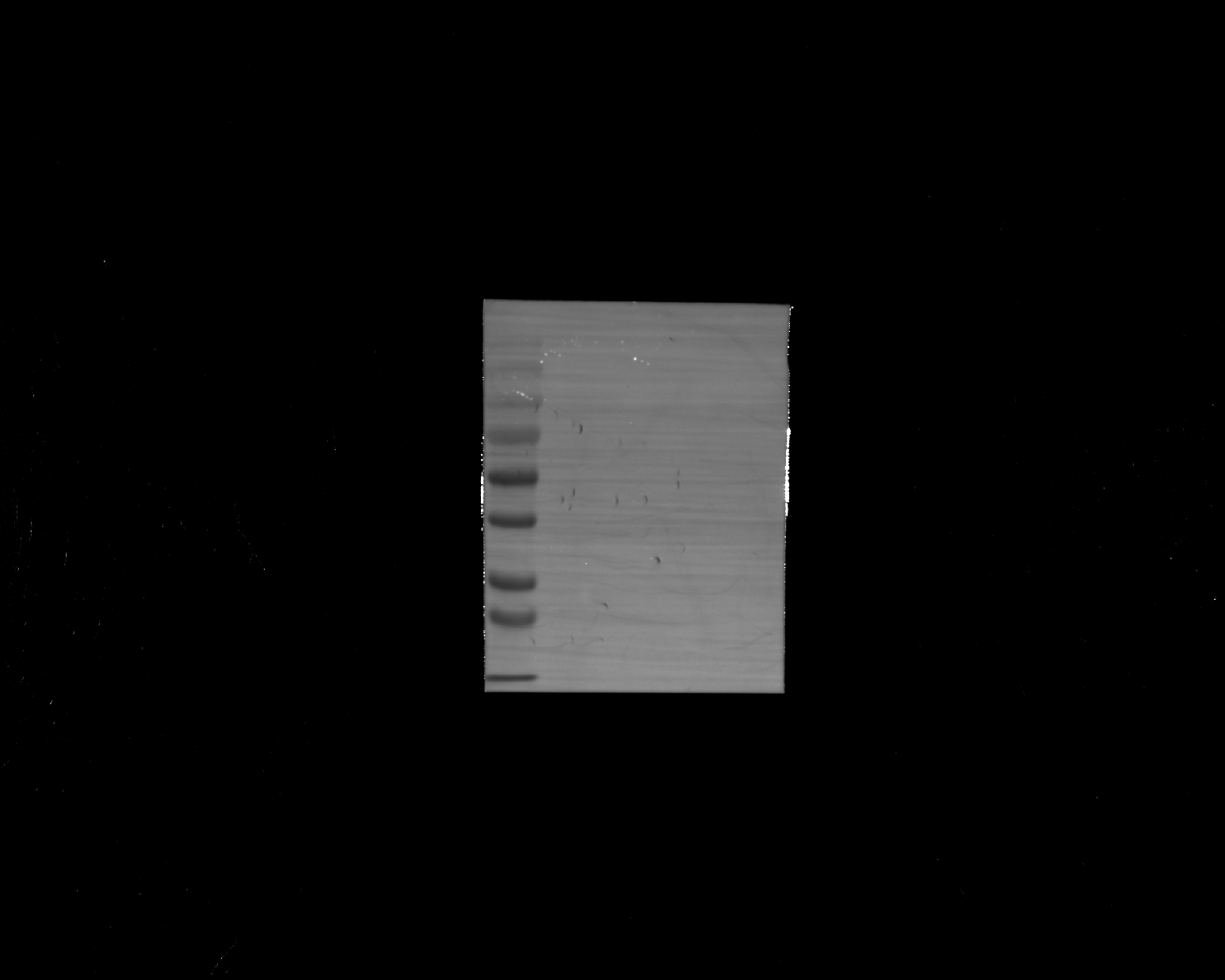

Supplement: Supplemental Information 5 [file peerj-12-18324-s005.zip › pstat6+stat6 3_1(Colorimetric).tif]

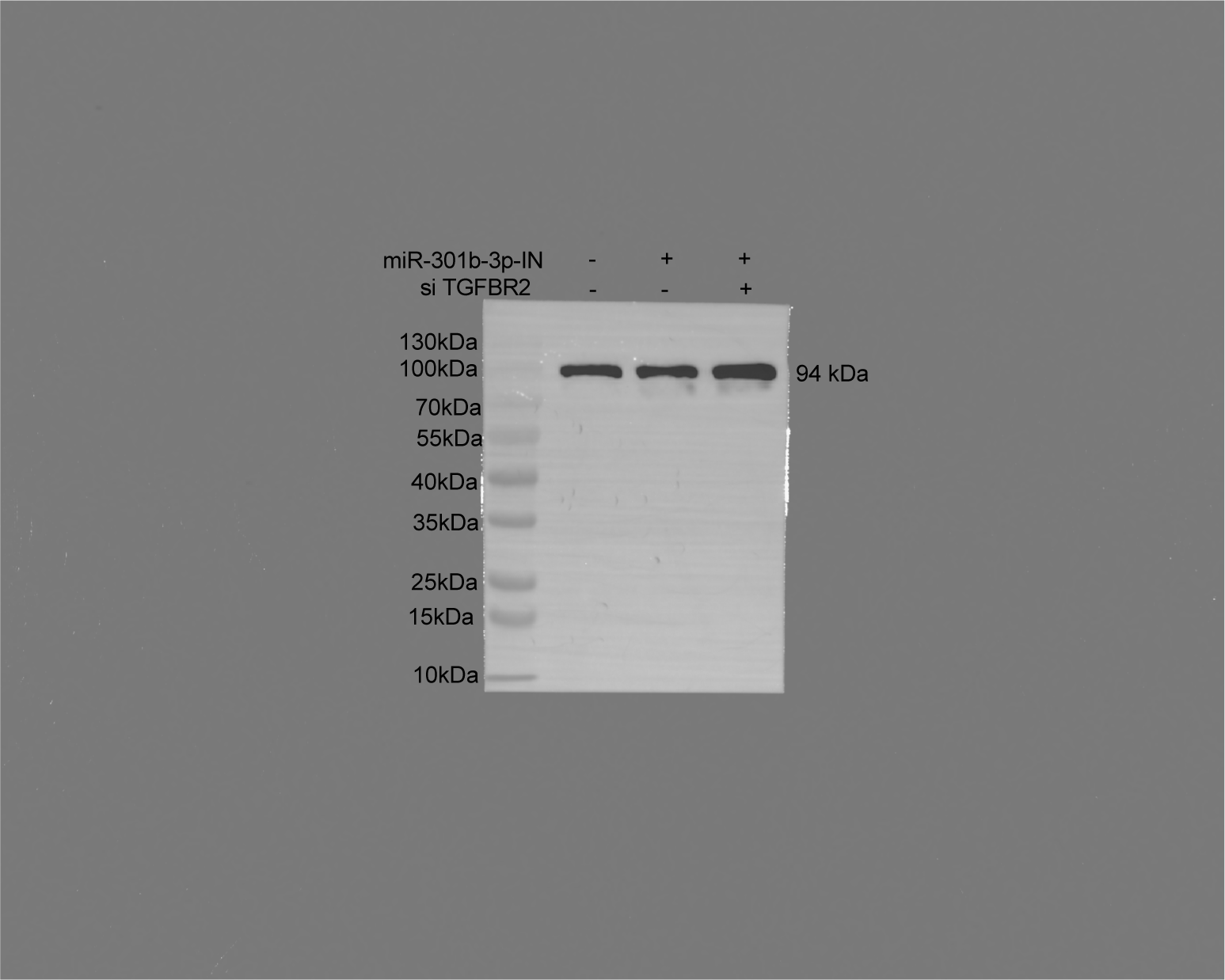

Supplement: Supplemental Information 5 [file peerj-12-18324-s005.zip › pstat6+stat6 3_1(Composite)-01.tif]

|                |   |   |   |
|----------------|---|---|---|
| miR-301b-3p-IN | - | + | + |
| si TGFR2       | - | - | + |

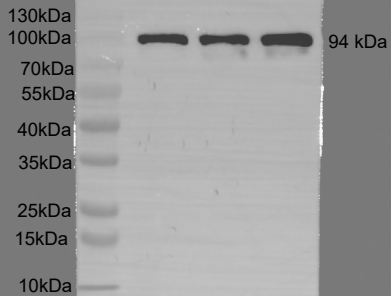

Supplement: Supplemental Information 5 [file peerj-12-18324-s005.zip › pstat6+stat6 3_1(Composite).pdf]

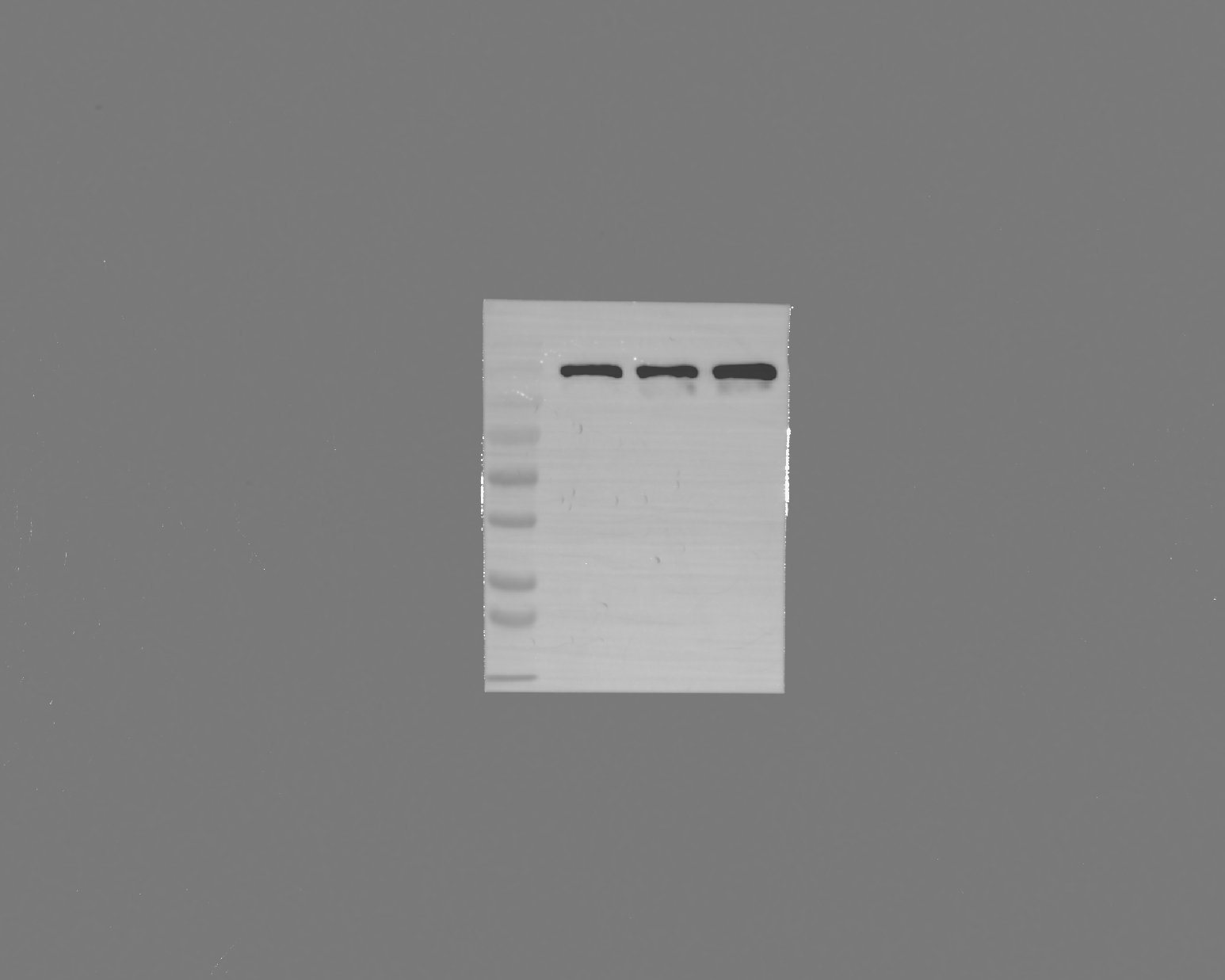

Supplement: Supplemental Information 5 [file peerj-12-18324-s005.zip › pstat6+stat6 3_1(Composite).tif]

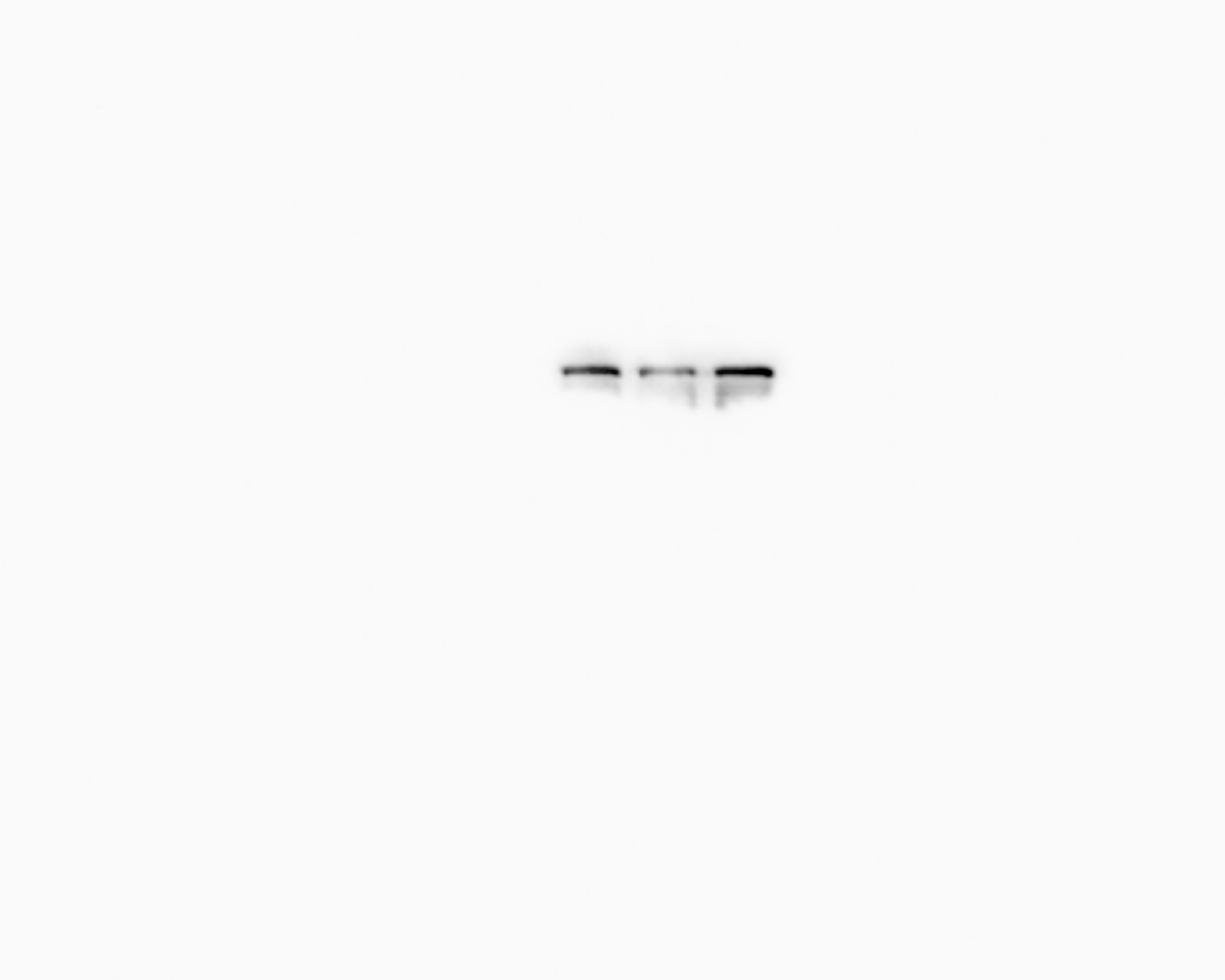

Supplement: Supplemental Information 5 [file peerj-12-18324-s005.zip › pstat6+stat6 3_2(Chemiluminescence).tif]

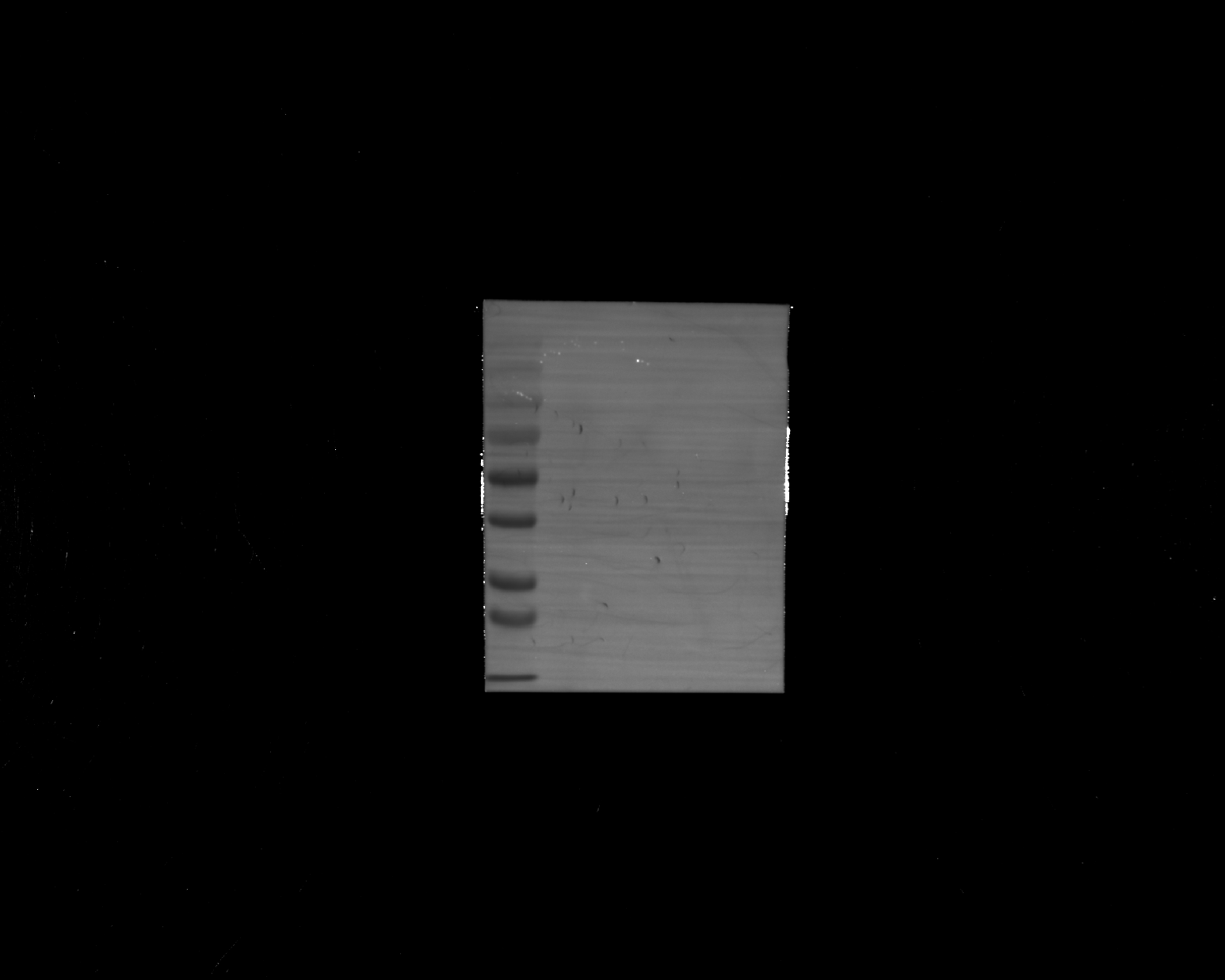

Supplement: Supplemental Information 5 [file peerj-12-18324-s005.zip › pstat6+stat6 3_2(Colorimetric).tif]

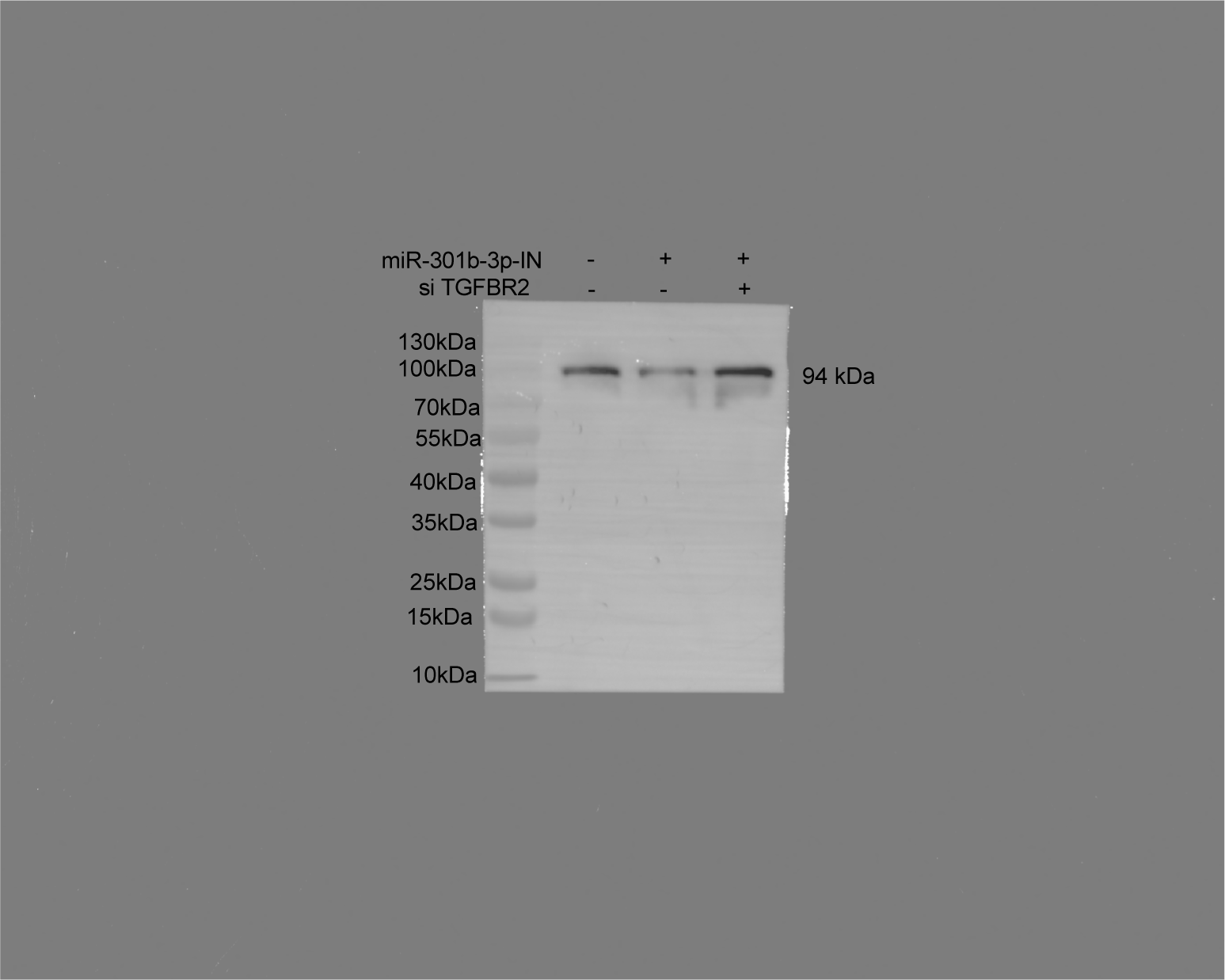

Supplement: Supplemental Information 5 [file peerj-12-18324-s005.zip › pstat6+stat6 3_2(Composite)-01.tif]

|                        |   |   |   |
|------------------------|---|---|---|
| miR-301b-3p-IN         | - | + | + |
| si TGFB <sup>R</sup> 2 | - | - | + |

130kDa

100kDa

70kDa

55kDa

40kDa

35kDa

25kDa

15kDa

10kDa

94 kDa

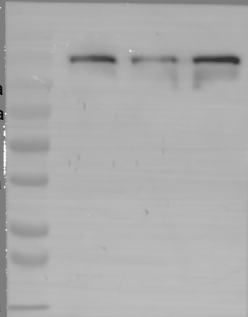

Supplement: Supplemental Information 5 [file peerj-12-18324-s005.zip › pstat6+stat6 3_2(Composite).pdf]

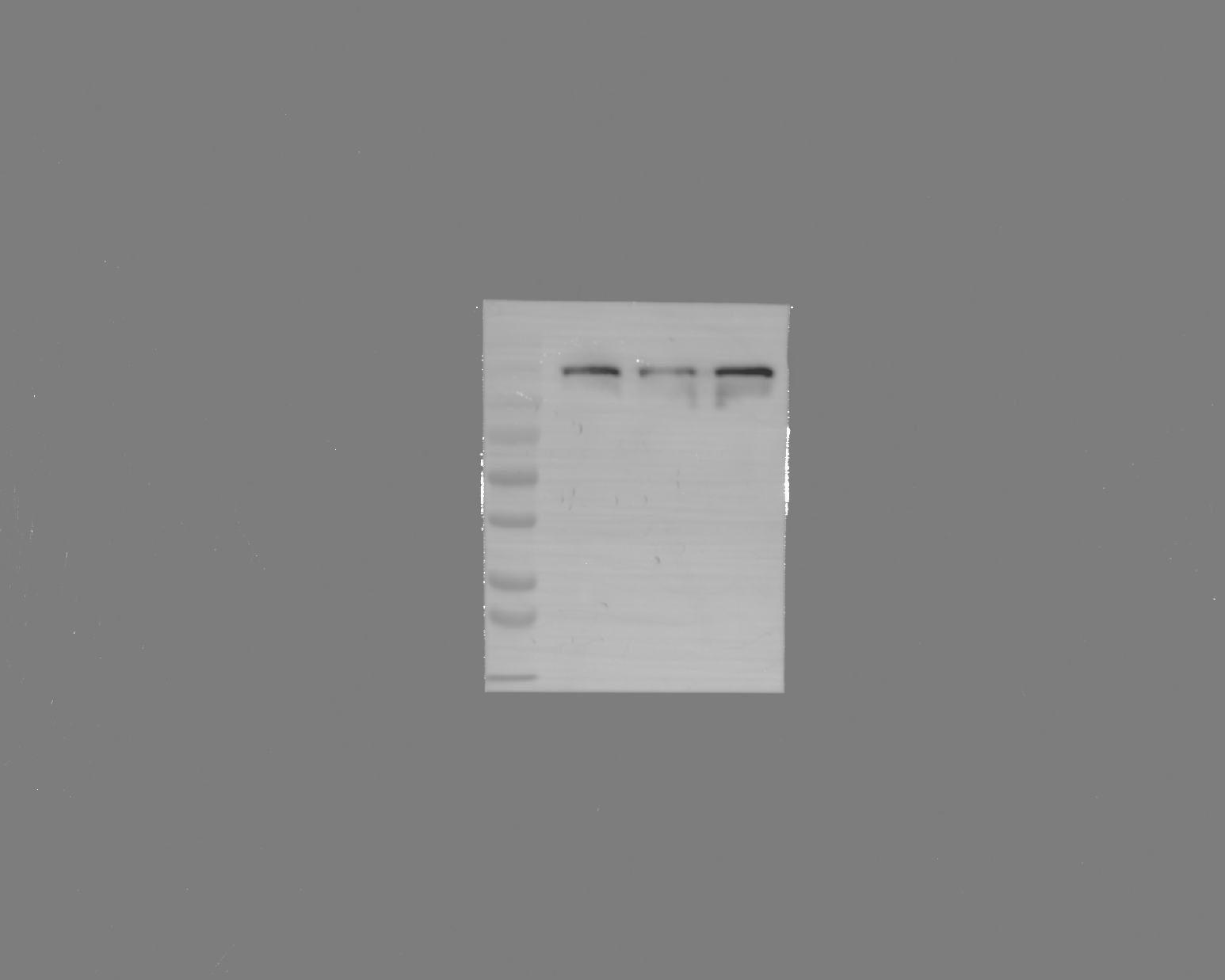

Supplement: Supplemental Information 5 [file peerj-12-18324-s005.zip › pstat6+stat6 3_2(Composite).tif]
